# Genetic Structure of Avian Influenza Viruses from Ducks of the Atlantic Flyway of North America

**DOI:** 10.1371/journal.pone.0086999

**Published:** 2014-01-30

**Authors:** Yanyan Huang, Michelle Wille, Ashley Dobbin, Natasha M. Walzthöni, Gregory J. Robertson, Davor Ojkic, Hugh Whitney, Andrew S. Lang

**Affiliations:** 1 Department of Biology, Memorial University of Newfoundland, St. John's, Newfoundland and Labrador, Canada; 2 Wildlife Research Division, Environment Canada, Mount Pearl, Newfoundland and Labrador, Canada; 3 Animal Health Laboratory, University of Guelph, Guelph, Ontario, Canada; 4 Newfoundland and Labrador Department of Natural Resources, St. John's, Newfoundland and Labrador, Canada; University of Georgia, United States of America

## Abstract

Wild birds, including waterfowl such as ducks, are reservoir hosts of influenza A viruses. Despite the increased number of avian influenza virus (AIV) genome sequences available, our understanding of AIV genetic structure and transmission through space and time in waterfowl in North America is still limited. In particular, AIVs in ducks of the Atlantic flyway of North America have not been thoroughly investigated. To begin to address this gap, we analyzed 109 AIV genome sequences from ducks in the Atlantic flyway to determine their genetic structure and to document the extent of gene flow in the context of sequences from other locations and other avian and mammalian host groups. The analyses included 25 AIVs from ducks from Newfoundland, Canada, from 2008–2011 and 84 available reference duck AIVs from the Atlantic flyway from 2006–2011. A vast diversity of viral genes and genomes was identified in the 109 viruses. The genetic structure differed amongst the 8 viral segments with predominant single lineages found for the PB2, PB1 and M segments, increased diversity found for the PA, NP and NS segments (2, 3 and 3 lineages, respectively), and the highest diversity found for the HA and NA segments (12 and 9 lineages, respectively). Identification of inter-hemispheric transmissions was rare with only 2% of the genes of Eurasian origin. Virus transmission between ducks and other bird groups was investigated, with 57.3% of the genes having highly similar (≥99% nucleotide identity) genes detected in birds other than ducks. Transmission between North American flyways has been frequent and 75.8% of the genes were highly similar to genes found in other North American flyways. However, the duck AIV genes did display spatial distribution bias, which was demonstrated by the different population sizes of specific viral genes in one or two neighbouring flyways compared to more distant flyways.

## Introduction

Epidemiological surveillance of avian influenza viruses (AIVs) has revealed a huge and dynamic virus reservoir in wild birds, especially in ducks. The genetic diversity of AIVs in wild birds is shaped by many factors such as the high error rate of the replicase, the segmented nature of the viral genome, broad host range properties of the viruses, and bird migration patterns [1]–[3]. Decades of studies in wild birds have revealed seasonal patterns of viral prevalence [2] and the segregation of AIV sequences into Eurasian and North American [2] and now also South American geographic phylogenetic lineages [4], [5].

The extensive AIV genome sequences accumulated during recent decades has made it possible to study the patterns of intra-continental virus distribution in North America. Analysis of the duck AIV genomes available from 1976 to 2005 showed strong patterns of AIV distribution by sampling locations and time, but not by duck species [6]. A large-scale statistical phylogeographic investigation of AIVs demonstrated a strong link between AIV phylogeny, spatial distance and migratory flyway [7]. Another recent large-scale analysis did not find that flyway separation affected the distribution of AIVs over long periods [8], supporting extensive movement of viruses between regions in North America. Recent work with ducks in Alaska and California has provided important insights into AIV ecology at the western edge of North America. Comparison of AIVs from these two locations from 2006 to 2008 revealed the transmission of AIVs was strongly associated with duck species, locations and sampling times [9]. Comparison of 161 Alaskan dabbling duck AIV genomes from 2005 to 2008 detected inter-species transmission of viruses between Northern Pintail (*Anas acuta*), Mallard (*A. platyrhynchos*), American Green-winged Teal (*A. carolinensis*) and Northern Shoveler (*A. clypeata*) during all sampling years [10]. Northern Pintails in Alaska have shown a higher inter-continental AIV detection rate compared to other duck species in Alaska as well as ducks from other locations of North America [11]–[14]. Mallards in Alaska also carry inter-continental AIV reassortants but, unlike Northern Pintails that move between Alaska and Asia, these are due to secondary infection from other bird species [15].

In comparison to the large amount of work performed in Pacific North America, our knowledge of the ecology of AIVs in ducks in eastern North America and the Atlantic flyway, especially at the northern end in Atlantic Canada, is more limited. The province of Newfoundland and Labrador, Canada, is located at the northeastern margin of North America; yet, the role of ducks from this region, and the Atlantic flyway in general, in intra- and inter-hemispheric AIV transmission is understudied. We have analyzed AIV genome sequence data from ducks on the island of Newfoundland, Canada, along with available reference Atlantic flyway duck virus sequences from 2006–2011 to increase our knowledge of AIV dynamics in this region of North America. We have used these data to examine the genetic structure in these viruses and to determine the amount of gene flow amongst different bird groups and North American migratory flyways.

## Materials and Methods

### Ethics statement

The samples used in this study came from ducks (*Anas* spp.) that were caught by bait trapping and banded under banding permit 10559 from Environment Canada at public locations requiring no access permits. This study did not involve endangered or protected species. Swabs of the duck oropharyngeal cavity and cloaca were collected. This work was carried out under the guidelines specified by the Canadian Council on Animal Care with approved protocols 09-01-AL, 10-01-AL, and 11-01-AL from the Memorial University Institutional Animal Care Committee, and biosafety permit S-103-08 from the Memorial University Biosafety Committee.

### Determination of AIV genome sequences from ducks in Newfoundland, Canada

Genome sequence data were successfully acquired for twenty-five AIVs from ducks in the St. John's region of Newfoundland, Canada, that were identified during AIV epidemiological surveillance over 2008–2011 [16] (Table S1). RNA was extracted from the swabs, which were kept cool in the field after collection and then stored at −80°C until use, with Trizol LS (Life Technologies, Burlington, Canada) and the AIV sequences were amplified by RT-PCR using the SuperScript® III One-Step RT-PCR System with Platinum *Taq* (Life Technologies) and previously published primers [17]–[21]. The resulting PCR products were purified with the QIAquick PCR purification kit (Qiagen, Toronto, Canada), and sequenced in both directions by Sanger sequencing at the Centre for Applied Genomics (Hospital for Sick Children, Toronto, Canada). Samples that showed evidence of mixed sequences were excluded. The nucleotide sequence data were compiled and analyzed using the Lasergene v7.1 sequence analysis software package (DNASTAR Inc., Madison, WI). The GenBank accession numbers for the sequences are KC492244-KC492440.

### Reference AIV genome sequences from ducks in the Atlantic flyway

Eighty-four reference AIV genomes, representing all available sequences (as of August 2012) from ducks in the Atlantic flyway over the period of 2006 to 2011, were downloaded from the NCBI influenza database [22]. The numbers of viruses from different locations were: Newfoundland, 7; Quebec, 20; New Brunswick, 32; Prince Edward Island, 9; Ontario, 1; Nova Scotia, 2; New York, 4; Pennsylvania, 1; Delaware, 1; Maryland, 6; and Florida, 1 (Table S2).

### Reference AIV genome sequences from other North American flyways

The genome sequences of duck AIVs from 2006 to 2011 from the Pacific, Central and Mississippi flyways were analyzed for their genotypes in the Flugenome database (http://www.flugenome.org/), which employs a nucleotide difference of 10% (p-distance analysis) as the cut-off value for an individual gene lineage, with the genotype of a certain AIV determined by the combination of gene lineages for segments 1 to 8 [23]. Viral genome sequences from different flyways representing all available genotype varieties were downloaded (1 genome per genotype in each bird flyway) from the NCBI influenza database for use in comparisons with the Atlantic flyway viruses. This provided 141 reference duck AIV genomes, with 102 genotypes from the Pacific flyway (35 from Alaska and 67 from California), 13 genotypes from the Central flyway, 21 genotypes from the Mississippi flyway, and 5 genotypes from Guatemala (a location of overlapping flyways). In addition, AIV gene segments from host species other than ducks that were highly related (≥99% nucleotide identity) to those in the Atlantic flyway duck AIVs were also downloaded from the NCBI database to study the distribution of the viral genes in different host groups.

### Phylogenetic analysis of the Atlantic flyway duck AIVs

Two sets of phylogenetic trees were constructed for each segment. The first set included 250 duck AIV genome sequences, 109 from the Atlantic flyway and 141 from other bird flyways in North America and Guatemala, to compare the phylogenetic features of the Atlantic flyway sequences to those from the other flyways. The second set was performed to determine the occurrence of the Atlantic flyway genes in non-duck (avian and mammal) host species. The nucleotide sequence alignments and pairwise distance (p-distance) analyses were performed with the Jotun Hein method in Lasergene. Phylogenetic trees were constructed with the neighbour-joining method in MEGA5 [24]. The nucleotide ranges of the coding regions used for each gene were: PB2, 1804–2256 bp; PB1, 1624–2271 bp; PA, 1–528 bp; NP, 4–576 bp; M, 40–939 bp; NS, 55–792 bp; H1, 1147–1590 bp; H2, 1180–1689 bp; H3, 1102–1680 bp; H4, 1120–1659 bp; H5, 1086–1686 bp; H6, 1018–1665 bp; H7, 691–1659 bp; H11, 1087–1656 bp; H12, 1123–1662 bp; H13, 493–1695 bp; H16, 13–1668 bp; N1, 910–1344 bp; N2, 1102–1410 bp; N3, 760–1347 bp; N4, 1004–1392 bp; N6, 904–1383 bp; N8, 856–1404 bp; N9, 844–1395 bp.

### Gene lineage and genotype assignments of the Atlantic flyway duck AIVs

On the basis of the phylogenetic trees, the 109 Atlantic flyway duck AIVs were analyzed to categorize their gene lineages and genotypes following the nomenclature of the Flugenome database [23]. The sequences were also classified by geographic and host group lineage (i.e. North American avian, Eurasian avian, North American gull or Eurasian gull) according to the origins of the reference sequences in the same phylogenetic clades. To study the genetic structure of the Atlantic flyway duck AIV population at an even finer level, gene sub-lineages were further determined with the criterion of ≥95% nucleotide identity (p-distance) for a sub-lineage. Sub-genotypes of the viruses were then determined accordingly. We employed the Flugenome gene lineage and genotype determination approach in this study because it works well as a universal phylogenetic classification method for AIV genes and virus genotyping [23], [25]. This was chosen to allow representation of the genetic structure of the viruses in a standardized way that also makes it easier to directly compare between different studies.

### Identification of highly similar genes and genomes among the Atlantic flyway duck AIVs

To study the patterns of distribution and maintenance of the Atlantic flyway duck AIVs, highly related genes and genomes were identified within the identified gene sub-lineages and virus sub-genotypes. Sequences with nucleotide identity of ≥99% (p-distance) were considered as highly similar genes or the same gene type. Viruses with nucleotide identities ≥99% for all 8 segments were considered as homologous genomes [10]. The identified highly similar genes and genomes were analyzed for their spatial and temporal patterns of detection.

### Investigation of the distribution of Atlantic flyway duck AIVs through space, time and host species

Each of the 248 gene types identified in the Atlantic flyway duck AIVs was used to search the NCBI database for highly related reference genes by BLAST (nucleotide identity ≥99% and coverage ≥95%) [26]. The longest gene sequence of the same gene type was chosen as the query sequence when applicable. The sequences highly related to the Atlantic flyway sequences were categorized for detection date, location and host species.

Differences among proportions of gene types detected for >5 and >10 years for the eight segments were analysed using standard χ^2^ contingency table tests. Individual contributions of each segment to the overall χ^2^ test were examined, and deemed to be significant different from the overall proportion when the χ^2^ was greater than the critical χ^2^ value for α, where α was set to 0.05 and adjusted downward for multiple comparisons (0.05/8 segments  = 0.0063). All tests were 2-tailed and statistics were calculated using R 3.0.1 (www.R-project.org/).

## Results

### Phylogenetic analysis, gene typing and genotyping of the Atlantic flyway duck AIVs

Phylogenetic trees of the 109 Atlantic flyway virus sequences and the 141 reference virus sequences were constructed (Figure S1). This allowed delineation of the Atlantic flyway sequences into 34 gene lineages, 76 sub-lineages and 248 different gene types (summarized in Table 1; full details provided in Table S2). Of the 248 gene types, 235 (95.5%) were of North American avian origin. The other 13 gene types, from 6 different viruses, represented 11 gull-related genes and 2 Eurasian avian genes (Table 2). At the genomic level, 43 different genotypes and 70 sub-genotypes were identified amongst the 109 viruses (Table S2). These high levels of diversity were not artifacts of the large geographical representation over the flyway because diverse gene types (Table S3) and genotypes (Table S4) were also observed in the individual locations contributing ≥20 AIVs (Newfoundland, Quebec and New Brunswick; Table S4).

**Table 1:** Summary of gene lineage and gene type diversity by segment for the 109 Atlantic flyway duck AIVs from 2006–2011.

Category^a^	HA	NA	PB2	PB1	PA	NP	M	NS	Total
Gene lineages	12	9	2	1	2	3	2	3	34
Gene sub-lineages	19	20	6	8	7	9	3	4	76
Gene types	38	38	29	31	34	32	23	23	248
Gene types identified in species other than ducks	14	14	14	18	22	19	22	18	141

a Gene lineages, sub-lineages and types are defined as sharing ≥90%, ≥95% and ≥99% nucleotide identity, respectively.

**Table 2:** Inter-continental reassortants identified in the 109 Atlantic flyway duck AIVs.

Virus	PB2	PB1	PA	HA	NP	NA	M	NS
A/mallard/Maryland/802/2007(H5N1)	C-2.7	F-7.1	H-1.13	5C-1.2	F-1.1^a^	1E- 2.1	E-1.5	1D-1.7
A/hooded merganser/New Brunswick/03750/2009(H13N6)	J-1.1^b^	F-8.1^c^	E-6.1^c^	13A-1.1^c^	D-1.1^c^	6A-1.2	J-1.1^b^	1C-1.1^c^
A/mallard/Quebec/02916-1/2009(H16N3)	J-1.2^b^	F-8.1^c^	E-6.2^c^	16D-1.1^b^	D-1.1^c^	3D-1.1^b^	J-1.1^b^	1C-1.1^c^
A/American black duck/Newfoundland/MW733/2010(H6N6)	C-2.3	F-3.1	H-1.4	6B-1.1^a^	H-1.3	6A-3.2	E-1.1	1D-1.4
A/American black duck/Newfoundland/PR007/2010(H6N6)	C-2.1	F-3.1	H-1.5	6B-1.1^a^	H-1.1	6A-2.1	E-1.6	1D-1.5
A/duck/Newfoundland/MW721/2010(H6N8)	C-2.1	F-3.1	H-1.4	6B-1.1^a^	H-1.3	8A-1.3	E-1.1	1D-1.4

a Eurasian avian origin.

b Eurasian gull origin.

c North American gull origin.

### Genetic features of each gene segment of the Atlantic flyway duck viruses

**Figure 1:**
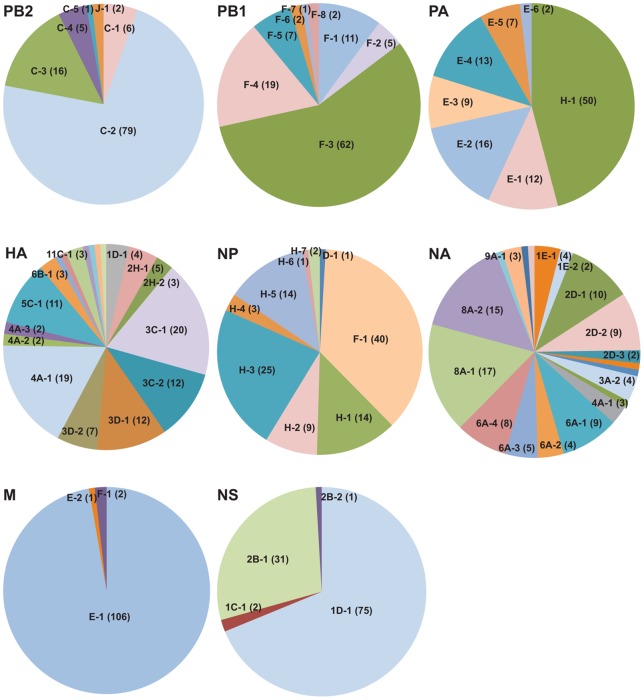
Genetic structure of the gene lineages and sub-lineages of the Atlantic flyway duck AIVs. The numbers of genes in each detected sub-lineage for each of the segments of the 109 Atlantic flyway duck AIVs are shown in the pie charts. AIV genes with ≥95% nucleotide identity by pairwise distance analysis were assigned in a sub-lineage. For the HA genes, sub-lineages 7F-1, 7F-2, 11C-2, 12A-1, 13A-1 and 16D-1 were each detected once and are not labelled. Similarly for the NA genes, sub-lineages 2G-1, 3A-1, 3D-1, 8A-3, 9A-2 and 9A-3 were each detected once and are not labelled.

All 8 segments of the 109 Atlantic flyway viruses had diverse gene types, but their genetic structures had pronounced differences (Table 1; Figure 1). Predominant single lineages and sub-lineages were found in the PB2, PB1 and M genes. The NP segments were represented by a single dominant lineage, but a number of different sub-lineages within this lineage. The PA segments were from 2 main lineages, each with very different sub-lineage structures. The NS segments were from 2 main lineages, with a major sub-lineage for each. The HA and NA segments had the highest diversity, with multiple lineages and sub-lineages identified. Investigation of the phylogenetic and epidemiological features of the 109 Atlantic flyway duck AIVs revealed the following information for each gene segment.

#### PB2

**Figure 2:**
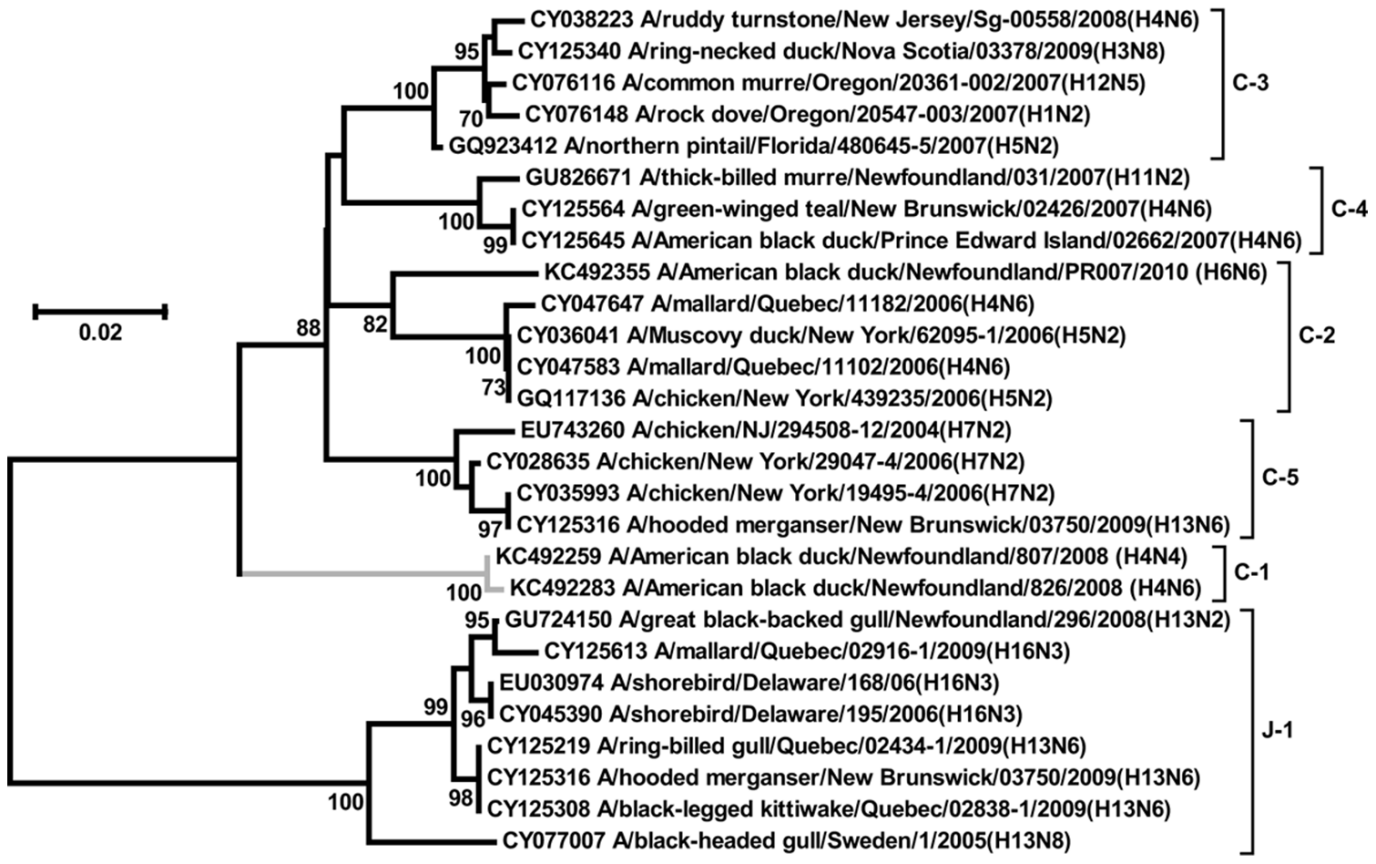
Detection of Atlantic flyway duck AIV PB2 gene sub-lineages in non-duck host species. The phylogenetic tree contains representative sequences from the Atlantic flyway duck AIVs for each of the 6 PB2 sub-lineages, with sub-lineages indicated on the right. The 5 sub-lineages (C-2 through C-5 and J-1) that contained genes detected from non-duck host species are displayed with black branches. The sub-lineage C1, which did not contain genes from non-duck host species, is displayed with grey branches. The neighbour-joining tree was constructed with MEGA 5 and support values based on 1000 bootstrap replicates are shown as percentages where ≥70%. The scale bar indicates nucleotide substitutions per site.

The PB2 sequences belonged to 2 gene lineages, J and C, with a single J sub-lineage and 5 C sub-lineages (Figures 1 and S1; Table S2). The 2 genes in the J-1 lineage were of Eurasian gull origin, and this lineage has also been detected in shorebirds and gulls of the Atlantic flyway (Figure 2). The remaining 106 genes were classified in C lineages, with the predominant C-2 sub-lineage detected in 79 viruses with 17 different gene types. In general, the C lineage of the PB2 segment is abundant in birds (waterfowl, gulls and poultry) of North America, but it has also been found in Eurasian ducks and seabirds, and in the 2009 H1N1 pandemic virus (Flugenome database).

#### PB1

All of the PB1 sequences belonged to the F lineage, with 8 sub-lineages (Figure 1). The F-3 sub-lineage was predominant and comprised of 12 gene types from 62 viruses (Figure S1; Table S2). In general, the F lineage PB1 gene is common in birds of North America, and has spilled over to swine (1999–2002) and humans (2003–2004). Several F-lineage genes have also been detected in Eurasian ducks and seabirds (Flugenome database).

#### PA

The PA sequences fell within 2 lineages (E and H; Figure 1) with 34 different gene types (Table S2). Browsing through the Flugenome database, the H lineage is common in birds of North America, but several genes of this lineage have been reported in Eurasia. The PA gene of the 2009 H1N1 pandemic virus is also assigned to the H lineage. E-lineage PA genes are common in birds from both North America and Eurasia.

#### HA

There were 11 subtypes found in the HA genes, with 4 H1, 8 H2, 51 H3, 23 H4, 11 H5, 3 H6, 2 H7, 4 H11, 1 H12, 1 H13 and 1 H16 categorized into 12 gene lineages and 19 sub-lineages (Figure 1 and Figure S1; Table S2). The 4 H1 genes belonged to the 1D lineage, which is abundant in waterfowl in North America and distinct from the 1A lineage often found in poultry in North America and found in the 2009 H1N1 pandemic strain (Flugenome database). The H2 genes all belonged to the 2H lineage, which is distinct from the 2D lineage that invaded North America from Eurasia [23], [27]. All of the H3 and H4 genes of the Atlantic flyway duck AIVs were from lineages typically identified in waterfowl in North America (Figure S1). The 11 H5 genes all belonged to the 5C lineage, which is abundant in waterfowl and poultry of North America and distinct from the H5 genes of the highly pathogenic H5N1 viruses in Eurasia, which belong to the 5J lineage (Flugenome database). The 3 H6 genes belonged to the 6B lineage of Eurasian-avian origin, which is known to have become the predominant H6 lineage in North America in the last decade [28]. The 2 H7 genes were classified in the 7F lineage (Figure S2; Table S2), which is abundant in waterfowl and poultry of North America with several genes detected from European poultry, while most H7 genes from waterfowl and poultry in Eurasia belonged to 7A lineage (Flugenome database). The 4 H11 genes belong to the 11C lineage (Table S2), which is abundant in waterfowl of North America and occasionally detected from shorebirds and poultry (Flugenome database). Several 11C-lineage genes were also detected from storks and ducks in Eurasia, although most H11 genes detected in Eurasia belonged to 11A lineage (Flugenome database). The H12 gene in this study belongs to the 12A lineage (Table S2) and the most similar sequences in the NCBI Influenza database (∼98% nucleotide identity) were from shorebirds of New Jersey and Delaware in 2008 (Figure S2). HA genes in the 12A lineage were detected from waterfowl, shorebirds, gulls and murres in North America, while those from Eurasia are mainly in the 12B lineage (Flugenome database). The one H13 gene belonged to the 13A lineage and may have recently been transmitted from gulls to ducks because the same gene type was detected in gulls from Quebec in the same year (2009) and these genes are closely related to an H13 gene found in a Newfoundland gull in 2008 (Figure S2). The one H16 gene was of Eurasian-gull origin and related to genes detected in shorebirds and gulls in Delaware and gulls in Europe (Figure S2). There are 3 lineages of H16 genes in the Flugenome database (16A, 16B and 16C) but the H16 gene from the Atlantic flyway duck in this study was divergent from these (≤90% nucleotide identity) and we therefore assigned it as the 16D lineage (Figure S2).

#### NP

The 109 NP sequences belonged to 3 lineages (F, H and D) but almost all (106/109) were from the H lineage (Figure 1 and Figure S1; Table S2), which is the predominant lineage in birds of North America (Flugenome database). NP genes of this lineage have also been detected in humans and swine, and 8 Eurasian H-lineage genes from Asia were found in the Flugenome database. The 2 NP genes in the D lineage were of American-gull origin (Table S2). Several D-lineage NP genes were also detected from gulls of Eurasia (Flugenome database). The single NP gene we found in the F lineage was of Eurasian-avian origin, and this lineage is common in Eurasian waterfowl and poultry (Flugenome database). Phylogenetic analyses indicated that these genes might have been brought to North America by ducks in the Pacific flyway (Figure S1), which is also supported by the epidemiological data because 49 of the 53 F-lineage NP genes in the Flugenome database from North America are from Pacific flyway waterfowl. The NP genes from some human AIV infections (H5N1 and H9N2 subtypes) in Asia also belong to the F lineage.

#### NA

The 108 NA sequences available from the 109 Atlantic flyway viruses fell into 7 NA subtypes with 9 gene lineages and 20 sub-lineages (Figure 1 and Figure S1; Table S2). The 6 N1 genes belonged to 2 sub-lineages of the 1E lineage, which is common in waterfowl and shorebirds of North America (Flugenome database). Most of the N2 genes (26/27) belonged to the 2D lineage, which is mainly found in waterfowl of North America and occasionally in other bird species such as chickens, seabirds and gulls. One N2 gene belonged to the 2G lineage, which is common in birds of North America but which has also been found in human AIVs in North America (Flugenome database). The 6 N3 genes were from 2 lineages, 3A and 3D. The 3A lineage is common in birds of North America and sometimes also detected in swine and human AIV viruses. The gene from the 3D lineage was of Eurasian-gull origin (Table S2). The 4 N4 genes belonged to the 4A lineage, which is mainly found in waterfowl of North America. The 26 N6 genes are from 4 sub-lineages of the 6A lineage, which is abundant in waterfowl and shorebirds in North America and occasionally detected in birds in Eurasia (Flugenome database). The gene type 6A-1.2 was also detected in gulls from Quebec in 2009 (Figure S2). Considering the predominance of the 6A-lineage NA genes in ducks of North America, this likely represents a recent cross-species transmission of this gene from ducks to gulls. There were 34 N8 genes and all belonged to the 8A lineage (Figure S2). In general, the 8A lineage is abundant in waterfowl, shorebirds and gulls of North America but also occasionally detected in aquatic birds in Asia (Flugenome database). The 5 N9 genes belonged to 3 sub-lineages of the 9A lineage, which has mainly been detected in waterfowl, shorebirds and gulls of North America but there were also 33 NA genes of 9A lineage from Eurasia in the Flugenome database.

#### M

The M sequences belonged to the E and F lineages. The E-1 sub-lineage was predominant and included 106 of the viruses (Figure S1; Table S2). The E-lineage is common in birds of North America with several genes detected in human and swine cases of AIV infection. Thirteen E-lineage genes from birds and swine in Asia were also found in the Flugenome database. The M gene of the F-1 sub-lineage was of Eurasian gull origin, which has previously been detected in American gulls and shorebirds, and has now also been detected in Atlantic flyway ducks (Figure S2).

#### NS

Both alleles 1 and 2 (allele A and B by conventional nomenclature) were detected in the Atlantic flyway duck AIVs. There were 77 genes from lineage 1, with 75 genes from lineage 1D and 2 from lineage 1C (Figure 1 and Figure S1; Table S2). The 1D lineage is abundant in waterfowl and shorebirds of North America, and has also been detected in poultry, seabirds, gulls, swine and humans in North America (Flugenome database). The 2 genes from the 1C lineage were of American gull origin (Figure S1). The remaining 32 NS genes belonged to lineage 2B, which is common in waterfowl and poultry of North America and also has been detected in other hosts (Flugenome database).

### Spatial and temporal detection of AIV genes and genomes within Atlantic flyway ducks

The 248 Atlantic flyway gene types were sorted according to year and location of detection (Figure S3 and Figure S4). Circulation of the genes in the Atlantic flyway through space and time was common, with 81 of the 248 gene types (32%) detected in more than one year or at different locations over 2006–2011, and 22 gene types (9%) were maintained in Atlantic flyway ducks for 3 to 6 years during 2006–2011 (Table S5). For example, the NA gene type 2D-1.1 was found in several locations of the Atlantic flyway over the 6 years and the HA gene type 3D-1.1 was perpetuated in Newfoundland over 4 years (2007–2011) (Table S5). Different locations possessed both unique and shared gene types. For example, amongst the 78 total gene types from Newfoundland, 18 were also detected elsewhere in the Atlantic flyway and 60 were restricted to Newfoundland. This was different than what was observed for the viruses from Quebec, where 30 of the 51 identified gene types were also detected at other locations in the Atlantic flyway during 2006–2011 and 21 genes were exclusively found in that province. However, the limited number of AIV reference sequences in Atlantic flyway ducks could cause under-estimation of virus transmission within Atlantic flyway, because searching of the NCBI database showed that 69 of the 78 gene types from Newfoundland have also been found in other bird flyways (Table S6 and Table S7).

At the genomic level, 12 homologous genomes were detected more than once (Table S2). Distinct from the long perpetuation of individual gene segments, most (9/12) of the homologous genomes were detected during the same year and from the same location. The exceptions to this were H1N1 viruses detected in consecutive years (both 2009 and 2010 in Newfoundland), H3N8 viruses detected in multiple locations (2009 in Nova Scotia, New Brunswick and Prince Edward Island), and H4N6 viruses detected in multiple locations (2007 in New Brunswick and Prince Edward Island).

### Spatial and temporal detection of the Atlantic flyway duck AIV genes in different North American flyways

Highly similar reference gene sequences (≥99% identical) from North America were identified for the 248 Atlantic flyway duck AIV gene types by BLAST, which totaled >9,000 sequences. These were categorized by year (Table S6) and migratory flyway of detection (Table S7). Extensive exchanges of the 248 Atlantic flyway AIV genes through space and time were detected, with 188 gene types (75.8%) identified in other flyways, while 42 of the gene types (16.9%) were identified only in the Atlantic flyway before 2006. The remaining 18 gene types (7.3%), of which 11 were HA genes, were only detected in Atlantic flyway ducks from 2006 to 2011 (Table S5).

**Figure 3:**
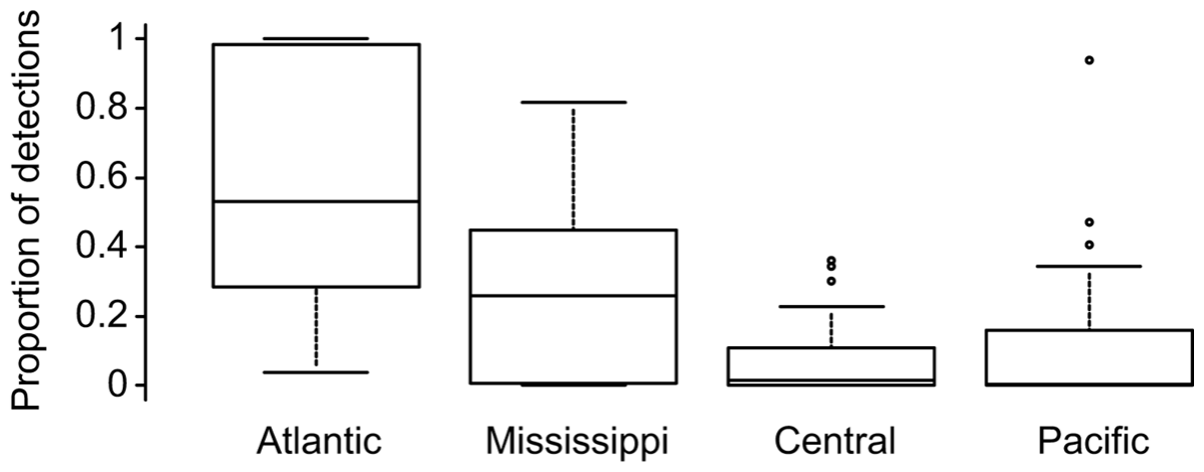
Spatial distribution of detections of the Atlantic flyway duck AIV sub-lineages in North American flyways. The box plots show the proportions of detections in each of the flyways for the gene sub-lineages that were identified in the 2006–2011 Atlantic flyway duck AIVs. The solid line in the box is the median. The top and bottom of the box are the first and third quartiles, respectively. The upper and lower horizontal lines represent 1.5X the difference between the first and third quartiles and any values falling outside of those are plotted as points. The detailed results for the each segment are provided in Figure S5.

Despite the frequent transmission of viruses among different bird flyways, the overall detection of genes highly similar to those from the 2006–2011 Atlantic flyway duck AIVs was most frequent in other viruses from the Atlantic flyway and decreased moving west across the different flyways (Figure 3). The majority of the 248 gene types displayed spatial distribution biases, as reflected by the larger numbers of detections of specific genes in one or two neighbouring flyways compared to the other flyways (Figure S5). For example, the HA genes of 3D-1, 4A-1 and 7F-2 sub-lineages were abundant in the Atlantic flyway, whereas the 4A-3 lineage was more abundant in the Mississippi and Central flyways (Figure S5). In contrast, the 1D-1 HA lineage was more evenly detected in the four flyways. Similarly for the NP genes, the H-4 and H-7 sub-lineages were more abundant in the Atlantic flyway, the H-1 and H-2 lineages were more widely detected, and the F-1 sub-lineage was mainly detected in the Pacific flyway (Figure S5). The NP F-1 sub-lineage was the most Pacific-biased of any sub-lineage (top point on Pacific flyway plot in Figure 3) and this may indicate it was recently transmitted from the Pacific flyway into eastern North America.

### Perpetuation of the AIV genes through years

**Figure 4:**
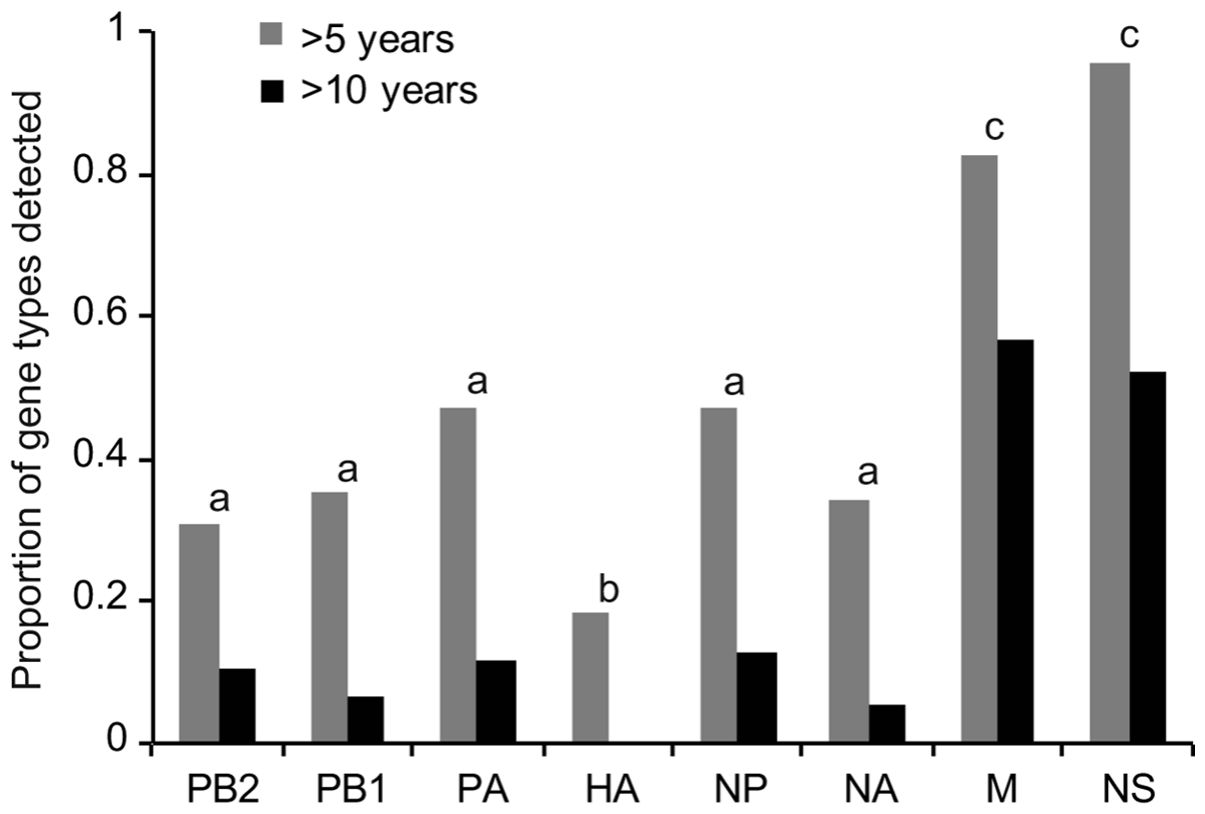
Perpetuation of Atlantic flyway duck AIV gene types across time. The proportions of gene types identified in the Atlantic flyway viruses that were detected for >5 and >10 years in North America are shown for each segment. There were significant differences among the proportions for the HA, M and NS segments relative to the other segments for >5 years (χ^2^ = 53.1, df = 7, P<0.0001) and >10 years (χ^2^ = 66.5, df = 7, P<0.0001). Statistically different proportions for the segments are indicated with different letters (a, b and c).

The perpetuation of the AIV segments was evident after comparing them with all highly similar genes available in the NCBI database. This showed that 175 of the 248 gene types (70.6%) have persisted for >3 years, and 112 of the 248 gene types (45.1%) have been maintained for >5 years, while 40 of the 248 gene types (16.1%) have circulated for >10 years (Table S6). Eleven of the gene types could be traced to more than 20 years ago, with the oldest detection from 1978 (Table S6). When the 8 AIV gene segments were compared for their maintenance across the years (Figure 4), the M and NS segments had the highest >5-year perpetuation detections, at 82.6% and 95.7%, respectively, whereas the HA segment had the lowest proportion of maintenance for >5 years (18.4%). Similarly, the M and NS segments had the highest >10-year perpetuation detections, at 56.5% and 52.1%, respectively, whereas no HA segment was maintained for >10 years (Figure 4). These differences were significant for both the >5 year (χ^2^ = 53.1, df = 7, P<0.0001) and >10 year (χ^2^ = 66.5, df = 7, P<0.0001) data.

### Identification of Atlantic flyway duck AIV genes in other hosts

Of the 248 AIV gene types identified in the 109 Atlantic flyway duck viruses, 142 (57.3%) were also detected in hosts other than ducks (Table S8). These included shorebirds, poultry, gulls, murres, other waterfowl, and swine (Table S9). The detection frequency of the Atlantic flyway duck AIV genes in different host groups varied, with 82 of the gene types found in shorebirds, 41 in geese, 39 in turkeys, 37 in chickens, 19 in gulls, 13 in swans, 9 in seabirds and 9 in swine (Table S9).

## Discussion

Current attempts to characterize AIV genetic structure and fully understand virus ecology are limited by the under-representation of sequence information from viruses across spatial, temporal, and host species scales, especially in the American portion of the Central flyway and the Canadian portion of the Atlantic flyway [7]. There is clearly an enormous virus reservoir maintained in both bird hosts [2], [3] and the natural environment [29]–[31] and we have discovered only a fraction of this diversity. To better resolve the dynamics of AIV transmission and distribution, it is critical to examine available sequences in the context of broader time scales, host species and geographic locations. In this study, 109 Atlantic flyway duck AIV genomes from 2006–2011 were analyzed for their genetic structure to provide insight into AIV evolution and ecology on the east coast of North America. Extensive diversity was detected in the AIV genes and genomes from the Atlantic flyway ducks in this study, similar to reports performed in waterfowl at other locations of North America (e.g. [8], [10], [32]). The analysis of the AIV genetic structure identified limited numbers of predominant gene lineages and sub-lineages for the internal protein genes, distinct from what was found for the surface protein genes. Restricted inter-continental AIV exchange was found in the Atlantic flyway ducks, in sharp contrast to the extensive intra-continental transmission among bird flyways and bird species.

The host sources of the AIVs analyzed in this study (Table S2) generally reflected duck distribution patterns in North America. However, the spatial distribution of duck species does not lead to the geographic distributions of AIV genes as shown by the identification of homologous genes in different duck species (Figure S1 and Table S2). This agrees with previous work, which also found no distinct AIV phylogenetic separation in dabbling duck species [6], [10]. We detected a high proportion (>50%; Table S8) of the Atlantic flyway duck AIV gene types in bird groups other than ducks, reflecting frequent interspecies transmission of AIVs in birds. The duck gene types were detected more often in shorebirds than other wild bird groups such as gulls and seabirds. This variation in detection frequency of the Atlantic flyway duck AIV genes in different bird groups could be caused by multiple factors, including differences in virus sequence availability, bird habitat use and migratory behaviors, bird population structure, AIV subtype prevalence, host AIV infection history and cellular receptor properties. The Atlantic flyway duck AIV gene types were also detected in domestic poultry (Table S9), reflecting the infection of domestic birds by wild bird viruses. Similarly, some of the gene types were identified in swine viruses (Table S9). This movement of AIV genes amongst wild birds and domestic animals is an important aspect of influenza dynamics as related to the evolution of new strains and the potential for negative impacts on human society in terms of agriculture and health. Continual surveillance of the wild bird AIV reservoir is especially valuable to monitor virus transmission to domestic animals and/or the human population.

Many AIV genes in our study were detected in more than one North American bird flyway, but the detections were biased by geographic locations (Figure 3). Most AIV gene sub-lineages were detected more often in in one or several neighbouring flyways relative to other flyways (Figure S5). It should be noted that there are differences in the numbers of duck virus sequences available from the different flyways, which could bias these results. However, we do not believe this pattern is caused by an over-representation of sequences from the Atlantic flyway because recent work has increased the sequences available for multiple regions of North America, particularly in the Pacific flyway. Furthermore, differential gene distributions by flyway were also found in a previous study [7]. Gene flow among flyways is presumably mediated by interactions of long-distance migrants on wintering grounds and some east-west bird movements [7], [8]. At the genomic level, highly similar viruses were mostly detected in adjacent locations and within short time spans (Table S2), reflecting frequent viral reassortment [10], [33]. The between-year detection of the 4 H1N1 viruses may reflect the maintenance of AIVs in the abiotic reservoir [16]. Among the 8 AIV gene segments, the HA gene displayed the lowest maintenance time with less than 20% of the gene types maintained for >5 years and none retained >10 years (Figure 4), which is not surprising considering that the HA protein is targeted by host immune responses. Along with the lowest lineage diversity in the Atlantic flyway ducks (Figure 1), the M and NS segments showed the most prolonged maintenance, with >80% of the identified gene types maintained for >5 years and >50% maintained for >10 years (Figure 4). This likely resulted from differences in the selective pressures on the different segments, and it was previously found that the M and NS (allele A) segments showed the lowest nucleotide substitution rates [34]. Prolonged prevalence of the same gene types may result in part from maintenance of viruses in the environment [30], [31], [35]–[38] because the high mutation rate for these RNA viruses and the constant immune pressure from the hosts make it unlikely for the same AIV genes to be maintained solely in birds across many years [39], [40].

The detection frequency of inter-continental AIV reassortants is higher at the continental margins than inland [11], [41]. Compared to the amount of inter-continental reassortment detected in AIVs from Northern Pintails in Alaska [11], [14], fewer inter-continental AIV genes (2%) were identified in Atlantic flyway ducks in our analysis. Limited inter-continental reassortants were also detected in Atlantic flyway shorebirds [12]. This could be due to the larger distance between eastern North America and Europe compared to that between Alaska and Russia, leading to more restricted movements of birds between the regions, especially ducks. Therefore, our study suggests there is a lower likelihood of AIV introduction from Eurasia to North America through ducks of the Atlantic flyway, compared to those of the Pacific flyway, which has implications for the potential transmission of highly pathogenic AIVs, such as H5N1, to North America through Atlantic Canada. There is currently no evidence that Atlantic flyway ducks move across the Atlantic Ocean and therefore the Eurasian sequences detected may result from the interaction of these ducks with other hosts such as gulls, which do move across the Atlantic [42] and which contain inter-continental reassortant viruses with high frequency [43]–[45]. Continued AIV surveillance in multiple bird groups in different regions will further increase our understanding of AIV ecology in North America and virus movement between regions.

## Supporting Information

Figure S1 **Phylogenetic analysis of the 8 segments of the 2006–2011 Atlantic flyway duck AIVs.** Phylogenetic trees are shown for each segment of the 109 Atlantic flyway duck AIVs (indicated with red circles), with separate trees for the different HA and NA subtypes. The sub-lineages (≥95% nucleotide identity within a sub-lineage) of the AIV genes from Atlantic flyway are labelled on the right. The neighbour-joining trees were constructed with MEGA5 and support values based on 1000 bootstrap replicates are shown as percentages where ≥70%. Use of maximum likelihood produced the same lineage topologies. The scale bars indicate nucleotide substitutions per site. The full identification information for the Atlantic flyway viruses is provided in Table S2. Abbreviations: ABDU, American Black Duck; RNDU, Ring-necked Duck; GWTE, Green-winged Teal; BWTE, Blue-winged Teal; AMWI, American Widgeon; NOPI, Northern Pintail; NL, Newfoundland; QC, Quebec; NS, Nova Scotia; NB, New Brunswick; NY, New York; PEI, Prince Edward Island; NS, Nova Scotia; PA, Pennsylvania; DE, Delaware; MD, Maryland; FL, Florida; AK, Alaska; CA, California; MN, Minnesota; MS, Missouri; SK, Saskatchewan.
(PDF)

Figure S2 **Identification of Atlantic flyway duck AIV genes in other bird hosts.** Phylogenetic trees were constructed to highlight the distribution of the genes found in the 109 Atlantic flyway duck AIVs in non-duck host species. The neighbour-joining trees were constructed with MEGA5 and support values based on 1000 bootstrap replicates are shown as percentages where ≥70%. The sub-lineages are labelled on the right of the phylogenetic trees. Black branch lines indicate detection of the sub-lineage in non-duck species, whereas grey branch lines indicate the detection of the sub-lineage only in ducks.
(PDF)

Figure S3 **Gene type categorization of the Atlantic flyway duck AIVs by year and location.** Gene typing was done as described in the Materials and Methods section such that sequences with nucleotide identity of ≥99% are considered as homologous genes or the same gene type. Sequences from different locations within the Atlantic bird flyway are shown in different colors, as indicated in the legend. The locations are abbreviated as follows: NL, Newfoundland; QC, Quebec; MD, Maryland; NB, New Brunswick; PEI, Prince Edward Island; NY, New York; PA, Pennsylvania; DE, Delaware; ON, Ontario; FL, Florida; NS, Nova Scotia.
(PDF)

Figure S4 **Distribution of the AIV gene types from Atlantic flyway ducks by location.** Gene typing was done as described in the Materials and Methods section such that sequences with nucleotide identity of ≥99% are considered as homologous genes or the same gene type. Sequences from different locations within the Atlantic bird flyway are shown in different colors, as indicated in the legend. The locations are abbreviated as follows: NL, Newfoundland; QC, Quebec; MD, Maryland; NB, New Brunswick; PEI, Prince Edward Island; NY, New York; PA, Pennsylvania; DE, Delaware; ON, Ontario; FL, Florida; NS, Nova Scotia. The 18 gene types detected only in Newfoundland are labelled with asterisk (*).
(PDF)

Figure S5 **Spatial distribution of detections of the Atlantic Flyway duck AIV gene sub-lineages in North American flyways.** The numbers of viruses identified in each flyway are shown for the indicated gene sub-lineages that were identified in the 2006-2011 Atlantic flyway duck viruses. Only sub-lineages with ≥10 genes detected in at least one flyway are shown. Flyways are shown in different shading: Atlantic flyway (AF), black; Mississippi flyway (MF), dark grey; Central flyway (CF), light grey; Pacific flyway (PF), white.
(PDF)

Table S1 **The 25 duck AIVs from Newfoundland, Canada, sequenced in this study.**
(PDF)

Table S2 **Genetic analysis of the 109 Atlantic flyway duck AIVs (from 2006 to 2011).**
(PDF)

Table S3 **AIV gene typing summary for Atlantic flyway locations with 20 or more viruses.**
(PDF)

Table S4 **AIV genotyping for Atlantic flyway locations with 20 or more viruses.**
(PDF)

Table S5 **Repeated gene type detections across space and time in ducks of the Atlantic flyway over 2006–2011.**
(PDF)

Table S6 **Detection of the 248 Atlantic flyway AIV gene types through years in North American flyways.**
(PDF)

Table S7 **Detection of the 248 identified Atlantic flyway AIV gene types in the North American flyways.**
(PDF)

Table S8 **Summary of detections of the Atlantic Flyway duck AIV genes in non-duck hosts.**
(PDF)

Table S9 **Identification of Atlantic Flyway duck AIV gene types in hosts other than ducks.**
(PDF)
