# Supplementary material for: Genetic Structure of Avian Influenza Viruses from Ducks of the Atlantic Flyway of North America
Source: PLoS One. 2014 Jan 30;9(1):e86999. doi: 10.1371/journal.pone.0086999 (PMC3907406; doi:10.1371/journal.pone.0086999)
Supplement: Figure S1 — Phylogenetic analysis of the 8 segments of the 2006–2011 Atlantic flyway duck AIVs. Phylogenetic trees are shown for each segment of the 109 Atlantic flyway duck AIVs (indicated with red circles), with separate trees for the different HA and NA subtypes. The sub-lineages (≥95% nucleotide identity within a sub-lineage) of the AIV genes from Atlantic flyway are labelled on the right. The neighbour-joining trees were constructed with MEGA5 and support values based on 1000 bootstrap replicates are shown as percentages where ≥70%. Use of maximum likelihood produced the same lineage topologies. The scale bars indicate nucleotide substitutions per site. The full identification information for the Atlantic flyway viruses is provided in Table S2. Abbreviations: ABDU, American Black Duck; RNDU, Ring-necked Duck; GWTE, Green-winged Teal; BWTE, Blue-winged Teal; AMWI, American Widgeon; NOPI, Northern Pintail; NL, Newfoundland; QC, Quebec; NS, Nova Scotia; NB, New Brunswick; NY, New York; PEI, Prince Edward Island; NS, Nova Scotia; PA, Pennsylvania; DE, Delaware; MD, Maryland; FL, Florida; AK, Alaska; CA, California; MN, Minnesota; MS, Missouri; SK, Saskatchewan. (PDF) [file pone.0086999.s001.pdf]

Figure S1. Phylogenetic analysis of the 8 segments of the 2006-2011 Atlantic flyway duck AIVs. Phylogenetic trees are shown for each segment of the 109 Atlantic flyway duck AIVs (indicated with red circles), with separate trees for the different HA and NA subtypes. The sub-lineages (≥95% nucleotide identity within a sub-lineage) of the AIV genes from the Atlantic flyway are labelled on the right. The neighbour-joining trees were constructed with MEGA5 and support values based on 1000 bootstrap replicates are shown as percentages where ≥70%. The scale bars indicate nucleotide substitutions per site. The full identification information for the Atlantic flyway viruses is provided in Table S2. Abbreviations: ABDU, American Black Duck; RNDU, Ring-necked Duck; GWTE, Green-winged Teal; BWTE, Blue-winged Teal; AMWI, American Widgeon; NOPI, Northern Pintail; NL, Newfoundland; QC, Quebec; NS, Nova Scotia; NB, New Brunswick; NY, New York; PEI, Prince Edward Island; NS, Nova Scotia; PA, Pennsylvania; DE, Delaware; MD, Maryland; FL, Florida; AK, Alaska; CA, California; MN, Minnesota; MS, Missouri; SK, Saskatchewan.

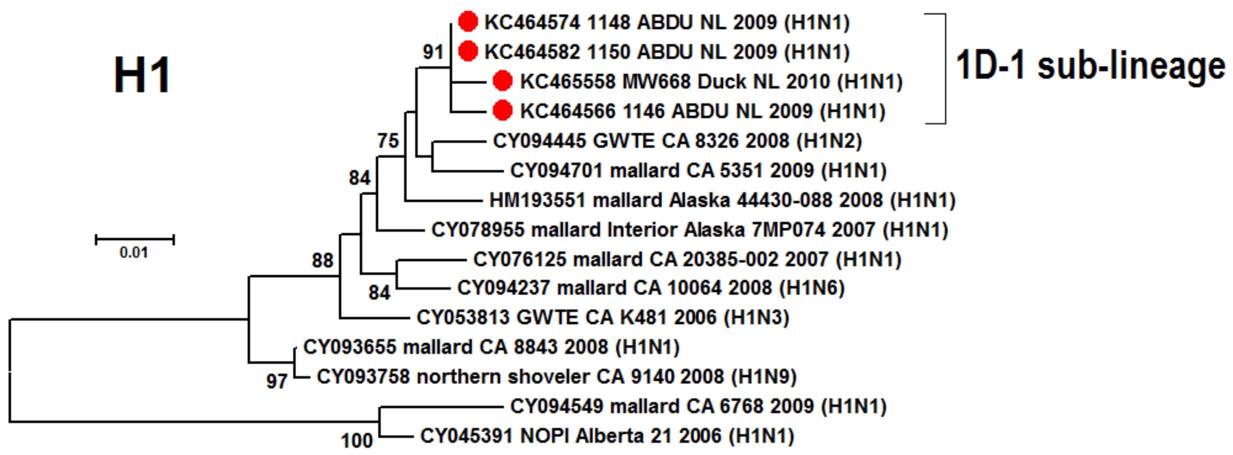
H1
1D-1 sub-lineage
KC464574 1148 ABDU NL 2009 (H1N1)
KC464582 1150 ABDU NL 2009 (H1N1)
KC465558 MW668 Duck NL 2010 (H1N1)
KC464566 1146 ABDU NL 2009 (H1N1)
CY094445 GWTE CA 8326 2008 (H1N2)
CY094701 mallard CA 5351 2009 (H1N1)
HM193551 mallard Alaska 44430-088 2008 (H1N1)
CY078955 mallard Interior Alaska 7MP074 2007 (H1N1)
CY076125 mallard CA 20385-002 2007 (H1N1)
CY094237 mallard CA 10064 2008 (H1N6)
CY053813 GWTE CA K481 2006 (H1N3)
CY093655 mallard CA 8843 2008 (H1N1)
CY093758 northern shoveler CA 9140 2008 (H1N9)
CY094549 mallard CA 6768 2009 (H1N1)
CY045391 NOPI Alberta 21 2006 (H1N1)
91
75
84
88
84
97
100
0.01

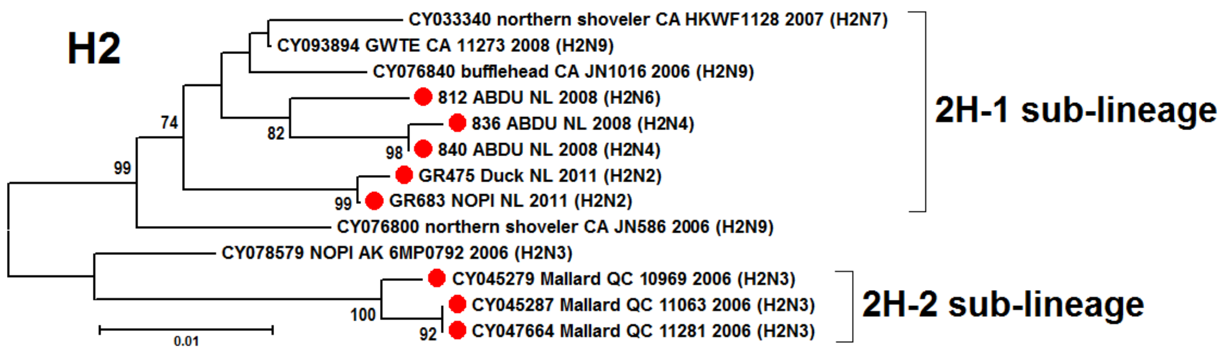
H2
2H-1 sub-lineage
2H-2 sub-lineage
CY033340 northern shoveler CA HKWF1128 2007 (H2N7)
CY093894 GWTE CA 11273 2008 (H2N9)
CY076840 bufflehead CA JN1016 2006 (H2N9)
812 ABDU NL 2008 (H2N6)
836 ABDU NL 2008 (H2N4)
840 ABDU NL 2008 (H2N4)
GR475 Duck NL 2011 (H2N2)
GR683 NOPI NL 2011 (H2N2)
CY076800 northern shoveler CA JN586 2006 (H2N9)
CY078579 NOPI AK 6MP0792 2006 (H2N3)
CY045279 Mallard QC 10969 2006 (H2N3)
CY045287 Mallard QC 11063 2006 (H2N3)
CY047664 Mallard QC 11281 2006 (H2N3)
74
82
98
99
99
100
92
0.01

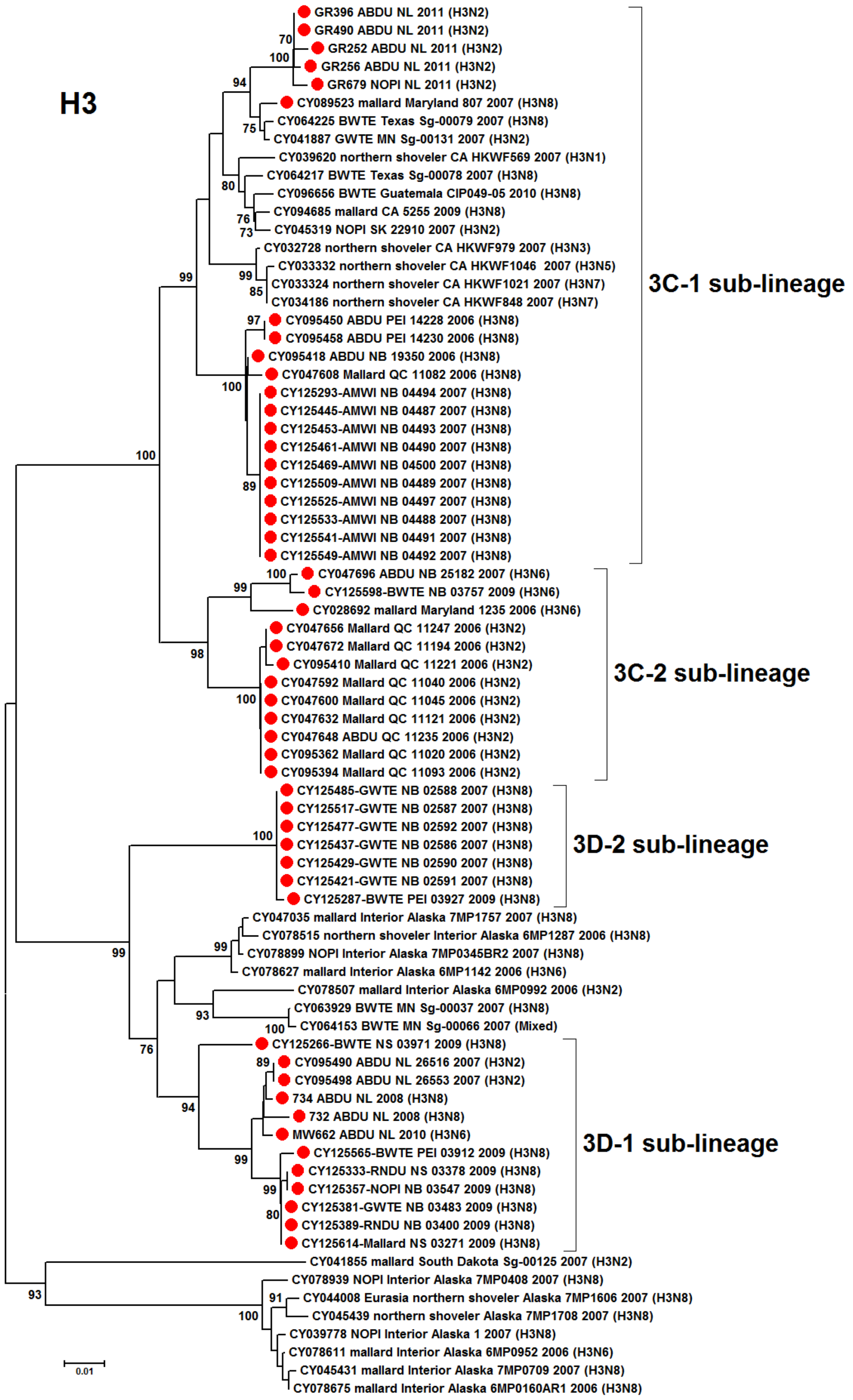
H3
GR396 ABDU NL 2011 (H3N2)
GR490 ABDU NL 2011 (H3N2)
GR252 ABDU NL 2011 (H3N2)
GR256 ABDU NL 2011 (H3N2)
GR679 NOPI NL 2011 (H3N2)
CY089523 mallard Maryland 807 2007 (H3N8)
CY064225 BWTE Texas Sg-00079 2007 (H3N8)
CY041887 GWTE MN Sg-00131 2007 (H3N2)
CY039620 northern shoveler CA HKWF569 2007 (H3N1)
CY064217 BWTE Texas Sg-00078 2007 (H3N8)
CY096656 BWTE Guatemala CIP049-05 2010 (H3N8)
CY094685 mallard CA 5255 2009 (H3N8)
CY045319 NOPI SK 22910 2007 (H3N2)
CY032728 northern shoveler CA HKWF979 2007 (H3N3)
CY033332 northern shoveler CA HKWF1046 2007 (H3N5)
CY033324 northern shoveler CA HKWF1021 2007 (H3N7)
CY034186 northern shoveler CA HKWF848 2007 (H3N7)
CY095450 ABDU PEI 14228 2006 (H3N8)
CY095458 ABDU PEI 14230 2006 (H3N8)
CY095418 ABDU NB 19350 2006 (H3N8)
CY047608 Mallard QC 11082 2006 (H3N8)
CY125293-AMWI NB 04494 2007 (H3N8)
CY125445-AMWI NB 04487 2007 (H3N8)
CY125453-AMWI NB 04493 2007 (H3N8)
CY125461-AMWI NB 04490 2007 (H3N8)
CY125469-AMWI NB 04500 2007 (H3N8)
CY125509-AMWI NB 04489 2007 (H3N8)
CY125525-AMWI NB 04497 2007 (H3N8)
CY125533-AMWI NB 04488 2007 (H3N8)
CY125541-AMWI NB 04491 2007 (H3N8)
CY125549-AMWI NB 04492 2007 (H3N8)
3C-1 sub-lineage
CY047696 ABDU NB 25182 2007 (H3N6)
CY125598-BWTE NB 03757 2009 (H3N6)
CY028692 mallard Maryland 1235 2006 (H3N6)
CY047656 Mallard QC 11247 2006 (H3N2)
CY047672 Mallard QC 11194 2006 (H3N2)
CY095410 Mallard QC 11221 2006 (H3N2)
CY047592 Mallard QC 11040 2006 (H3N2)
CY047600 Mallard QC 11045 2006 (H3N2)
CY047632 Mallard QC 11121 2006 (H3N2)
CY047648 ABDU QC 11235 2006 (H3N2)
CY095362 Mallard QC 11020 2006 (H3N2)
CY095394 Mallard QC 11093 2006 (H3N2)
3C-2 sub-lineage
CY125485-GWTE NB 02588 2007 (H3N8)
CY125517-GWTE NB 02587 2007 (H3N8)
CY125477-GWTE NB 02592 2007 (H3N8)
CY125437-GWTE NB 02586 2007 (H3N8)
CY125429-GWTE NB 02590 2007 (H3N8)
CY125421-GWTE NB 02591 2007 (H3N8)
CY125287-BWTE PEI 03927 2009 (H3N8)
3D-2 sub-lineage
CY047035 mallard Interior Alaska 7MP1757 2007 (H3N8)
CY078515 northern shoveler Interior Alaska 6MP1287 2006 (H3N8)
CY078899 NOPI Interior Alaska 7MP0345BR2 2007 (H3N8)
CY078627 mallard Interior Alaska 6MP1142 2006 (H3N6)
CY078507 mallard Interior Alaska 6MP0992 2006 (H3N2)
CY063929 BWTE MN Sg-00037 2007 (H3N8)
CY064153 BWTE MN Sg-00066 2007 (Mixed)
CY125266-BWTE NS 03971 2009 (H3N8)
CY095490 ABDU NL 26516 2007 (H3N2)
CY095498 ABDU NL 26553 2007 (H3N2)
734 ABDU NL 2008 (H3N8)
732 ABDU NL 2008 (H3N8)
MW662 ABDU NL 2010 (H3N6)
CY125565-BWTE PEI 03912 2009 (H3N8)
CY125333-RNDU NS 03378 2009 (H3N8)
CY125357-NOPI NB 03547 2009 (H3N8)
CY125381-GWTE NB 03483 2009 (H3N8)
CY125389-RNDU NB 03400 2009 (H3N8)
CY125614-Mallard NS 03271 2009 (H3N8)
3D-1 sub-lineage
CY041855 mallard South Dakota Sg-00125 2007 (H3N2)
CY078939 NOPI Interior Alaska 7MP0408 2007 (H3N8)
CY044008 Eurasia northern shoveler Alaska 7MP1606 2007 (H3N8)
CY045439 northern shoveler Alaska 7MP1708 2007 (H3N8)
CY039778 NOPI Interior Alaska 1 2007 (H3N8)
CY078611 mallard Interior Alaska 6MP0952 2006 (H3N6)
CY045431 mallard Interior Alaska 7MP0709 2007 (H3N8)
CY078675 mallard Interior Alaska 6MP0160AR1 2006 (H3N8)
0.01

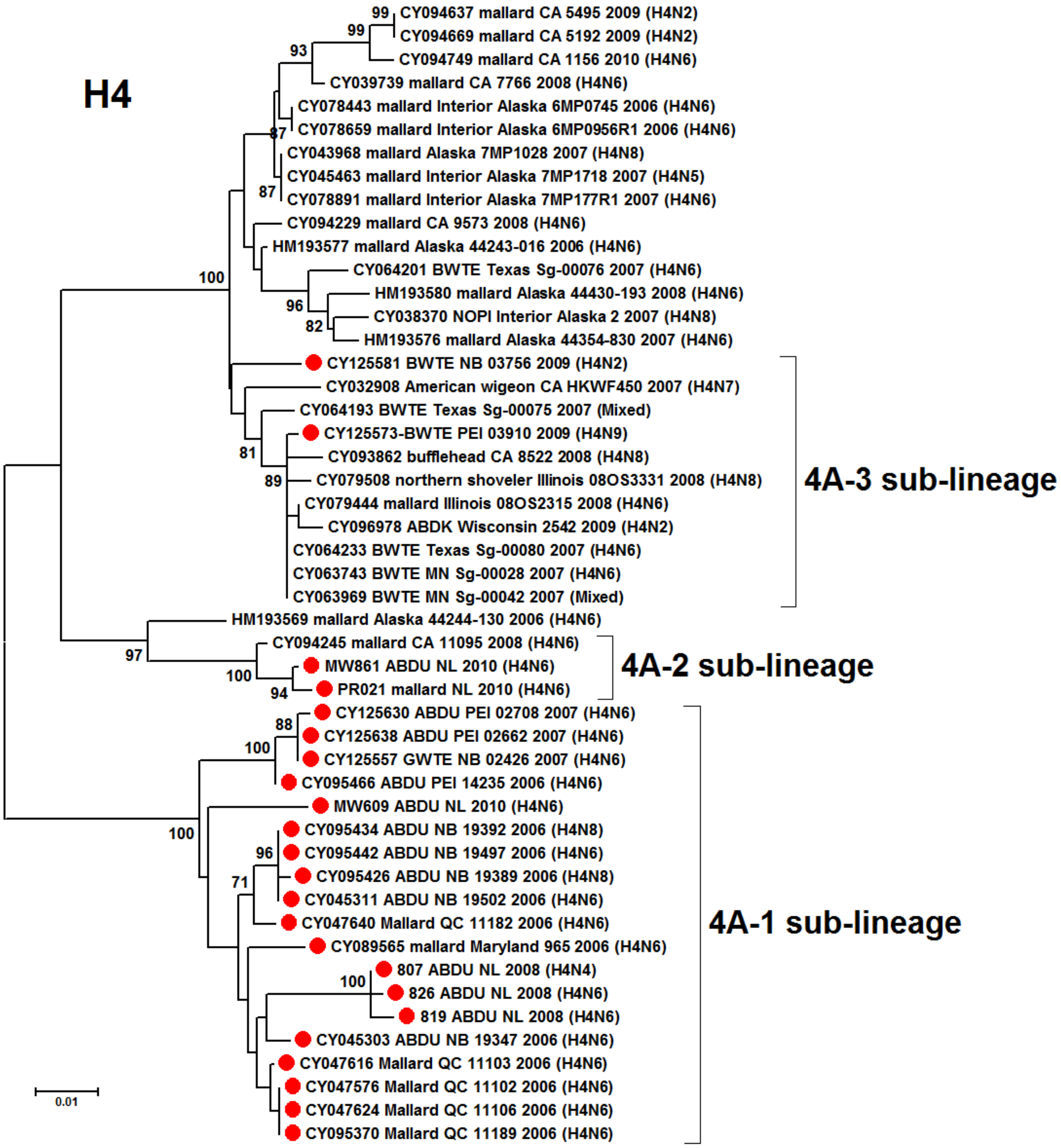
H4
99
99
93
87
87
100
96
82
81
89
97
100
94
88
100
100
96
71
100
0.01
CY094637 mallard CA 5495 2009 (H4N2)
CY094669 mallard CA 5192 2009 (H4N2)
CY094749 mallard CA 1156 2010 (H4N6)
CY039739 mallard CA 7766 2008 (H4N6)
CY078443 mallard Interior Alaska 6MP0745 2006 (H4N6)
CY078659 mallard Interior Alaska 6MP0956R1 2006 (H4N6)
CY043968 mallard Alaska 7MP1028 2007 (H4N8)
CY045463 mallard Interior Alaska 7MP1718 2007 (H4N5)
CY078891 mallard Interior Alaska 7MP177R1 2007 (H4N6)
CY094229 mallard CA 9573 2008 (H4N6)
HM193577 mallard Alaska 44243-016 2006 (H4N6)
CY064201 BWTE Texas Sg-00076 2007 (H4N6)
HM193580 mallard Alaska 44430-193 2008 (H4N6)
CY038370 NOPI Interior Alaska 2 2007 (H4N8)
HM193576 mallard Alaska 44354-830 2007 (H4N6)
CY125581 BWTE NB 03756 2009 (H4N2)
CY032908 American wigeon CA HKWF450 2007 (H4N7)
CY064193 BWTE Texas Sg-00075 2007 (Mixed)
CY125573-BWTE PEI 03910 2009 (H4N9)
CY093862 bufflehead CA 8522 2008 (H4N8)
CY079508 northern shoveler Illinois 08OS3331 2008 (H4N8)
CY079444 mallard Illinois 08OS2315 2008 (H4N6)
CY096978 ABDK Wisconsin 2542 2009 (H4N2)
CY064233 BWTE Texas Sg-00080 2007 (H4N6)
CY063743 BWTE MN Sg-00028 2007 (H4N6)
CY063969 BWTE MN Sg-00042 2007 (Mixed)
4A-3 sub-lineage
HM193569 mallard Alaska 44244-130 2006 (H4N6)
CY094245 mallard CA 11095 2008 (H4N6)
MW861 ABDU NL 2010 (H4N6)
PR021 mallard NL 2010 (H4N6)
4A-2 sub-lineage
CY125630 ABDU PEI 02708 2007 (H4N6)
CY125638 ABDU PEI 02662 2007 (H4N6)
CY125557 GWTE NB 02426 2007 (H4N6)
CY095466 ABDU PEI 14235 2006 (H4N6)
MW609 ABDU NL 2010 (H4N6)
CY095434 ABDU NB 19392 2006 (H4N8)
CY095442 ABDU NB 19497 2006 (H4N6)
CY095426 ABDU NB 19389 2006 (H4N8)
CY045311 ABDU NB 19502 2006 (H4N6)
CY047640 Mallard QC 11182 2006 (H4N6)
CY089565 mallard Maryland 965 2006 (H4N6)
807 ABDU NL 2008 (H4N4)
826 ABDU NL 2008 (H4N6)
819 ABDU NL 2008 (H4N6)
CY045303 ABDU NB 19347 2006 (H4N6)
CY047616 Mallard QC 11103 2006 (H4N6)
CY047576 Mallard QC 11102 2006 (H4N6)
CY047624 Mallard QC 11106 2006 (H4N6)
CY095370 Mallard QC 11189 2006 (H4N6)
4A-1 sub-lineage

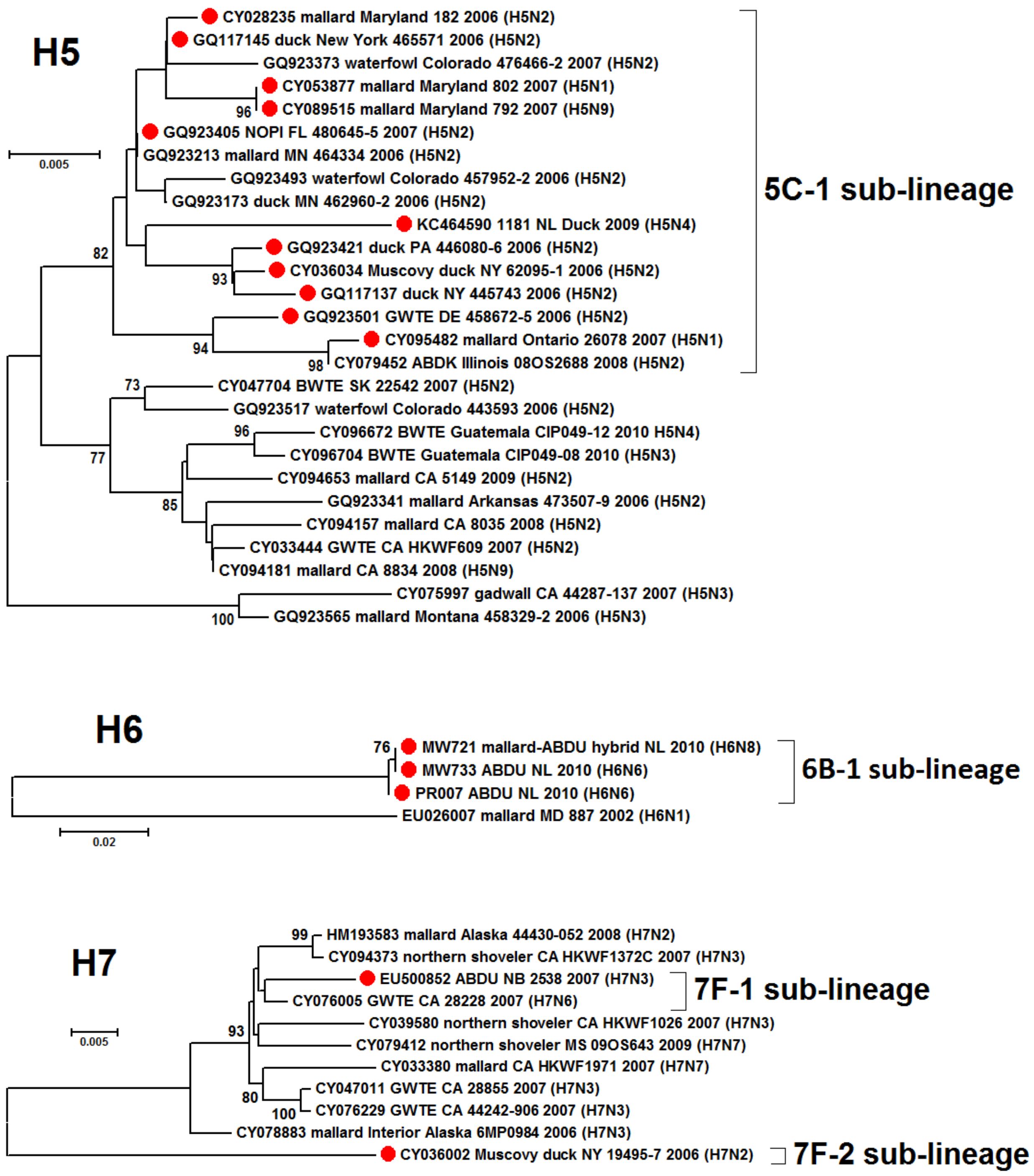
H5
CY028235 mallard Maryland 182 2006 (H5N2)
GQ117145 duck New York 465571 2006 (H5N2)
GQ923373 waterfowl Colorado 476466-2 2007 (H5N2)
CY053877 mallard Maryland 802 2007 (H5N1)
96
CY089515 mallard Maryland 792 2007 (H5N9)
GQ923405 NOPI FL 480645-5 2007 (H5N2)
0.005
GQ923213 mallard MN 464334 2006 (H5N2)
GQ923493 waterfowl Colorado 457952-2 2006 (H5N2)
GQ923173 duck MN 462960-2 2006 (H5N2)
5C-1 sub-lineage
KC464590 1181 NL Duck 2009 (H5N4)
82
GQ923421 duck PA 446080-6 2006 (H5N2)
93
CY036034 Muscovy duck NY 62095-1 2006 (H5N2)
GQ117137 duck NY 445743 2006 (H5N2)
GQ923501 GWTE DE 458672-5 2006 (H5N2)
94
CY095482 mallard Ontario 26078 2007 (H5N1)
98
CY079452 ABDK Illinois 08OS2688 2008 (H5N2)
73
CY047704 BWTE SK 22542 2007 (H5N2)
GQ923517 waterfowl Colorado 443593 2006 (H5N2)
96
CY096672 BWTE Guatemala CIP049-12 2010 H5N4)
77
CY096704 BWTE Guatemala CIP049-08 2010 (H5N3)
CY094653 mallard CA 5149 2009 (H5N2)
85
GQ923341 mallard Arkansas 473507-9 2006 (H5N2)
CY094157 mallard CA 8035 2008 (H5N2)
CY033444 GWTE CA HKWF609 2007 (H5N2)
CY094181 mallard CA 8834 2008 (H5N9)
CY075997 gadwall CA 44287-137 2007 (H5N3)
100
GQ923565 mallard Montana 458329-2 2006 (H5N3)
H6
76
MW721 mallard-ABDU hybrid NL 2010 (H6N8)
MW733 ABDU NL 2010 (H6N6)
6B-1 sub-lineage
PR007 ABDU NL 2010 (H6N6)
EU026007 mallard MD 887 2002 (H6N1)
0.02
H7
99
HM193583 mallard Alaska 44430-052 2008 (H7N2)
CY094373 northern shoveler CA HKWF1372C 2007 (H7N3)
EU500852 ABDU NB 2538 2007 (H7N3)
7F-1 sub-lineage
CY076005 GWTE CA 28228 2007 (H7N6)
0.005
93
CY039580 northern shoveler CA HKWF1026 2007 (H7N3)
CY079412 northern shoveler MS 09OS643 2009 (H7N7)
CY033380 mallard CA HKWF1971 2007 (H7N7)
80
CY047011 GWTE CA 28855 2007 (H7N3)
100
CY076229 GWTE CA 44242-906 2007 (H7N3)
CY078883 mallard Interior Alaska 6MP0984 2006 (H7N3)
CY036002 Muscovy duck NY 19495-7 2006 (H7N2)
7F-2 sub-lineage

H11

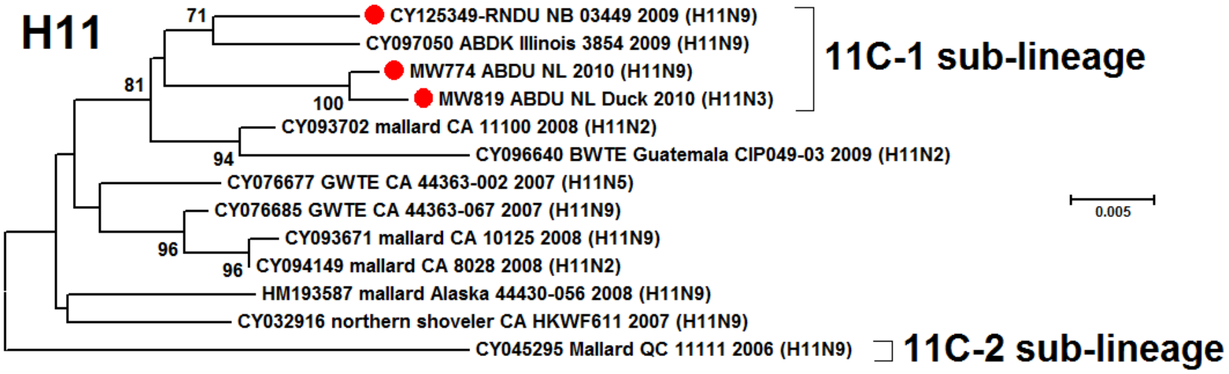

H12

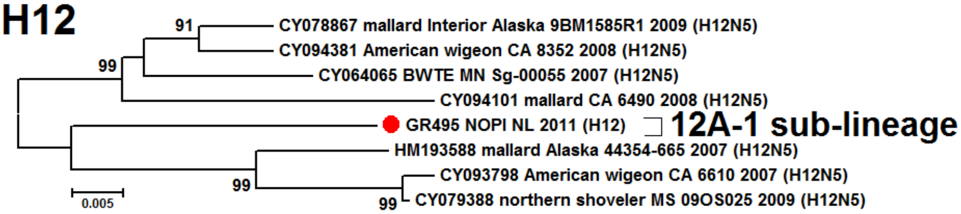

H13

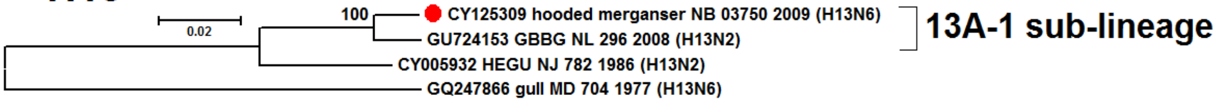

H16

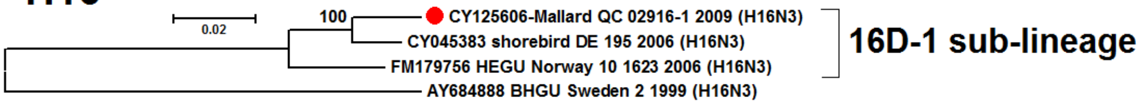

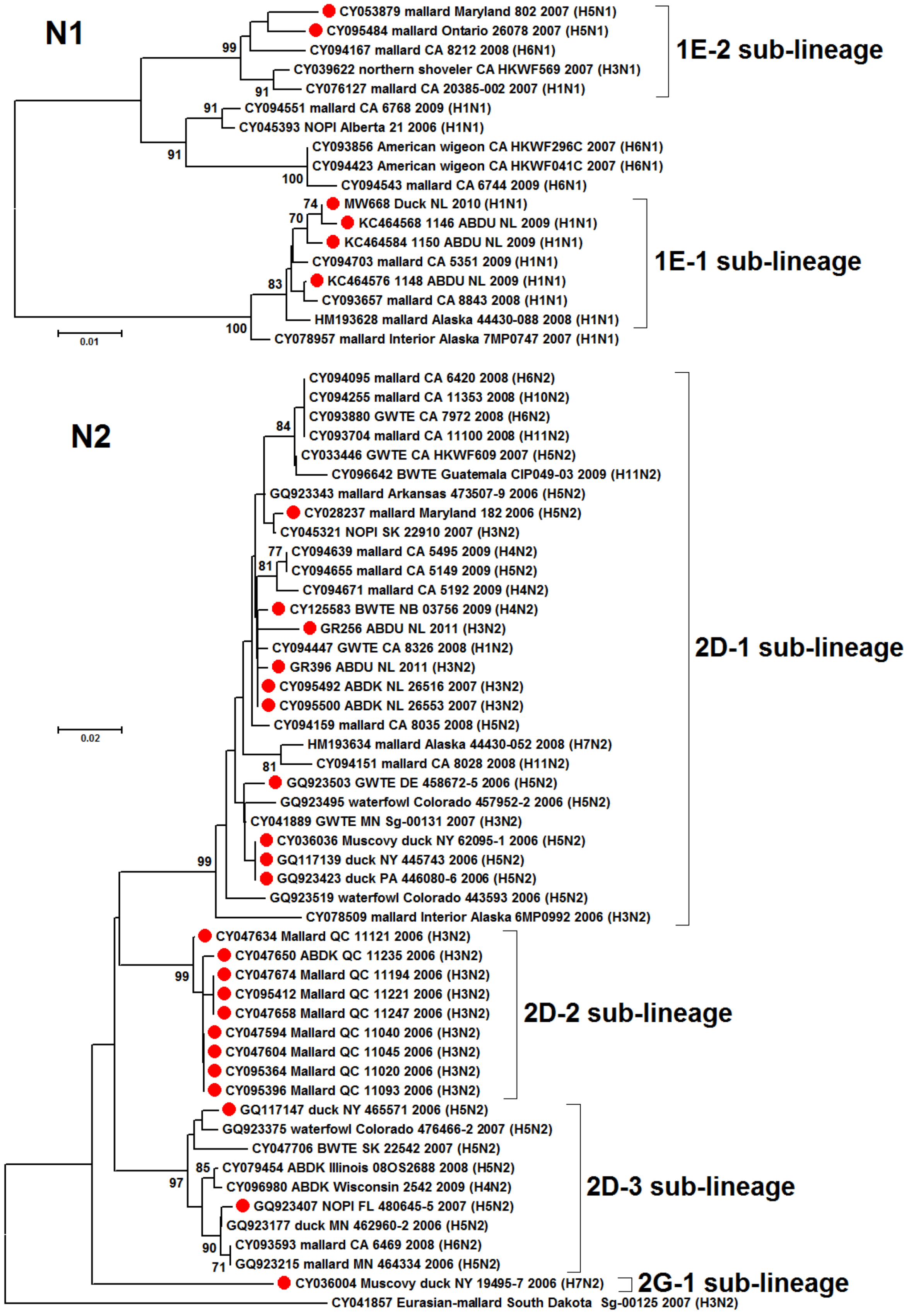
N1
CY053879 mallard Maryland 802 2007 (H5N1)
CY095484 mallard Ontario 26078 2007 (H5N1)
CY094167 mallard CA 8212 2008 (H6N1)
CY039622 northern shoveler CA HKWF569 2007 (H3N1)
CY076127 mallard CA 20385-002 2007 (H1N1)
1E-2 sub-lineage
CY094551 mallard CA 6768 2009 (H1N1)
CY045393 NOPI Alberta 21 2006 (H1N1)
CY093856 American wigeon CA HKWF296C 2007 (H6N1)
CY094423 American wigeon CA HKWF041C 2007 (H6N1)
CY094543 mallard CA 6744 2009 (H6N1)
MW668 Duck NL 2010 (H1N1)
KC464568 1146 ABDU NL 2009 (H1N1)
KC464584 1150 ABDU NL 2009 (H1N1)
CY094703 mallard CA 5351 2009 (H1N1)
KC464576 1148 ABDU NL 2009 (H1N1)
CY093657 mallard CA 8843 2008 (H1N1)
HM193628 mallard Alaska 44430-088 2008 (H1N1)
CY078957 mallard Interior Alaska 7MP0747 2007 (H1N1)
1E-1 sub-lineage
99
91
91
91
100
74
70
83
100
0.01
N2
CY094095 mallard CA 6420 2008 (H6N2)
CY094255 mallard CA 11353 2008 (H10N2)
CY093880 GWTE CA 7972 2008 (H6N2)
CY093704 mallard CA 11100 2008 (H11N2)
CY033446 GWTE CA HKWF609 2007 (H5N2)
CY096642 BWTE Guatemala CIP049-03 2009 (H11N2)
GQ923343 mallard Arkansas 473507-9 2006 (H5N2)
CY028237 mallard Maryland 182 2006 (H5N2)
CY045321 NOPI SK 22910 2007 (H3N2)
CY094639 mallard CA 5495 2009 (H4N2)
CY094655 mallard CA 5149 2009 (H5N2)
CY094671 mallard CA 5192 2009 (H4N2)
CY125583 BWTE NB 03756 2009 (H4N2)
GR256 ABDU NL 2011 (H3N2)
CY094447 GWTE CA 8326 2008 (H1N2)
GR396 ABDU NL 2011 (H3N2)
CY095492 ABDK NL 26516 2007 (H3N2)
CY095500 ABDK NL 26553 2007 (H3N2)
CY094159 mallard CA 8035 2008 (H5N2)
HM193634 mallard Alaska 44430-052 2008 (H7N2)
CY094151 mallard CA 8028 2008 (H11N2)
GQ923503 GWTE DE 458672-5 2006 (H5N2)
GQ923495 waterfowl Colorado 457952-2 2006 (H5N2)
CY041889 GWTE MN Sg-00131 2007 (H3N2)
CY036036 Muscovy duck NY 62095-1 2006 (H5N2)
GQ117139 duck NY 445743 2006 (H5N2)
GQ923423 duck PA 446080-6 2006 (H5N2)
GQ923519 waterfowl Colorado 443593 2006 (H5N2)
CY078509 mallard Interior Alaska 6MP0992 2006 (H3N2)
2D-1 sub-lineage
CY047634 Mallard QC 11121 2006 (H3N2)
CY047650 ABDK QC 11235 2006 (H3N2)
CY047674 Mallard QC 11194 2006 (H3N2)
CY095412 Mallard QC 11221 2006 (H3N2)
CY047658 Mallard QC 11247 2006 (H3N2)
CY047594 Mallard QC 11040 2006 (H3N2)
CY047604 Mallard QC 11045 2006 (H3N2)
CY095364 Mallard QC 11020 2006 (H3N2)
CY095396 Mallard QC 11093 2006 (H3N2)
2D-2 sub-lineage
GQ117147 duck NY 465571 2006 (H5N2)
GQ923375 waterfowl Colorado 476466-2 2007 (H5N2)
CY047706 BWTE SK 22542 2007 (H5N2)
CY079454 ABDK Illinois 08OS2688 2008 (H5N2)
CY096980 ABDK Wisconsin 2542 2009 (H4N2)
GQ923407 NOPI FL 480645-5 2007 (H5N2)
GQ923177 duck MN 462960-2 2006 (H5N2)
CY093593 mallard CA 6469 2008 (H6N2)
GQ923215 mallard MN 464334 2006 (H5N2)
2D-3 sub-lineage
CY036004 Muscovy duck NY 19495-7 2006 (H7N2)
2G-1 sub-lineage
CY041857 Eurasian-mallard South Dakota Sg-00125 2007 (H3N2)
84
77
81
81
99
99
85
97
90
71
0.02

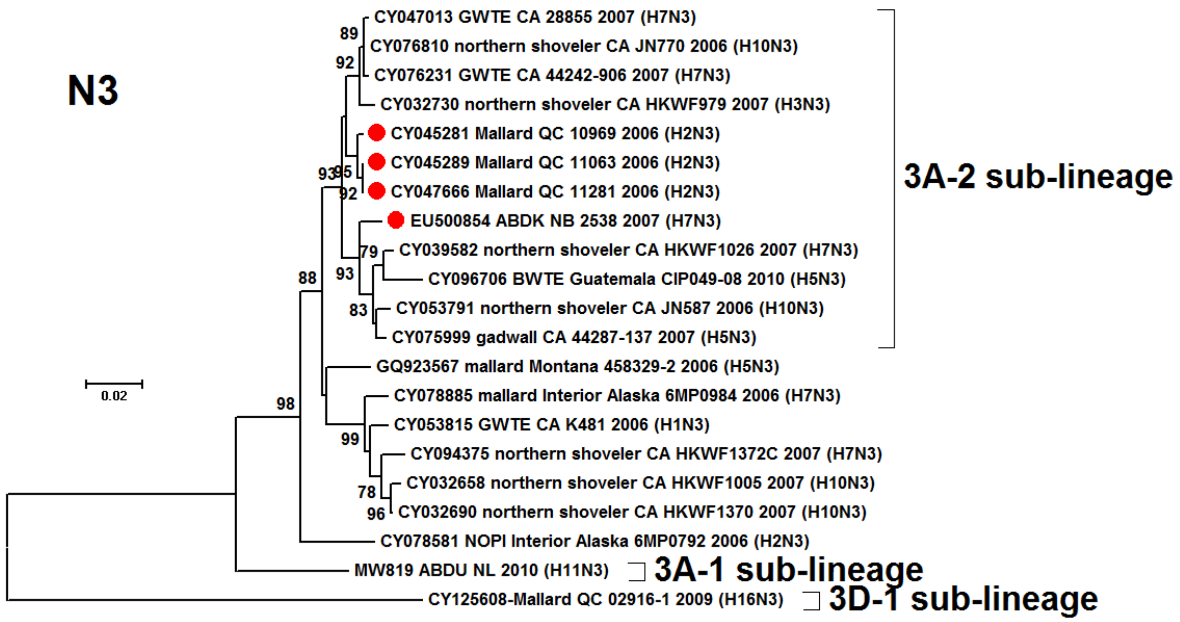
N3
CY047013 GWTE CA 28855 2007 (H7N3)
CY076810 northern shoveler CA JN770 2006 (H10N3)
CY076231 GWTE CA 44242-906 2007 (H7N3)
CY032730 northern shoveler CA HKWF979 2007 (H3N3)
CY045281 Mallard QC 10969 2006 (H2N3)
CY045289 Mallard QC 11063 2006 (H2N3)
CY047666 Mallard QC 11281 2006 (H2N3)
EU500854 ABDK NB 2538 2007 (H7N3)
CY039582 northern shoveler CA HKWF1026 2007 (H7N3)
CY096706 BWTE Guatemala CIP049-08 2010 (H5N3)
CY053791 northern shoveler CA JN587 2006 (H10N3)
CY075999 gadwall CA 44287-137 2007 (H5N3)
GQ923567 mallard Montana 458329-2 2006 (H5N3)
CY078885 mallard Interior Alaska 6MP0984 2006 (H7N3)
CY053815 GWTE CA K481 2006 (H1N3)
CY094375 northern shoveler CA HKWF1372C 2007 (H7N3)
CY032658 northern shoveler CA HKWF1005 2007 (H10N3)
CY032690 northern shoveler CA HKWF1370 2007 (H10N3)
CY078581 NOPI Interior Alaska 6MP0792 2006 (H2N3)
MW819 ABDU NL 2010 (H11N3)
CY125608-Mallard QC 02916-1 2009 (H16N3)
3A-2 sub-lineage
3A-1 sub-lineage
3D-1 sub-lineage
0.02

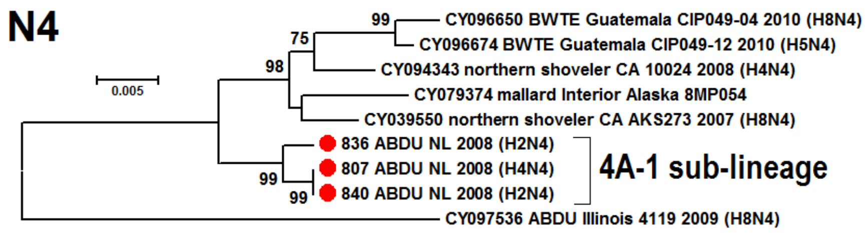
N4
CY096650 BWTE Guatemala CIP049-04 2010 (H8N4)
CY096674 BWTE Guatemala CIP049-12 2010 (H5N4)
CY094343 northern shoveler CA 10024 2008 (H4N4)
CY079374 mallard Interior Alaska 8MP054
CY039550 northern shoveler CA AKS273 2007 (H8N4)
836 ABDU NL 2008 (H2N4)
807 ABDU NL 2008 (H4N4)
840 ABDU NL 2008 (H2N4)
CY097536 ABDU Illinois 4119 2009 (H8N4)
4A-1 sub-lineage
0.005

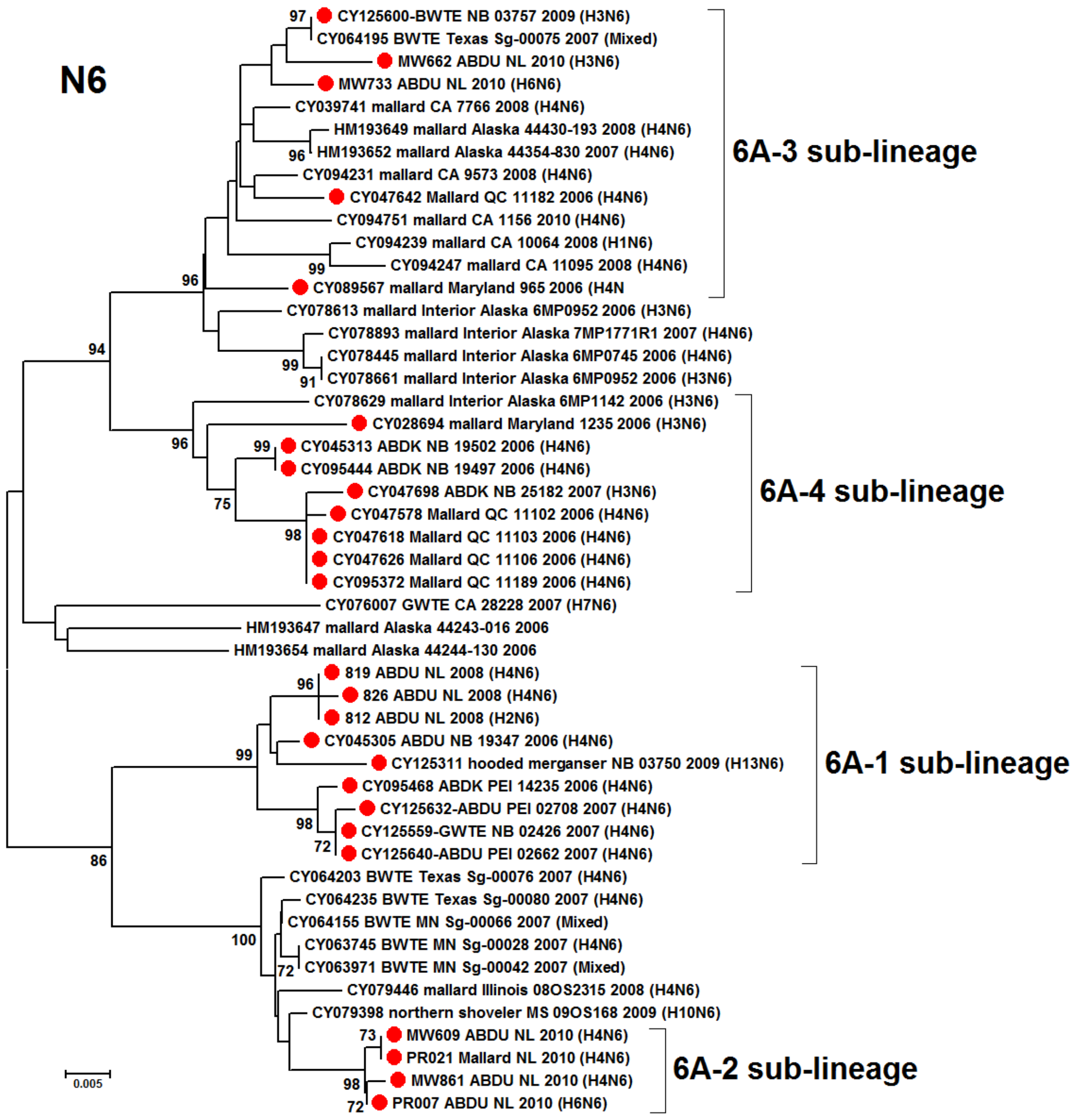
N6
CY125600-BWTE NB 03757 2009 (H3N6)
CY064195 BWTE Texas Sg-00075 2007 (Mixed)
MW662 ABDU NL 2010 (H3N6)
MW733 ABDU NL 2010 (H6N6)
CY039741 mallard CA 7766 2008 (H4N6)
HM193649 mallard Alaska 44430-193 2008 (H4N6)
HM193652 mallard Alaska 44354-830 2007 (H4N6)
CY094231 mallard CA 9573 2008 (H4N6)
CY047642 Mallard QC 11182 2006 (H4N6)
CY094751 mallard CA 1156 2010 (H4N6)
CY094239 mallard CA 10064 2008 (H1N6)
CY094247 mallard CA 11095 2008 (H4N6)
CY089567 mallard Maryland 965 2006 (H4N
CY078613 mallard Interior Alaska 6MP0952 2006 (H3N6)
CY078893 mallard Interior Alaska 7MP1771R1 2007 (H4N6)
CY078445 mallard Interior Alaska 6MP0745 2006 (H4N6)
CY078661 mallard Interior Alaska 6MP0952 2006 (H3N6)
6A-3 sub-lineage
CY078629 mallard Interior Alaska 6MP1142 2006 (H3N6)
CY028694 mallard Maryland 1235 2006 (H3N6)
CY045313 ABDK NB 19502 2006 (H4N6)
CY095444 ABDK NB 19497 2006 (H4N6)
CY047698 ABDK NB 25182 2007 (H3N6)
CY047578 Mallard QC 11102 2006 (H4N6)
CY047618 Mallard QC 11103 2006 (H4N6)
CY047626 Mallard QC 11106 2006 (H4N6)
CY095372 Mallard QC 11189 2006 (H4N6)
6A-4 sub-lineage
CY076007 GWTE CA 28228 2007 (H7N6)
HM193647 mallard Alaska 44243-016 2006
HM193654 mallard Alaska 44244-130 2006
819 ABDU NL 2008 (H4N6)
826 ABDU NL 2008 (H4N6)
812 ABDU NL 2008 (H2N6)
CY045305 ABDU NB 19347 2006 (H4N6)
CY125311 hooded merganser NB 03750 2009 (H13N6)
CY095468 ABDK PEI 14235 2006 (H4N6)
CY125632-ABDU PEI 02708 2007 (H4N6)
CY125559-GWTE NB 02426 2007 (H4N6)
CY125640-ABDU PEI 02662 2007 (H4N6)
6A-1 sub-lineage
CY064203 BWTE Texas Sg-00076 2007 (H4N6)
CY064235 BWTE Texas Sg-00080 2007 (H4N6)
CY064155 BWTE MN Sg-00066 2007 (Mixed)
CY063745 BWTE MN Sg-00028 2007 (H4N6)
CY063971 BWTE MN Sg-00042 2007 (Mixed)
CY079446 mallard Illinois 08OS2315 2008 (H4N6)
CY079398 northern shoveler MS 09OS168 2009 (H10N6)
MW609 ABDU NL 2010 (H4N6)
PR021 Mallard NL 2010 (H4N6)
MW861 ABDU NL 2010 (H4N6)
PR007 ABDU NL 2010 (H6N6)
6A-2 sub-lineage
97
96
99
96
94
99
91
96
99
75
98
96
99
98
72
86
100
72
73
98
72
0.005

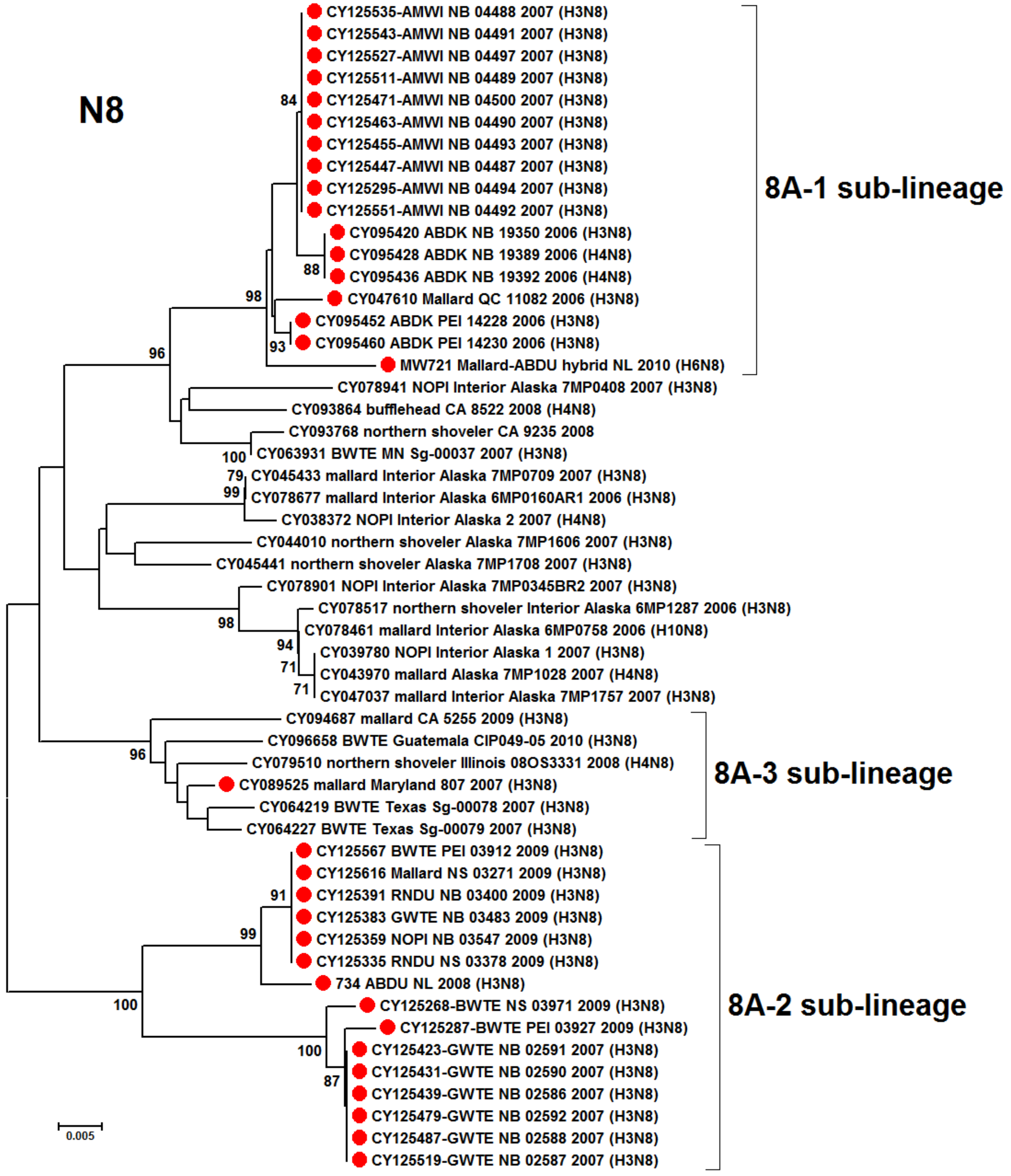
N8
CY125535-AMWI NB 04488 2007 (H3N8)
CY125543-AMWI NB 04491 2007 (H3N8)
CY125527-AMWI NB 04497 2007 (H3N8)
CY125511-AMWI NB 04489 2007 (H3N8)
84
CY125471-AMWI NB 04500 2007 (H3N8)
CY125463-AMWI NB 04490 2007 (H3N8)
CY125455-AMWI NB 04493 2007 (H3N8)
CY125447-AMWI NB 04487 2007 (H3N8)
CY125295-AMWI NB 04494 2007 (H3N8)
CY125551-AMWI NB 04492 2007 (H3N8)
CY095420 ABDK NB 19350 2006 (H3N8)
CY095428 ABDK NB 19389 2006 (H4N8)
88
CY095436 ABDK NB 19392 2006 (H4N8)
98
CY047610 Mallard QC 11082 2006 (H3N8)
CY095452 ABDK PEI 14228 2006 (H3N8)
93
CY095460 ABDK PEI 14230 2006 (H3N8)
96
MW721 Mallard-ABDU hybrid NL 2010 (H6N8)
8A-1 sub-lineage
CY078941 NOPI Interior Alaska 7MP0408 2007 (H3N8)
CY093864 bufflehead CA 8522 2008 (H4N8)
CY093768 northern shoveler CA 9235 2008
100
CY063931 BWTE MN Sg-00037 2007 (H3N8)
79
CY045433 mallard Interior Alaska 7MP0709 2007 (H3N8)
99
CY078677 mallard Interior Alaska 6MP0160AR1 2006 (H3N8)
CY038372 NOPI Interior Alaska 2 2007 (H4N8)
CY044010 northern shoveler Alaska 7MP1606 2007 (H3N8)
CY045441 northern shoveler Alaska 7MP1708 2007 (H3N8)
CY078901 NOPI Interior Alaska 7MP0345BR2 2007 (H3N8)
CY078517 northern shoveler Interior Alaska 6MP1287 2006 (H3N8)
98
CY078461 mallard Interior Alaska 6MP0758 2006 (H10N8)
94
CY039780 NOPI Interior Alaska 1 2007 (H3N8)
71
CY043970 mallard Alaska 7MP1028 2007 (H4N8)
71
CY047037 mallard Interior Alaska 7MP1757 2007 (H3N8)
CY094687 mallard CA 5255 2009 (H3N8)
CY096658 BWTE Guatemala CIP049-05 2010 (H3N8)
96
CY079510 northern shoveler Illinois 08OS3331 2008 (H4N8)
CY089525 mallard Maryland 807 2007 (H3N8)
CY064219 BWTE Texas Sg-00078 2007 (H3N8)
CY064227 BWTE Texas Sg-00079 2007 (H3N8)
8A-3 sub-lineage
CY125567 BWTE PEI 03912 2009 (H3N8)
CY125616 Mallard NS 03271 2009 (H3N8)
91
CY125391 RNDU NB 03400 2009 (H3N8)
CY125383 GWTE NB 03483 2009 (H3N8)
99
CY125359 NOPI NB 03547 2009 (H3N8)
CY125335 RNDU NS 03378 2009 (H3N8)
734 ABDU NL 2008 (H3N8)
100
CY125268-BWTE NS 03971 2009 (H3N8)
CY125287-BWTE PEI 03927 2009 (H3N8)
100
CY125423-GWTE NB 02591 2007 (H3N8)
CY125431-GWTE NB 02590 2007 (H3N8)
87
CY125439-GWTE NB 02586 2007 (H3N8)
CY125479-GWTE NB 02592 2007 (H3N8)
CY125487-GWTE NB 02588 2007 (H3N8)
CY125519-GWTE NB 02587 2007 (H3N8)
8A-2 sub-lineage
0.005

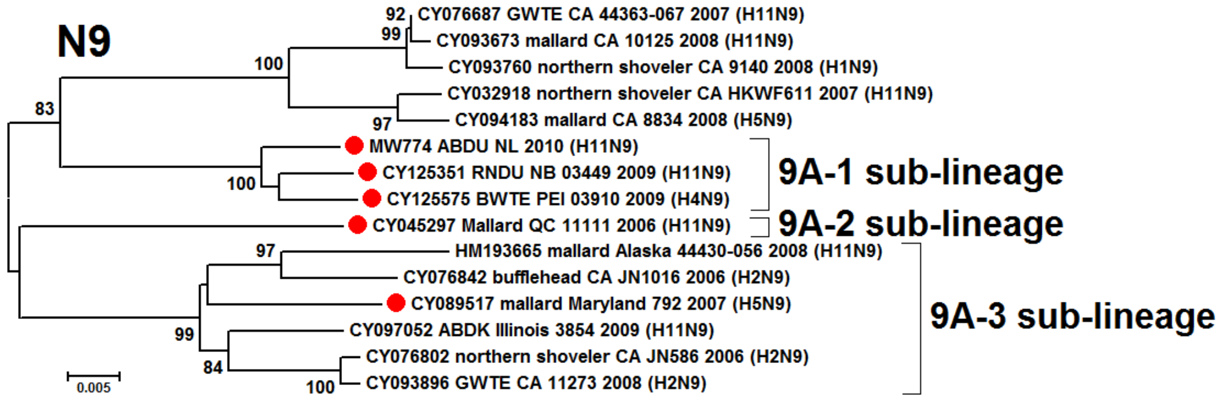
N9
92
CY076687 GWTE CA 44363-067 2007 (H11N9)
99
CY093673 mallard CA 10125 2008 (H11N9)
100
CY093760 northern shoveler CA 9140 2008 (H1N9)
83
CY032918 northern shoveler CA HKWF611 2007 (H11N9)
97
CY094183 mallard CA 8834 2008 (H5N9)
MW774 ABDU NL 2010 (H11N9)
CY125351 RNDU NB 03449 2009 (H11N9)
100
CY125575 BWTE PEI 03910 2009 (H4N9)
9A-1 sub-lineage
CY045297 Mallard QC 11111 2006 (H11N9)
9A-2 sub-lineage
97
HM193665 mallard Alaska 44430-056 2008 (H11N9)
CY076842 bufflehead CA JN1016 2006 (H2N9)
CY089517 mallard Maryland 792 2007 (H5N9)
99
CY097052 ABDK Illinois 3854 2009 (H11N9)
84
CY076802 northern shoveler CA JN586 2006 (H2N9)
100
CY093896 GWTE CA 11273 2008 (H2N9)
9A-3 sub-lineage
0.005

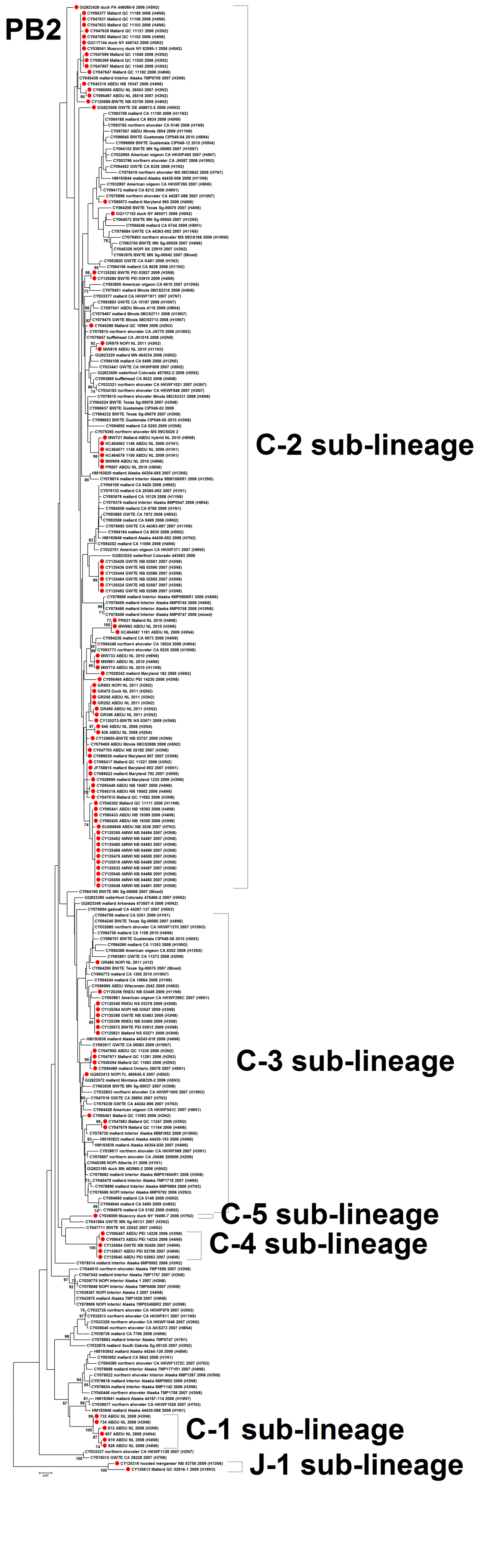
PB2
C-2 sub-lineage
C-3 sub-lineage
C-5 sub-lineage
C-4 sub-lineage
C-1 sub-lineage
J-1 sub-lineage

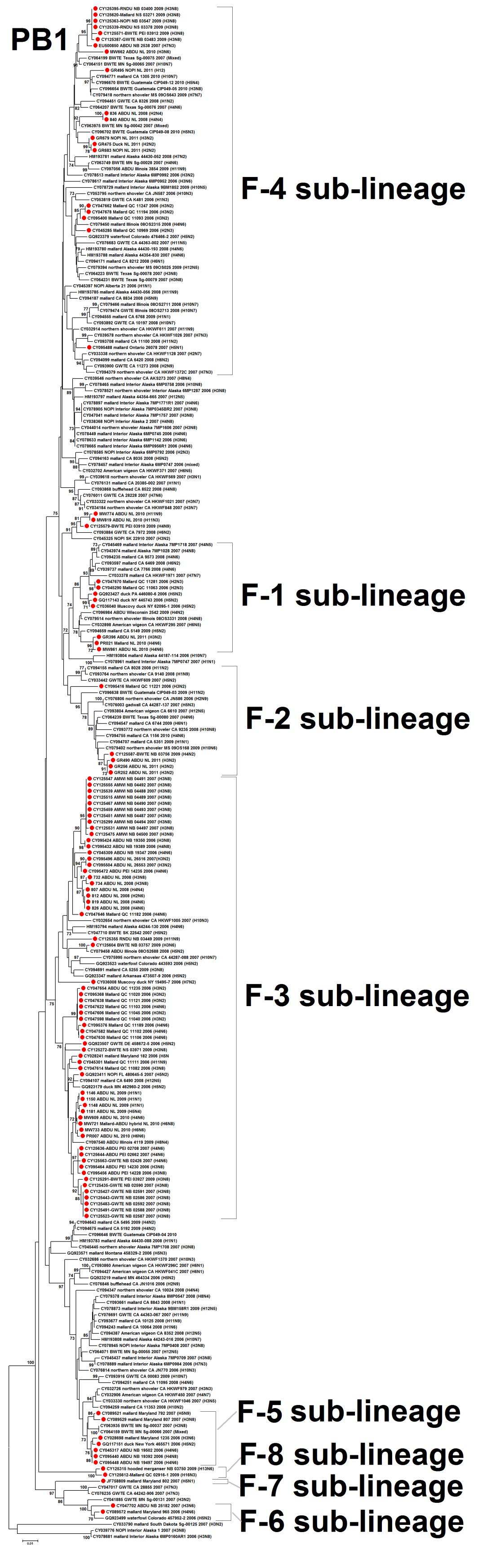
PB1
F-4 sub-lineage
F-1 sub-lineage
F-2 sub-lineage
F-3 sub-lineage
F-5 sub-lineage
F-8 sub-lineage
F-7 sub-lineage
F-6 sub-lineage

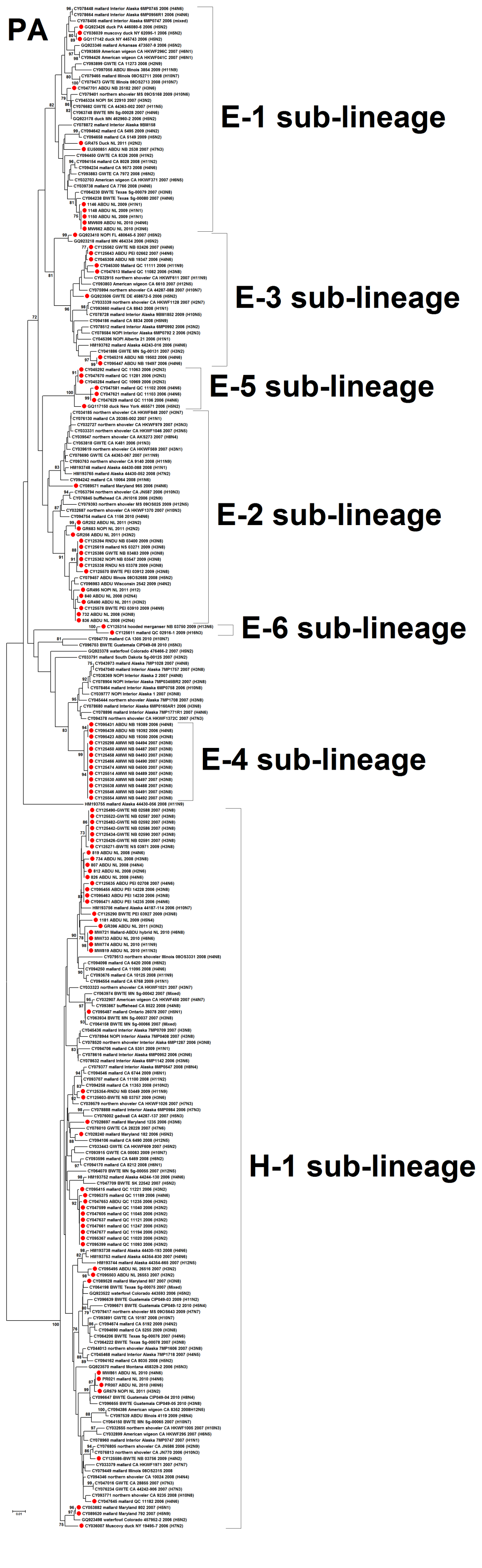
PA
E-1 sub-lineage
E-3 sub-lineage
E-5 sub-lineage
E-2 sub-lineage
E-6 sub-lineage
E-4 sub-lineage
H-1 sub-lineage

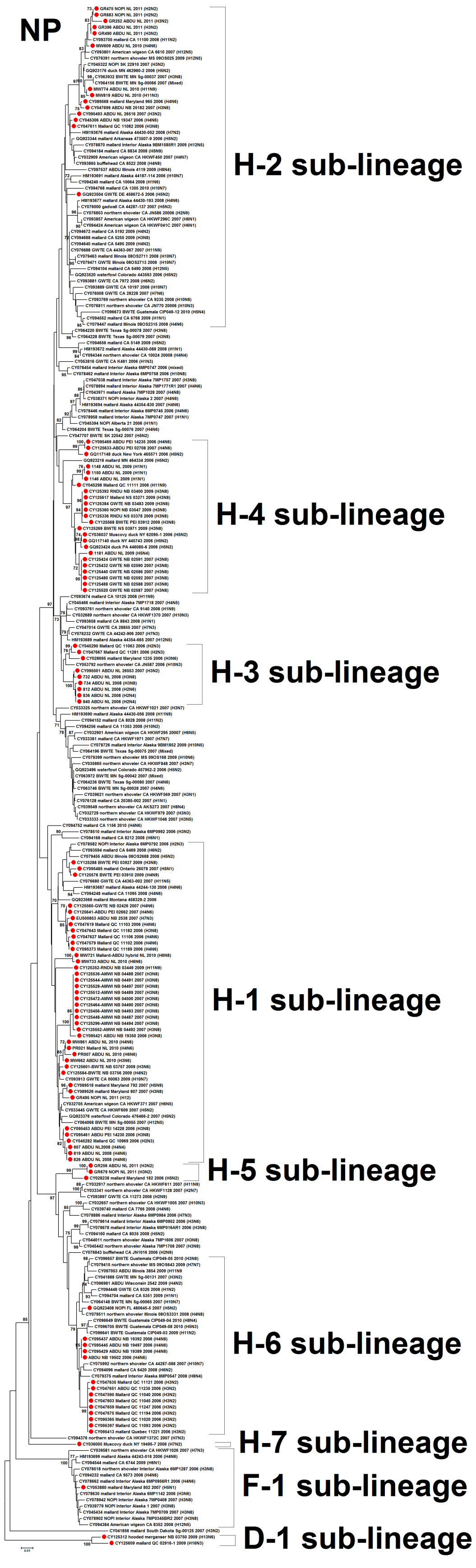
NP
H-2 sub-lineage
H-4 sub-lineage
H-3 sub-lineage
H-1 sub-lineage
H-5 sub-lineage
H-6 sub-lineage
H-7 sub-lineage
F-1 sub-lineage
D-1 sub-lineage

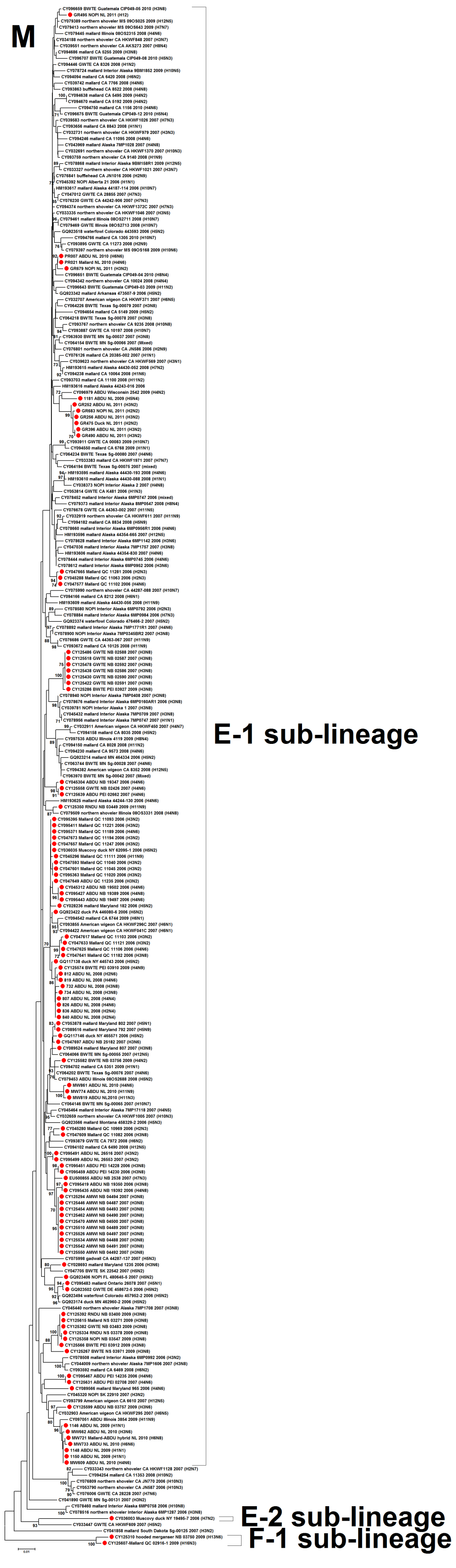
M
E-1 sub-lineage
E-2 sub-lineage
F-1 sub-lineage

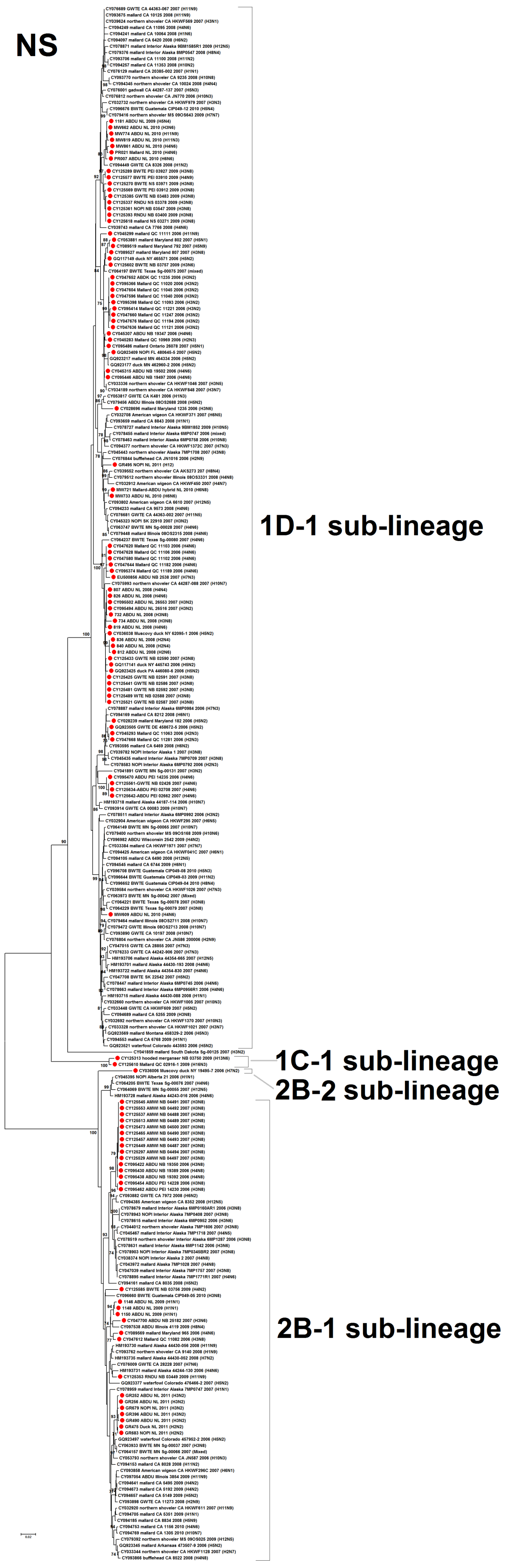
NS
1D-1 sub-lineage
1C-1 sub-lineage
2B-2 sub-lineage
2B-1 sub-lineage
