# Supplementary material for: Genetic Structure of Avian Influenza Viruses from Ducks of the Atlantic Flyway of North America
Source: PLoS One. 2014 Jan 30;9(1):e86999. doi: 10.1371/journal.pone.0086999 (PMC3907406; doi:10.1371/journal.pone.0086999)
Supplement: Figure S2 — Identification of Atlantic flyway duck AIV genes in other bird hosts. Phylogenetic trees were constructed to highlight the distribution of the genes found in the 109 Atlantic flyway duck AIVs in non-duck host species. The neighbour-joining trees were constructed with MEGA5 and support values based on 1000 bootstrap replicates are shown as percentages where ≥70%. The sub-lineages are labelled on the right of the phylogenetic trees. Black branch lines indicate detection of the sub-lineage in non-duck species, whereas grey branch lines indicate the detection of the sub-lineage only in ducks. (PDF) [file pone.0086999.s002.pdf]

Figure S2. Identification of Atlantic flyway duck AIV genes in other bird hosts.

Phylogenetic trees were constructed to highlight the distribution of the genes found in the 109 Atlantic flyway duck AIVs in non-duck host species. The neighbour-joining trees were constructed with MEGA5 and support values based on 1000 bootstrap replicates are shown as percentages where ≥70%. The sub-lineages are labelled on the right of the phylogenetic trees. Black branch lines indicate detection of the sub-lineage in non-duck species, whereas grey branch lines indicate the detection of the sub-lineage only in ducks. For the H16 tree, the 3 gull-related lineages (H16A to C) established in the Flugenome database were labelled in red.

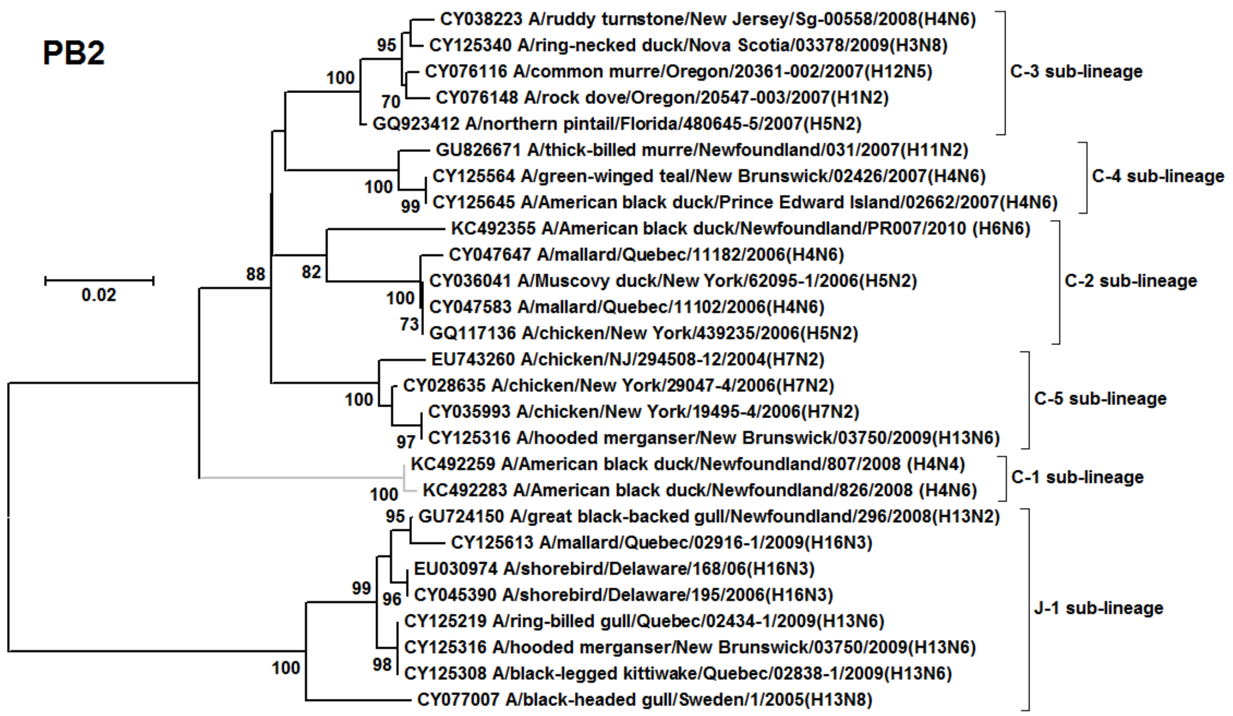
PB2
0.02
CY038223 A/ruddy turnstone/New Jersey/Sg-00558/2008(H4N6)
CY125340 A/ring-necked duck/Nova Scotia/03378/2009(H3N8)
CY076116 A/common murre/Oregon/20361-002/2007(H12N5)
CY076148 A/rock dove/Oregon/20547-003/2007(H1N2)
GQ923412 A/northern pintail/Florida/480645-5/2007(H5N2)
GU826671 A/thick-billed murre/Newfoundland/031/2007(H11N2)
CY125564 A/green-winged teal/New Brunswick/02426/2007(H4N6)
CY125645 A/American black duck/Prince Edward Island/02662/2007(H4N6)
KC492355 A/American black duck/Newfoundland/PR007/2010 (H6N6)
CY047647 A/mallard/Quebec/11182/2006(H4N6)
CY036041 A/Muscovy duck/New York/62095-1/2006(H5N2)
CY047583 A/mallard/Quebec/11102/2006(H4N6)
GQ117136 A/chicken/New York/439235/2006(H5N2)
EU743260 A/chicken/NJ/294508-12/2004(H7N2)
CY028635 A/chicken/New York/29047-4/2006(H7N2)
CY035993 A/chicken/New York/19495-4/2006(H7N2)
CY125316 A/hooded merganser/New Brunswick/03750/2009(H13N6)
KC492259 A/American black duck/Newfoundland/807/2008 (H4N4)
KC492283 A/American black duck/Newfoundland/826/2008 (H4N6)
GU724150 A/great black-backed gull/Newfoundland/296/2008(H13N2)
CY125613 A/mallard/Quebec/02916-1/2009(H16N3)
EU030974 A/shorebird/Delaware/168/06(H16N3)
CY045390 A/shorebird/Delaware/195/2006(H16N3)
CY125219 A/ring-billed gull/Quebec/02434-1/2009(H13N6)
CY125316 A/hooded merganser/New Brunswick/03750/2009(H13N6)
CY125308 A/black-legged kittiwake/Quebec/02838-1/2009(H13N6)
CY077007 A/black-headed gull/Sweden/1/2005(H13N8)
C-3 sub-lineage
C-4 sub-lineage
C-2 sub-lineage
C-5 sub-lineage
C-1 sub-lineage
J-1 sub-lineage
95
100
70
100
99
88
82
100
73
100
97
100
95
99
96
98
100

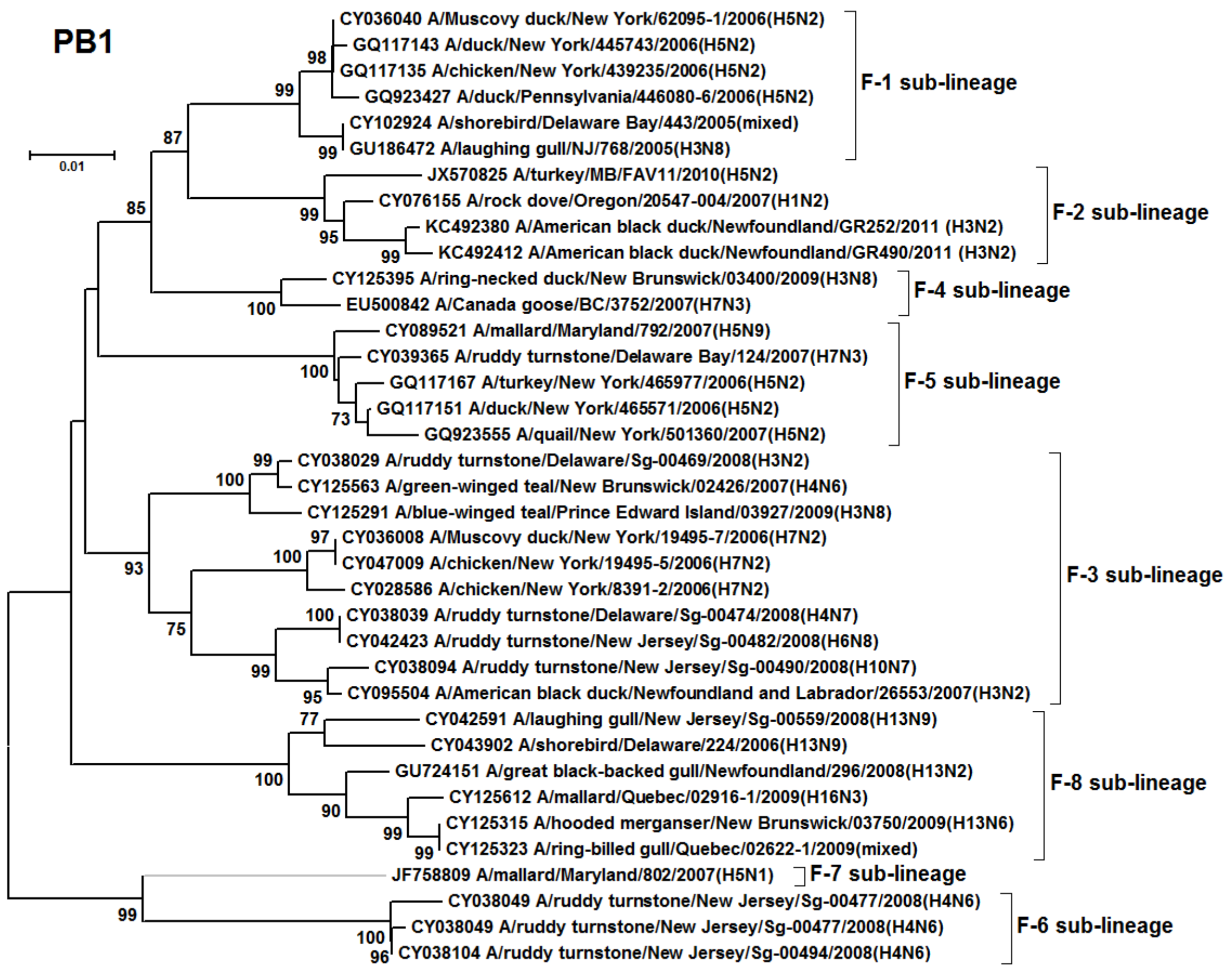
PB1
0.01
CY036040 A/Muscovy duck/New York/62095-1/2006(H5N2)
GQ117143 A/duck/New York/445743/2006(H5N2)
GQ117135 A/chicken/New York/439235/2006(H5N2)
GQ923427 A/duck/Pennsylvania/446080-6/2006(H5N2)
CY102924 A/shorebird/Delaware Bay/443/2005(mixed)
GU186472 A/laughing gull/NJ/768/2005(H3N8)
F-1 sub-lineage
JX570825 A/turkey/MB/FAV11/2010(H5N2)
CY076155 A/rock dove/Oregon/20547-004/2007(H1N2)
KC492380 A/American black duck/Newfoundland/GR252/2011 (H3N2)
KC492412 A/American black duck/Newfoundland/GR490/2011 (H3N2)
F-2 sub-lineage
CY125395 A/ring-necked duck/New Brunswick/03400/2009(H3N8)
EU500842 A/Canada goose/BC/3752/2007(H7N3)
F-4 sub-lineage
CY089521 A/mallard/Maryland/792/2007(H5N9)
CY039365 A/ruddy turnstone/Delaware Bay/124/2007(H7N3)
GQ117167 A/turkey/New York/465977/2006(H5N2)
GQ117151 A/duck/New York/465571/2006(H5N2)
GQ923555 A/quail/New York/501360/2007(H5N2)
F-5 sub-lineage
CY038029 A/ruddy turnstone/Delaware/Sg-00469/2008(H3N2)
CY125563 A/green-winged teal/New Brunswick/02426/2007(H4N6)
CY125291 A/blue-winged teal/Prince Edward Island/03927/2009(H3N8)
CY036008 A/Muscovy duck/New York/19495-7/2006(H7N2)
CY047009 A/chicken/New York/19495-5/2006(H7N2)
CY028586 A/chicken/New York/8391-2/2006(H7N2)
CY038039 A/ruddy turnstone/Delaware/Sg-00474/2008(H4N7)
CY042423 A/ruddy turnstone/New Jersey/Sg-00482/2008(H6N8)
CY038094 A/ruddy turnstone/New Jersey/Sg-00490/2008(H10N7)
CY095504 A/American black duck/Newfoundland and Labrador/26553/2007(H3N2)
F-3 sub-lineage
CY042591 A/laughing gull/New Jersey/Sg-00559/2008(H13N9)
CY043902 A/shorebird/Delaware/224/2006(H13N9)
GU724151 A/great black-backed gull/Newfoundland/296/2008(H13N2)
CY125612 A/mallard/Quebec/02916-1/2009(H16N3)
CY125315 A/hooded merganser/New Brunswick/03750/2009(H13N6)
CY125323 A/ring-billed gull/Quebec/02622-1/2009(mixed)
F-8 sub-lineage
JF758809 A/mallard/Maryland/802/2007(H5N1)
F-7 sub-lineage
CY038049 A/ruddy turnstone/New Jersey/Sg-00477/2008(H4N6)
CY038049 A/ruddy turnstone/New Jersey/Sg-00477/2008(H4N6)
CY038104 A/ruddy turnstone/New Jersey/Sg-00494/2008(H4N6)
F-6 sub-lineage

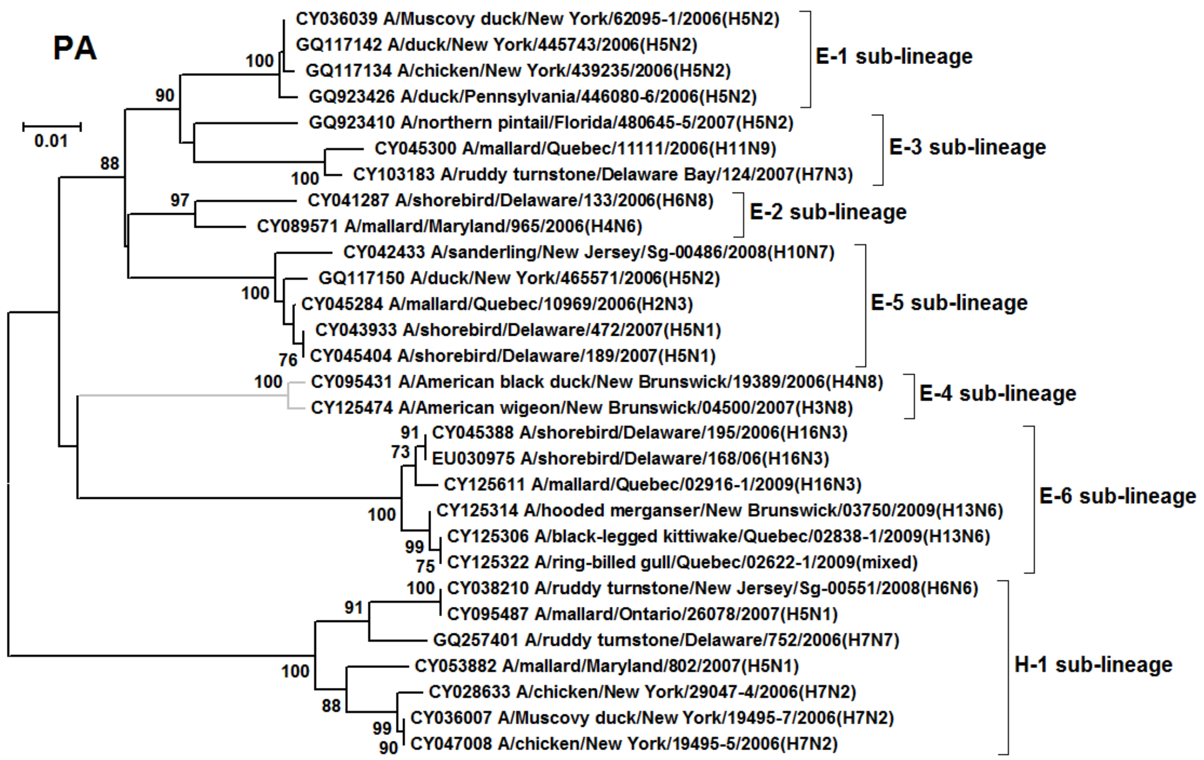
PA
0.01
CY036039 A/Muscovy duck/New York/62095-1/2006(H5N2)
GQ117142 A/duck/New York/445743/2006(H5N2)
GQ117134 A/chicken/New York/439235/2006(H5N2)
GQ923426 A/duck/Pennsylvania/446080-6/2006(H5N2)
E-1 sub-lineage
GQ923410 A/northern pintail/Florida/480645-5/2007(H5N2)
CY045300 A/mallard/Quebec/11111/2006(H11N9)
CY103183 A/ruddy turnstone/Delaware Bay/124/2007(H7N3)
E-3 sub-lineage
CY041287 A/shorebird/Delaware/133/2006(H6N8)
CY089571 A/mallard/Maryland/965/2006(H4N6)
E-2 sub-lineage
CY042433 A/sanderling/New Jersey/Sg-00486/2008(H10N7)
GQ117150 A/duck/New York/465571/2006(H5N2)
CY045284 A/mallard/Quebec/10969/2006(H2N3)
CY043933 A/shorebird/Delaware/472/2007(H5N1)
CY045404 A/shorebird/Delaware/189/2007(H5N1)
E-5 sub-lineage
CY095431 A/American black duck/New Brunswick/19389/2006(H4N8)
CY125474 A/American wigeon/New Brunswick/04500/2007(H3N8)
E-4 sub-lineage
CY045388 A/shorebird/Delaware/195/2006(H16N3)
EU030975 A/shorebird/Delaware/168/06(H16N3)
CY125611 A/mallard/Quebec/02916-1/2009(H16N3)
CY125314 A/hooded merganser/New Brunswick/03750/2009(H13N6)
CY125306 A/black-legged kittiwake/Quebec/02838-1/2009(H13N6)
CY125322 A/ring-billed gull/Quebec/02622-1/2009(mixed)
E-6 sub-lineage
CY038210 A/ruddy turnstone/New Jersey/Sg-00551/2008(H6N6)
CY095487 A/mallard/Ontario/26078/2007(H5N1)
GQ257401 A/ruddy turnstone/Delaware/752/2006(H7N7)
CY053882 A/mallard/Maryland/802/2007(H5N1)
CY028633 A/chicken/New York/29047-4/2006(H7N2)
CY036007 A/Muscovy duck/New York/19495-7/2006(H7N2)
CY047008 A/chicken/New York/19495-5/2006(H7N2)
H-1 sub-lineage
100
90
88
100
97
100
76
100
91
73
100
99
75
100
91
100
88
99
90

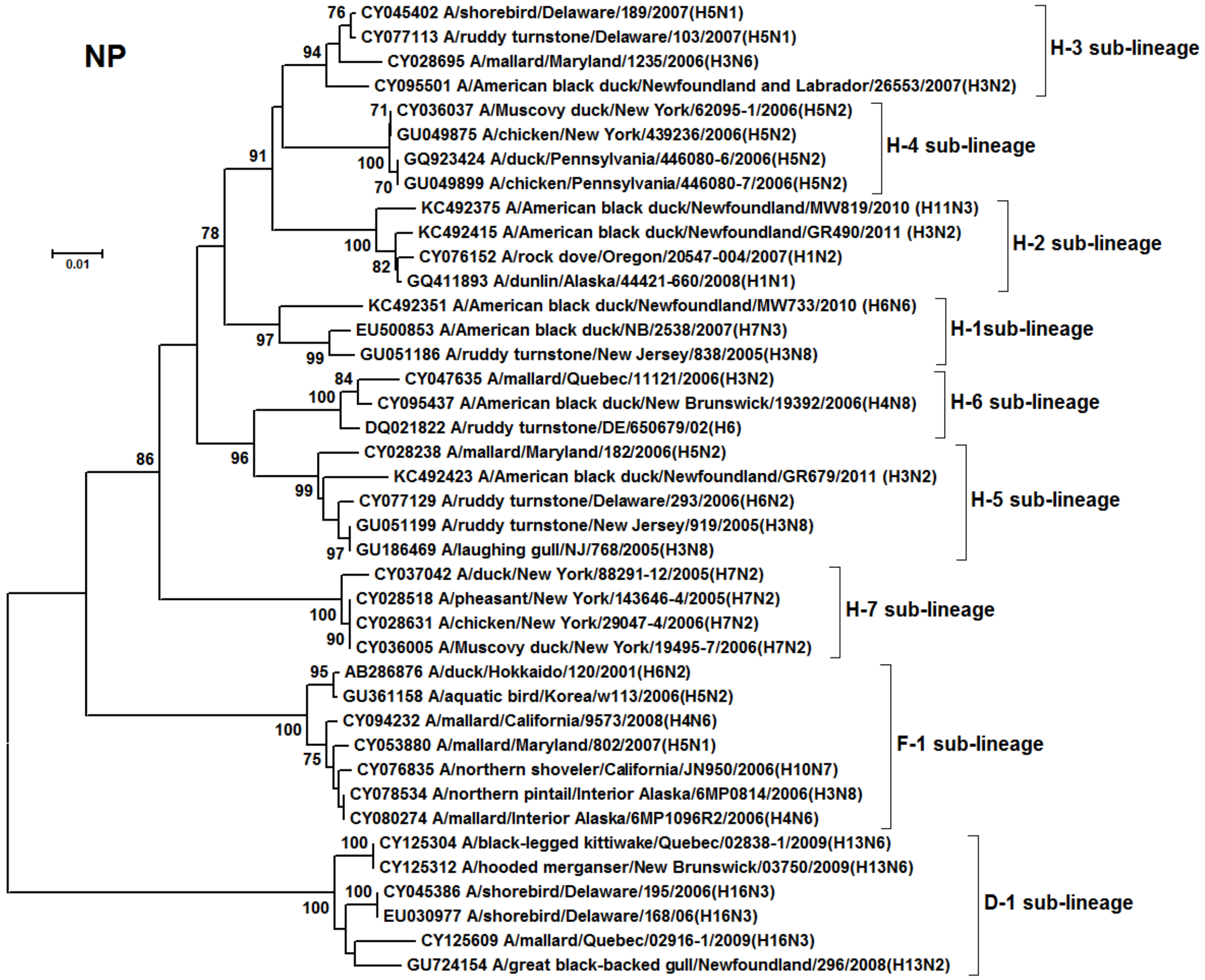
NP
CY045402 A/shorebird/Delaware/189/2007(H5N1)
CY077113 A/ruddy turnstone/Delaware/103/2007(H5N1)
CY028695 A/mallard/Maryland/1235/2006(H3N6)
CY095501 A/American black duck/Newfoundland and Labrador/26553/2007(H3N2)
H-3 sub-lineage
CY036037 A/Muscovy duck/New York/62095-1/2006(H5N2)
GU049875 A/chicken/New York/439236/2006(H5N2)
GQ923424 A/duck/Pennsylvania/446080-6/2006(H5N2)
GU049899 A/chicken/Pennsylvania/446080-7/2006(H5N2)
H-4 sub-lineage
KC492375 A/American black duck/Newfoundland/MW819/2010 (H11N3)
KC492415 A/American black duck/Newfoundland/GR490/2011 (H3N2)
CY076152 A/rock dove/Oregon/20547-004/2007(H1N2)
GQ411893 A/dunlin/Alaska/44421-660/2008(H1N1)
H-2 sub-lineage
KC492351 A/American black duck/Newfoundland/MW733/2010 (H6N6)
EU500853 A/American black duck/NB/2538/2007(H7N3)
GU051186 A/ruddy turnstone/New Jersey/838/2005(H3N8)
H-1sub-lineage
CY047635 A/mallard/Quebec/11121/2006(H3N2)
CY095437 A/American black duck/New Brunswick/19392/2006(H4N8)
DQ021822 A/ruddy turnstone/DE/650679/02(H6)
H-6 sub-lineage
CY028238 A/mallard/Maryland/182/2006(H5N2)
KC492423 A/American black duck/Newfoundland/GR679/2011 (H3N2)
CY077129 A/ruddy turnstone/Delaware/293/2006(H6N2)
GU051199 A/ruddy turnstone/New Jersey/919/2005(H3N8)
GU186469 A/laughing gull/NJ/768/2005(H3N8)
H-5 sub-lineage
CY037042 A/duck/New York/88291-12/2005(H7N2)
CY028518 A/pheasant/New York/143646-4/2005(H7N2)
CY028631 A/chicken/New York/29047-4/2006(H7N2)
CY036005 A/Muscovy duck/New York/19495-7/2006(H7N2)
H-7 sub-lineage
AB286876 A/duck/Hokkaido/120/2001(H6N2)
GU361158 A/aquatic bird/Korea/w113/2006(H5N2)
CY094232 A/mallard/California/9573/2008(H4N6)
CY053880 A/mallard/Maryland/802/2007(H5N1)
CY076835 A/northern shoveler/California/JN950/2006(H10N7)
CY078534 A/northern pintail/Interior Alaska/6MP0814/2006(H3N8)
CY080274 A/mallard/Interior Alaska/6MP1096R2/2006(H4N6)
F-1 sub-lineage
CY125304 A/black-legged kittiwake/Quebec/02838-1/2009(H13N6)
CY125312 A/hooded merganser/New Brunswick/03750/2009(H13N6)
CY045386 A/shorebird/Delaware/195/2006(H16N3)
EU030977 A/shorebird/Delaware/168/06(H16N3)
CY125609 A/mallard/Quebec/02916-1/2009(H16N3)
GU724154 A/great black-backed gull/Newfoundland/296/2008(H13N2)
D-1 sub-lineage
0.01
76
94
91
78
86
71
100
70
100
82
97
99
84
100
96
99
97
100
90
95
100
75
100
100
100

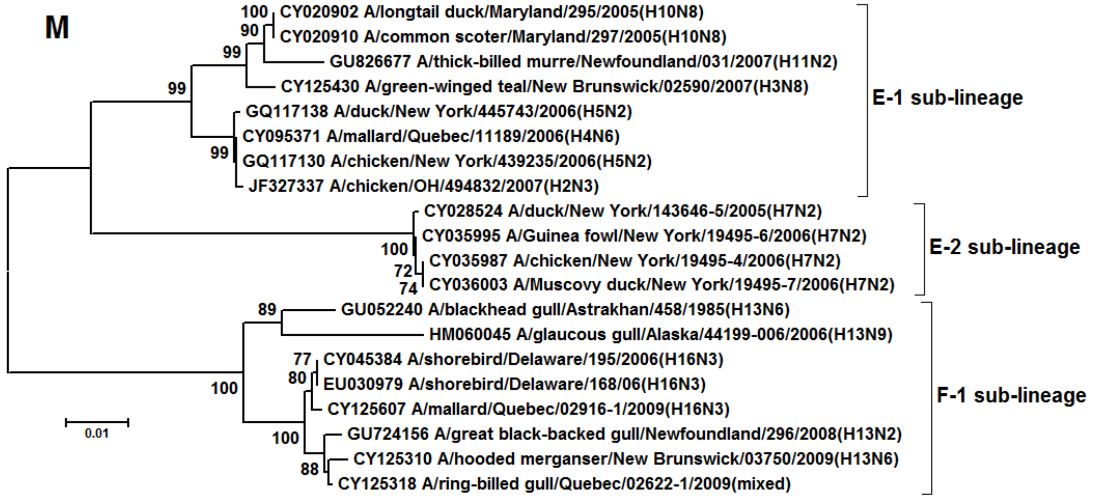
M
CY020902 A/longtail duck/Maryland/295/2005(H10N8)
CY020910 A/common scoter/Maryland/297/2005(H10N8)
GU826677 A/thick-billed murre/Newfoundland/031/2007(H11N2)
CY125430 A/green-winged teal/New Brunswick/02590/2007(H3N8)
GQ117138 A/duck/New York/445743/2006(H5N2)
CY095371 A/mallard/Quebec/11189/2006(H4N6)
GQ117130 A/chicken/New York/439235/2006(H5N2)
JF327337 A/chicken/OH/494832/2007(H2N3)
E-1 sub-lineage
CY028524 A/duck/New York/143646-5/2005(H7N2)
CY035995 A/Guinea fowl/New York/19495-6/2006(H7N2)
CY035987 A/chicken/New York/19495-4/2006(H7N2)
CY036003 A/Muscovy duck/New York/19495-7/2006(H7N2)
E-2 sub-lineage
GU052240 A/blackhead gull/Astrakhan/458/1985(H13N6)
HM060045 A/glaucous gull/Alaska/44199-006/2006(H13N9)
CY045384 A/shorebird/Delaware/195/2006(H16N3)
EU030979 A/shorebird/Delaware/168/06(H16N3)
CY125607 A/mallard/Quebec/02916-1/2009(H16N3)
GU724156 A/great black-backed gull/Newfoundland/296/2008(H13N2)
CY125310 A/hooded merganser/New Brunswick/03750/2009(H13N6)
CY125318 A/ring-billed gull/Quebec/02622-1/2009(mixed)
F-1 sub-lineage
0.01
100
90
99
99
99
100
72
74
89
77
80
100
100
88

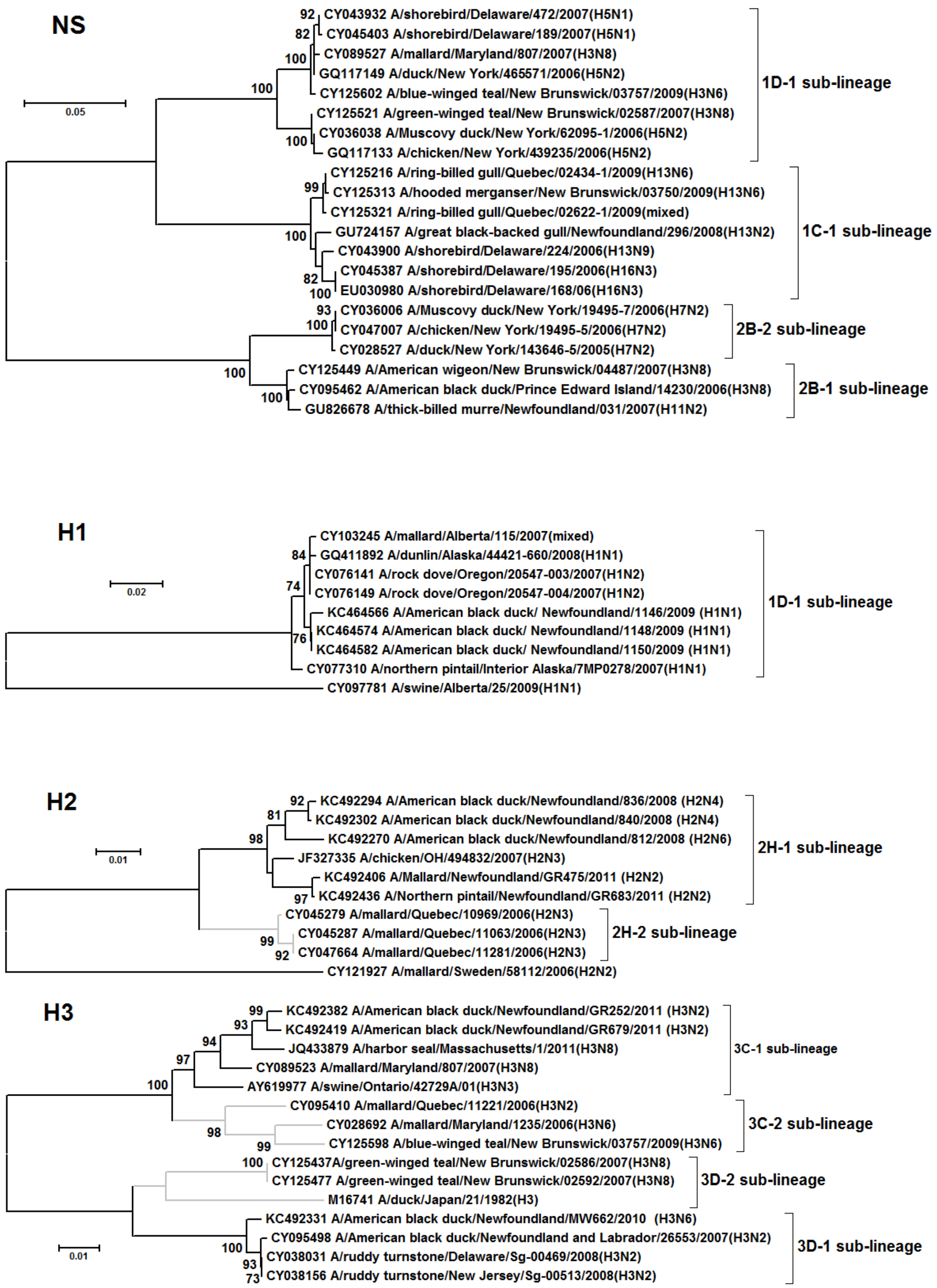
NS
0.05
CY043932 A/shorebird/Delaware/472/2007(H5N1)
CY045403 A/shorebird/Delaware/189/2007(H5N1)
CY089527 A/mallard/Maryland/807/2007(H3N8)
GQ117149 A/duck/New York/465571/2006(H5N2)
CY125602 A/blue-winged teal/New Brunswick/03757/2009(H3N6)
CY125521 A/green-winged teal/New Brunswick/02587/2007(H3N8)
CY036038 A/Muscovy duck/New York/62095-1/2006(H5N2)
GQ117133 A/chicken/New York/439235/2006(H5N2)
1D-1 sub-lineage
CY125216 A/ring-billed gull/Quebec/02434-1/2009(H13N6)
CY125313 A/hooded merganser/New Brunswick/03750/2009(H13N6)
CY125321 A/ring-billed gull/Quebec/02622-1/2009(mixed)
GU724157 A/great black-backed gull/Newfoundland/296/2008(H13N2)
CY043900 A/shorebird/Delaware/224/2006(H13N9)
CY045387 A/shorebird/Delaware/195/2006(H16N3)
EU030980 A/shorebird/Delaware/168/06(H16N3)
1C-1 sub-lineage
CY036006 A/Muscovy duck/New York/19495-7/2006(H7N2)
CY047007 A/chicken/New York/19495-5/2006(H7N2)
CY028527 A/duck/New York/143646-5/2005(H7N2)
2B-2 sub-lineage
CY125449 A/American wigeon/New Brunswick/04487/2007(H3N8)
CY095462 A/American black duck/Prince Edward Island/14230/2006(H3N8)
GU826678 A/thick-billed murre/Newfoundland/031/2007(H11N2)
2B-1 sub-lineage
92
82
100
100
100
99
100
82
100
93
100
100
100
H1
0.02
CY103245 A/mallard/Alberta/115/2007(mixed)
GQ411892 A/dunlin/Alaska/44421-660/2008(H1N1)
CY076141 A/rock dove/Oregon/20547-003/2007(H1N2)
CY076149 A/rock dove/Oregon/20547-004/2007(H1N2)
KC464566 A/American black duck/ Newfoundland/1146/2009 (H1N1)
KC464574 A/American black duck/ Newfoundland/1148/2009 (H1N1)
KC464582 A/American black duck/ Newfoundland/1150/2009 (H1N1)
CY077310 A/northern pintail/Interior Alaska/7MP0278/2007(H1N1)
CY097781 A/swine/Alberta/25/2009(H1N1)
1D-1 sub-lineage
84
74
76
H2
0.01
KC492294 A/American black duck/Newfoundland/836/2008 (H2N4)
KC492302 A/American black duck/Newfoundland/840/2008 (H2N4)
KC492270 A/American black duck/Newfoundland/812/2008 (H2N6)
JF327335 A/chicken/OH/494832/2007(H2N3)
KC492406 A/Mallard/Newfoundland/GR475/2011 (H2N2)
KC492436 A/Northern pintail/Newfoundland/GR683/2011 (H2N2)
2H-1 sub-lineage
CY045279 A/mallard/Quebec/10969/2006(H2N3)
CY045287 A/mallard/Quebec/11063/2006(H2N3)
CY047664 A/mallard/Quebec/11281/2006(H2N3)
2H-2 sub-lineage
CY121927 A/mallard/Sweden/58112/2006(H2N2)
92
81
98
97
99
92
H3
KC492382 A/American black duck/Newfoundland/GR252/2011 (H3N2)
KC492419 A/American black duck/Newfoundland/GR679/2011 (H3N2)
JQ433879 A/harbor seal/Massachusetts/1/2011(H3N8)
CY089523 A/mallard/Maryland/807/2007(H3N8)
AY619977 A/swine/Ontario/42729A/01(H3N3)
3C-1 sub-lineage
CY095410 A/mallard/Quebec/11221/2006(H3N2)
CY028692 A/mallard/Maryland/1235/2006(H3N6)
CY125598 A/blue-winged teal/New Brunswick/03757/2009(H3N6)
3C-2 sub-lineage
CY125437A/green-winged teal/New Brunswick/02586/2007(H3N8)
CY125477 A/green-winged teal/New Brunswick/02592/2007(H3N8)
M16741 A/duck/Japan/21/1982(H3)
3D-2 sub-lineage
KC492331 A/American black duck/Newfoundland/MW662/2010 (H3N6)
CY095498 A/American black duck/Newfoundland and Labrador/26553/2007(H3N2)
CY038031 A/ruddy turnstone/Delaware/Sg-00469/2008(H3N2)
CY038156 A/ruddy turnstone/New Jersey/Sg-00513/2008(H3N2)
3D-1 sub-lineage
99
93
94
97
100
98
99
100
100
93
73
0.01

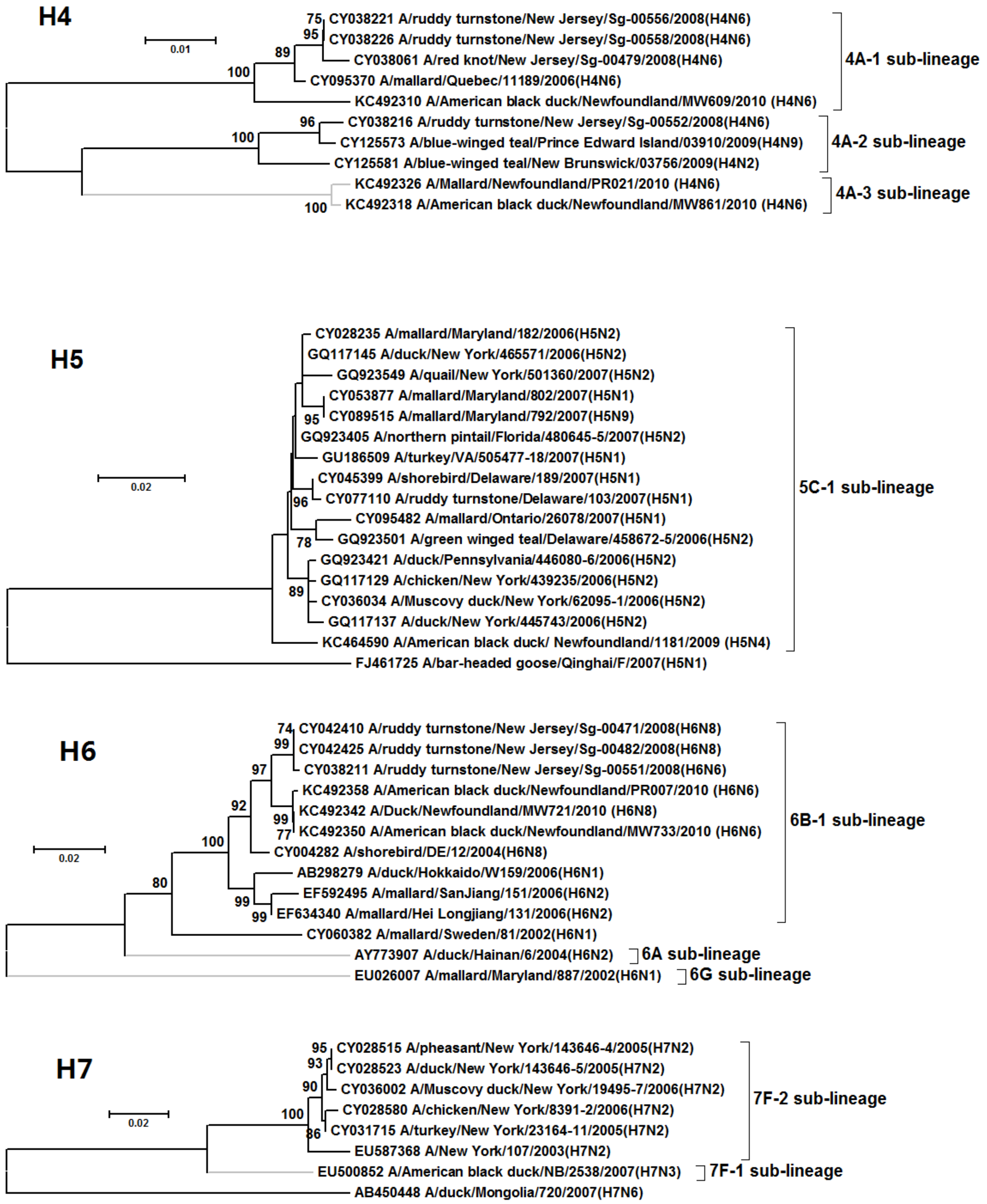
H4
0.01
CY038221 A/ruddy turnstone/New Jersey/Sg-00556/2008(H4N6)
CY038226 A/ruddy turnstone/New Jersey/Sg-00558/2008(H4N6)
CY038061 A/red knot/New Jersey/Sg-00479/2008(H4N6)
CY095370 A/mallard/Quebec/11189/2006(H4N6)
KC492310 A/American black duck/Newfoundland/MW609/2010 (H4N6)
4A-1 sub-lineage
CY038216 A/ruddy turnstone/New Jersey/Sg-00552/2008(H4N6)
CY125573 A/blue-winged teal/Prince Edward Island/03910/2009(H4N9)
CY125581 A/blue-winged teal/New Brunswick/03756/2009(H4N2)
4A-2 sub-lineage
KC492326 A/Mallard/Newfoundland/PR021/2010 (H4N6)
KC492318 A/American black duck/Newfoundland/MW861/2010 (H4N6)
4A-3 sub-lineage
H5
0.02
CY028235 A/mallard/Maryland/182/2006(H5N2)
GQ117145 A/duck/New York/465571/2006(H5N2)
GQ923549 A/quail/New York/501360/2007(H5N2)
CY053877 A/mallard/Maryland/802/2007(H5N1)
CY089515 A/mallard/Maryland/792/2007(H5N9)
GQ923405 A/northern pintail/Florida/480645-5/2007(H5N2)
GU186509 A/turkey/VA/505477-18/2007(H5N1)
CY045399 A/shorebird/Delaware/189/2007(H5N1)
CY077110 A/ruddy turnstone/Delaware/103/2007(H5N1)
CY095482 A/mallard/Ontario/26078/2007(H5N1)
GQ923501 A/green winged teal/Delaware/458672-5/2006(H5N2)
GQ923421 A/duck/Pennsylvania/446080-6/2006(H5N2)
GQ117129 A/chicken/New York/439235/2006(H5N2)
CY036034 A/Muscovy duck/New York/62095-1/2006(H5N2)
GQ117137 A/duck/New York/445743/2006(H5N2)
KC464590 A/American black duck/ Newfoundland/1181/2009 (H5N4)
FJ461725 A/bar-headed goose/Qinghai/F/2007(H5N1)
5C-1 sub-lineage
H6
0.02
CY042410 A/ruddy turnstone/New Jersey/Sg-00471/2008(H6N8)
CY042425 A/ruddy turnstone/New Jersey/Sg-00482/2008(H6N8)
CY038211 A/ruddy turnstone/New Jersey/Sg-00551/2008(H6N6)
KC492358 A/American black duck/Newfoundland/PR007/2010 (H6N6)
KC492342 A/Duck/Newfoundland/MW721/2010 (H6N8)
KC492350 A/American black duck/Newfoundland/MW733/2010 (H6N6)
CY004282 A/shorebird/DE/12/2004(H6N8)
AB298279 A/duck/Hokkaido/W159/2006(H6N1)
EF592495 A/mallard/SanJiang/151/2006(H6N2)
EF634340 A/mallard/Hei Longjiang/131/2006(H6N2)
CY060382 A/mallard/Sweden/81/2002(H6N1)
6B-1 sub-lineage
AY773907 A/duck/Hainan/6/2004(H6N2)
6A sub-lineage
EU026007 A/mallard/Maryland/887/2002(H6N1)
6G sub-lineage
H7
0.02
CY028515 A/pheasant/New York/143646-4/2005(H7N2)
CY028523 A/duck/New York/143646-5/2005(H7N2)
CY036002 A/Muscovy duck/New York/19495-7/2006(H7N2)
CY028580 A/chicken/New York/8391-2/2006(H7N2)
CY031715 A/turkey/New York/23164-11/2005(H7N2)
EU587368 A/New York/107/2003(H7N2)
7F-2 sub-lineage
EU500852 A/American black duck/NB/2538/2007(H7N3)
7F-1 sub-lineage
AB450448 A/duck/Mongolia/720/2007(H7N6)

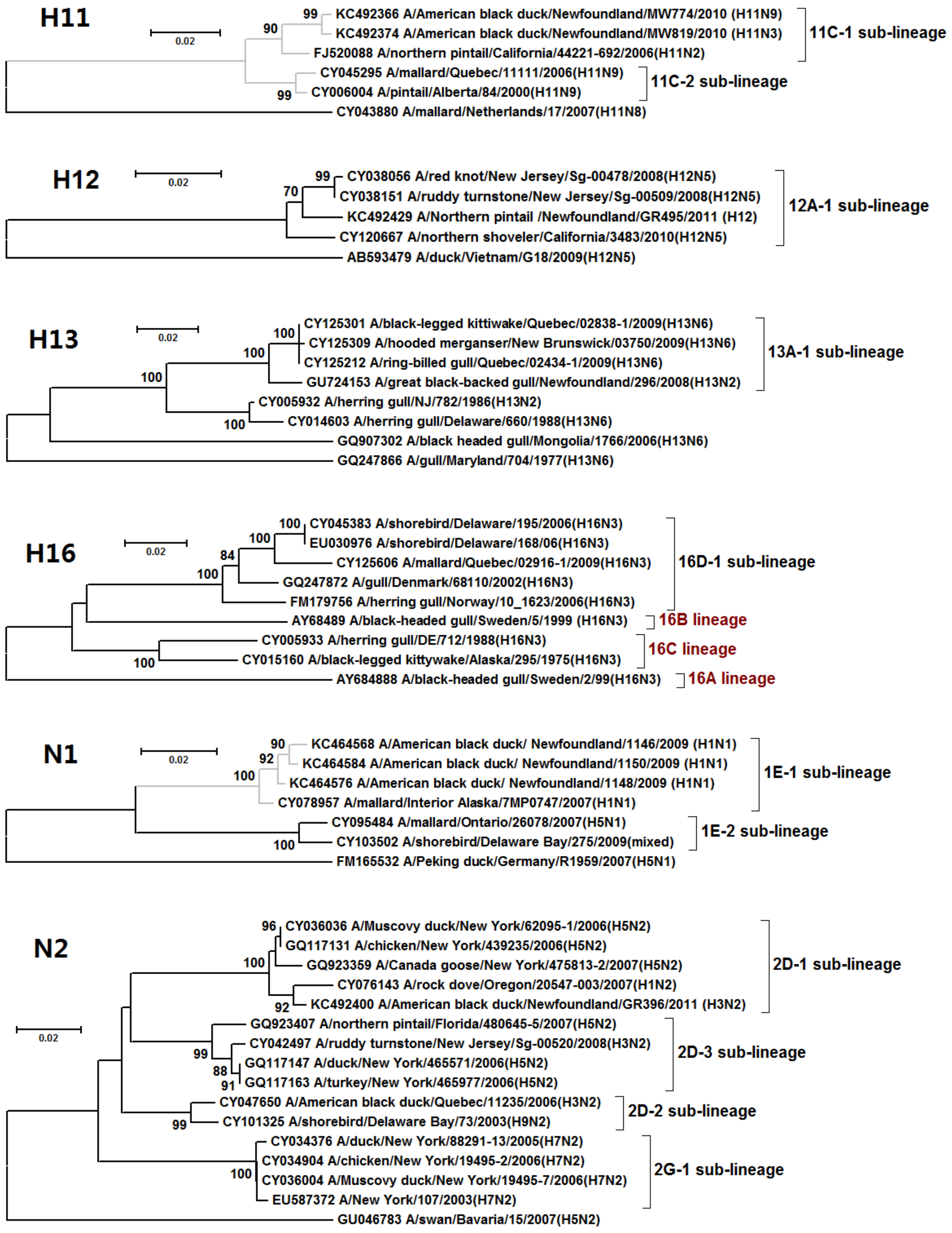
H11
0.02
KC492366 A/American black duck/Newfoundland/MW774/2010 (H11N9)
KC492374 A/American black duck/Newfoundland/MW819/2010 (H11N3)
FJ520088 A/northern pintail/California/44221-692/2006(H11N2)
CY045295 A/mallard/Quebec/11111/2006(H11N9)
CY006004 A/pintail/Alberta/84/2000(H11N9)
CY043880 A/mallard/Netherlands/17/2007(H11N8)
99
90
99
11C-1 sub-lineage
11C-2 sub-lineage
H12
0.02
CY038056 A/red knot/New Jersey/Sg-00478/2008(H12N5)
CY038151 A/ruddy turnstone/New Jersey/Sg-00509/2008(H12N5)
KC492429 A/Northern pintail /Newfoundland/GR495/2011 (H12)
CY120667 A/northern shoveler/California/3483/2010(H12N5)
AB593479 A/duck/Vietnam/G18/2009(H12N5)
99
70
12A-1 sub-lineage
H13
0.02
CY125301 A/black-legged kittiwake/Quebec/02838-1/2009(H13N6)
CY125309 A/hooded merganser/New Brunswick/03750/2009(H13N6)
CY125212 A/ring-billed gull/Quebec/02434-1/2009(H13N6)
GU724153 A/great black-backed gull/Newfoundland/296/2008(H13N2)
CY005932 A/herring gull/NJ/782/1986(H13N2)
CY014603 A/herring gull/Delaware/660/1988(H13N6)
GQ907302 A/black headed gull/Mongolia/1766/2006(H13N6)
GQ247866 A/gull/Maryland/704/1977(H13N6)
100
100
100
100
13A-1 sub-lineage
H16
0.02
CY045383 A/shorebird/Delaware/195/2006(H16N3)
EU030976 A/shorebird/Delaware/168/06(H16N3)
CY125606 A/mallard/Quebec/02916-1/2009(H16N3)
GQ247872 A/gull/Denmark/68110/2002(H16N3)
FM179756 A/herring gull/Norway/10_1623/2006(H16N3)
AY68489 A/black-headed gull/Sweden/5/1999 (H16N3)
CY005933 A/herring gull/DE/712/1988(H16N3)
CY015160 A/black-legged kittywake/Alaska/295/1975(H16N3)
AY684888 A/black-headed gull/Sweden/2/99(H16N3)
100
100
84
100
100
16D-1 sub-lineage
16B lineage
16C lineage
16A lineage
N1
0.02
KC464568 A/American black duck/ Newfoundland/1146/2009 (H1N1)
KC464584 A/American black duck/ Newfoundland/1150/2009 (H1N1)
KC464576 A/American black duck/ Newfoundland/1148/2009 (H1N1)
CY078957 A/mallard/Interior Alaska/7MP0747/2007(H1N1)
CY095484 A/mallard/Ontario/26078/2007(H5N1)
CY103502 A/shorebird/Delaware Bay/275/2009(mixed)
FM165532 A/Peking duck/Germany/R1959/2007(H5N1)
90
92
100
100
1E-1 sub-lineage
1E-2 sub-lineage
N2
0.02
CY036036 A/Muscovy duck/New York/62095-1/2006(H5N2)
GQ117131 A/chicken/New York/439235/2006(H5N2)
GQ923359 A/Canada goose/New York/475813-2/2007(H5N2)
CY076143 A/rock dove/Oregon/20547-003/2007(H1N2)
KC492400 A/American black duck/Newfoundland/GR396/2011 (H3N2)
GQ923407 A/northern pintail/Florida/480645-5/2007(H5N2)
CY042497 A/ruddy turnstone/New Jersey/Sg-00520/2008(H3N2)
GQ117147 A/duck/New York/465571/2006(H5N2)
GQ117163 A/turkey/New York/465977/2006(H5N2)
CY047650 A/American black duck/Quebec/11235/2006(H3N2)
CY101325 A/shorebird/Delaware Bay/73/2003(H9N2)
CY034376 A/duck/New York/88291-13/2005(H7N2)
CY034904 A/chicken/New York/19495-2/2006(H7N2)
CY036004 A/Muscovy duck/New York/19495-7/2006(H7N2)
EU587372 A/New York/107/2003(H7N2)
GU046783 A/swan/Bavaria/15/2007(H5N2)
96
100
92
99
88
91
99
100
2D-1 sub-lineage
2D-3 sub-lineage
2D-2 sub-lineage
2G-1 sub-lineage

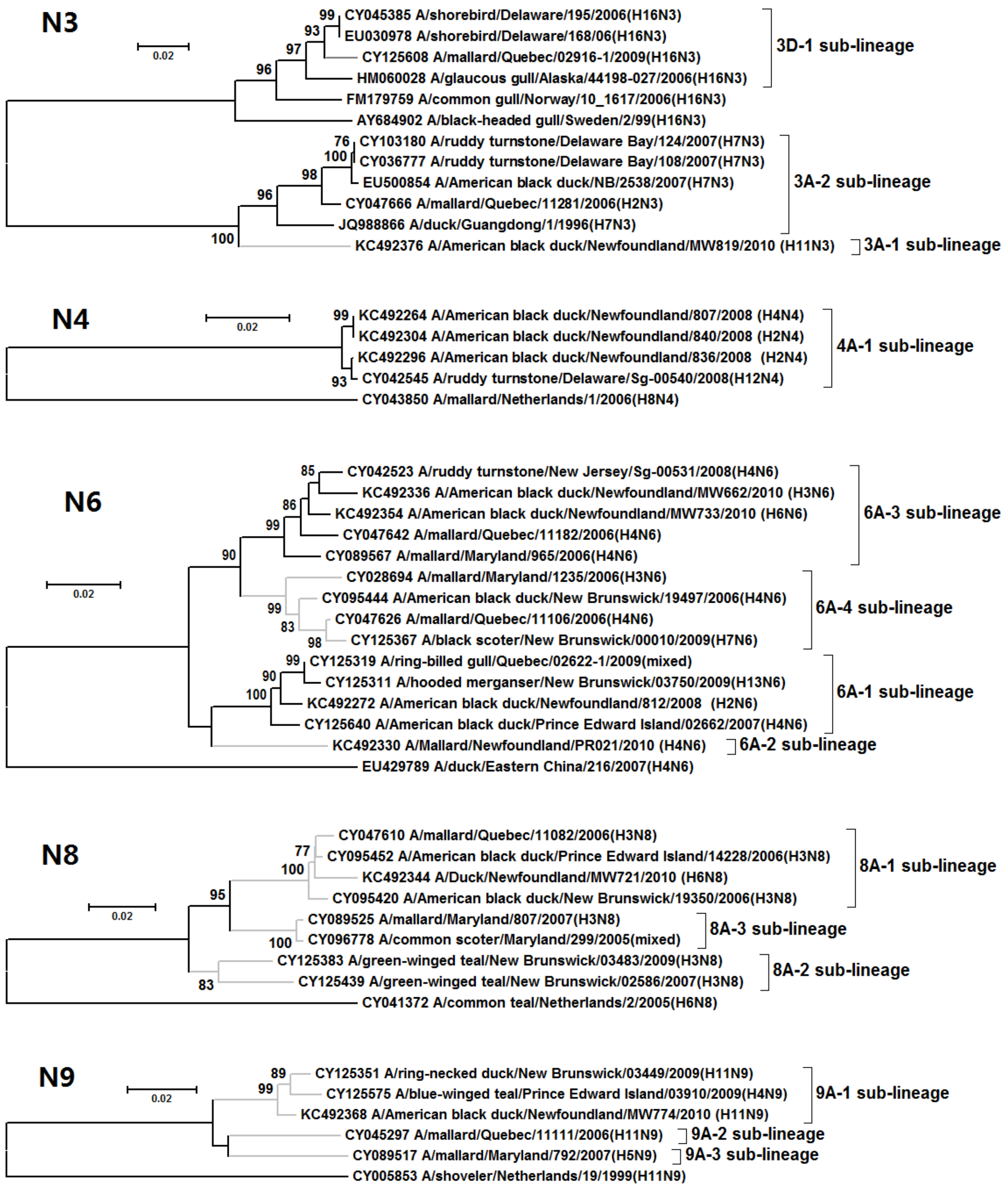
N3
0.02
99 CY045385 A/shorebird/Delaware/195/2006(H16N3)
93 EU030978 A/shorebird/Delaware/168/06(H16N3)
97 CY125608 A/mallard/Quebec/02916-1/2009(H16N3)
96 HM060028 A/glaucous gull/Alaska/44198-027/2006(H16N3)
FM179759 A/common gull/Norway/10_1617/2006(H16N3)
AY684902 A/black-headed gull/Sweden/2/99(H16N3)
76 CY103180 A/ruddy turnstone/Delaware Bay/124/2007(H7N3)
100 CY036777 A/ruddy turnstone/Delaware Bay/108/2007(H7N3)
98 EU500854 A/American black duck/NB/2538/2007(H7N3)
96 CY047666 A/mallard/Quebec/11281/2006(H2N3)
JQ988866 A/duck/Guangdong/1/1996(H7N3)
100 KC492376 A/American black duck/Newfoundland/MW819/2010 (H11N3)
3D-1 sub-lineage
3A-2 sub-lineage
3A-1 sub-lineage
N4
0.02
99 KC492264 A/American black duck/Newfoundland/807/2008 (H4N4)
KC492304 A/American black duck/Newfoundland/840/2008 (H2N4)
KC492296 A/American black duck/Newfoundland/836/2008 (H2N4)
93 CY042545 A/ruddy turnstone/Delaware/Sg-00540/2008(H12N4)
CY043850 A/mallard/Netherlands/1/2006(H8N4)
4A-1 sub-lineage
N6
0.02
85 CY042523 A/ruddy turnstone/New Jersey/Sg-00531/2008(H4N6)
KC492336 A/American black duck/Newfoundland/MW662/2010 (H3N6)
86 KC492354 A/American black duck/Newfoundland/MW733/2010 (H6N6)
99 CY047642 A/mallard/Quebec/11182/2006(H4N6)
90 CY089567 A/mallard/Maryland/965/2006(H4N6)
CY028694 A/mallard/Maryland/1235/2006(H3N6)
99 CY095444 A/American black duck/New Brunswick/19497/2006(H4N6)
83 CY047626 A/mallard/Quebec/11106/2006(H4N6)
98 CY125367 A/black scoter/New Brunswick/00010/2009(H7N6)
99 CY125319 A/ring-billed gull/Quebec/02622-1/2009(mixed)
90 CY125311 A/hooded merganser/New Brunswick/03750/2009(H13N6)
100 KC492272 A/American black duck/Newfoundland/812/2008 (H2N6)
CY125640 A/American black duck/Prince Edward Island/02662/2007(H4N6)
KC492330 A/Mallard/Newfoundland/PR021/2010 (H4N6)
EU429789 A/duck/Eastern China/216/2007(H4N6)
6A-3 sub-lineage
6A-4 sub-lineage
6A-1 sub-lineage
6A-2 sub-lineage
N8
0.02
CY047610 A/mallard/Quebec/11082/2006(H3N8)
77 CY095452 A/American black duck/Prince Edward Island/14228/2006(H3N8)
100 KC492344 A/Duck/Newfoundland/MW721/2010 (H6N8)
95 CY095420 A/American black duck/New Brunswick/19350/2006(H3N8)
CY089525 A/mallard/Maryland/807/2007(H3N8)
100 CY096778 A/common scoter/Maryland/299/2005(mixed)
CY125383 A/green-winged teal/New Brunswick/03483/2009(H3N8)
83 CY125439 A/green-winged teal/New Brunswick/02586/2007(H3N8)
CY041372 A/common teal/Netherlands/2/2005(H6N8)
8A-1 sub-lineage
8A-3 sub-lineage
8A-2 sub-lineage
N9
0.02
89 CY125351 A/ring-necked duck/New Brunswick/03449/2009(H11N9)
99 CY125575 A/blue-winged teal/Prince Edward Island/03910/2009(H4N9)
KC492368 A/American black duck/Newfoundland/MW774/2010 (H11N9)
CY045297 A/mallard/Quebec/11111/2006(H11N9)
CY089517 A/mallard/Maryland/792/2007(H5N9)
CY005853 A/shoveler/Netherlands/19/1999(H11N9)
9A-1 sub-lineage
9A-2 sub-lineage
9A-3 sub-lineage
