# Supplementary material for: Genetic Structure of Avian Influenza Viruses from Ducks of the Atlantic Flyway of North America
Source: PLoS One. 2014 Jan 30;9(1):e86999. doi: 10.1371/journal.pone.0086999 (PMC3907406; doi:10.1371/journal.pone.0086999)
Supplement: Figure S3 — Gene type categorization of the Atlantic flyway duck AIVs by year and location. Gene typing was done as described in the Materials and Methods section such that sequences with nucleotide identity of ≥99% are considered as homologous genes or the same gene type. Sequences from different locations within the Atlantic bird flyway are shown in different colors, as indicated in the legend. The locations are abbreviated as follows: NL, Newfoundland; QC, Quebec; MD, Maryland; NB, New Brunswick; PEI, Prince Edward Island; NY, New York; PA, Pennsylvania; DE, Delaware; ON, Ontario; FL, Florida; NS, Nova Scotia. (PDF) [file pone.0086999.s003.pdf]

Figure S3. Gene type categorization of the Atlantic flyway duck AIVs by year and location. Gene typing was done as described in the Materials and Methods section such that sequences with nucleotide identity of ≥99% are considered as homologous genes or the same gene type. Sequences from different locations within the Atlantic bird flyway are shown in different colours, as indicated in the legend. The locations are abbreviated as follows: NL, Newfoundland; QC, Quebec; MD, Maryland; NB, New Brunswick; PEI, Prince Edward Island; NY, New York; PA, Pennsylvania; DE, Delaware; ON, Ontario; FL, Florida; NS, Nova Scotia.

PB2

2006		2007		2008	2009	2010	2011
C-2.6	C-2.7	C-2.6	C-2.7	C-1.1	C-2.1	C-2.1	C-2.2
C-2.7	C-2.13	C-2.6	C-2.7	C-1.1	C-2.1	C-2.1	C-2.2
C-2.7	C-2.14	C-2.6	C-2.7	C-1.1	C-2.1	C-2.1	C-2.2
C-2.11	C-2.6	C-2.6	C-4.1	C-1.1	C-2.4	C-2.3	C-2.2
C-2.12	C-2.6	C-2.6	C-4.1	C-1.1	C-2.18	C-2.3	C-2.2
C-2.12	C-2.6	C-2.6	C-2.10	C-1.1	C-2.18	C-2.3	C-2.2
C-2.12	C-2.7	C-2.6	C-2.10	C-2.2	C-3.5	C-2.4	C-2.5
C-2.12	C-2.7	C-2.6	C-3.2	C-2.2	C-3.5	C-2.4	C-3.1
C-2.12	C-2.10	C-2.6	C-3.3		C-3.5	C-2.5	
C-2.12	C-2.9	C-2.6			J-1.2		
C-2.12	C-4.1	C-2.6			C-2.2		
C-2.12	C-4.1	C-2.7			C-2.2		
C-2.12	C-2.12	C-2.19			C-2.8		
C-3.2	C-2.12	C-2.19			C-3.5		
C-3.2	C-2.15	C-2.19			C-3.5		
C-3.2	C-5.1	C-2.19			C-3.5		
C-3.3	C-2.17	C-2.19			C-3.5		
C-3.3	C-2.16	C-2.19			J-1.1		
C-3.4		C-4.1					

Legend	
NL	DE
QC	MD
NB	FL
NS	PA
PEI	NY
ON	

PB1

2006		2007		2008	2009	2010	2011
F-1.3	F-3.2	F-3.5	F-5.2	F-3.2	F-3.1	F-1.1	F-1.1
F-1.3	F-5.1	F-3.5	F-5.2	F-3.2	F-3.1	F-1.1	F-2.1
F-2.2	F-6.2	F-3.5	F-7.1	F-3.2	F-3.1	F-1.2	F-2.1
F-3.4	F-3.3	F-3.5	F-3.6	F-3.2	F-3.1	F-1.2	F-2.1
F-3.4	F-3.5	F-3.5	F-3.6	F-3.2	F-1.2	F-3.1	F-4.1
F-3.4	F-3.5	F-3.5	F-3.2	F-3.2	F-3.6	F-3.1	F-4.1
F-3.4	F-5.1	F-3.5	F-3.2	F-4.2	F-4.5	F-3.1	F-4.1
F-3.4	F-5.1	F-3.5	F-4.7	F-4.2	F-4.5	F-3.1	F-4.4
F-3.4	F-5.1	F-3.5	F-3.8		F-4.5	F-4.3	
F-3.4	F-3.2	F-3.5			F-8.1		
F-3.4	F-3.6	F-3.6			F-2.1		
F-3.4	F-3.6	F-3.6			F-3.11		
F-3.7	F-1.4	F-3.6			F-3.12		
F-3.7	F-1.4	F-3.6			F-3.12		
F-3.8	F-5.1	F-3.6			F-4.5		
F-4.6	F-3.10	F-3.6			F-4.5		
F-4.6	F-3.9	F-3.6			F-4.5		
F-4.6	F-1.4	F-4.5			F-8.1		
F-4.6		F-6.1					

PA

2006		2007		2008	2009	2010	2011
E-3.1	E-2.2	E-1.3	H-1.12	E-2.1	E-1.1	E-1.1	E-1.2
E-3.3	H-1.10	E-1.4	H-1.13	E-2.1	E-1.1	E-1.1	E-2.1

E-5.1	H-1.11	H-1.17	H-1.13	E-2.1	E-1.1	H-1.4	E-2.1
E-5.1	E-3.2	H-1.17	H-1.1	H-1.1	H-1.3	H-1.4	E-2.1
E-5.1	E-3.1	H-1.17	E-3.1	H-1.1	E-2.1	H-1.4	E-2.1
E-5.2	E-3.2	H-1.17	H-1.6	H-1.1	E-2.1	H-1.4	E-2.1
E-5.2	E-4.1	H-1.17	H-1.6	H-1.1	H-1.17	H-1.5	H-1.2
E-5.2	E-4.1	H-1.17	H-1.14	H-1.1	E-2.1	H-1.5	H-1.5
H-1.7	E-4.1	E-3.1	E-3.4		E-2.1	H-1.5	
H-1.7	H-1.1	E-4.1			E-6.2		
H-1.7	H-1.1	E-4.1			E-2.1		
H-1.7	H-1.1	E-4.1			E-2.1		
H-1.7	E-1.5	E-4.1			E-2.1		
H-1.7	E-1.5	E-4.1			E-6.1		
H-1.7	E-5.1	E-4.1			H-1.15		
H-1.7	H-1.8	E-4.1			H-1.15		
H-1.7	E-3.5	E-4.1			H-1.16		
H-1.7	E-1.5	E-4.1			H-1.17		
H-1.9		E-4.1					

HA

2006		2007		2008	2009	2010	2011
2H-2.1	3C-2.2	3C-1.2	3C-1.3	2H-1.2	1D-1.1	3D-1.1	2H-1.1
2H-2.1	4A-1.3	3C-1.2	5C-1.2	2H-1.2	1D-1.1	4A-1.2	2H-1.1
2H-2.1	5C-1.2	3C-1.2	5C-1.2	2H-1.3	1D-1.1	4A-2.1	3C-1.1
3C-1.2	3C-1.2	3C-1.2	4A-1.5	3D-1.1	5C-1.1	4A-2.1	3C-1.1
3C-2.1	4A-1.4	3C-1.2	4A-1.5	3D-1.1	3D-1.2	6B-1.1	3C-1.1
3C-2.1	4A-1.6	3C-1.2	3D-1.1	4A-1.1	3D-2.1	6B-1.1	3C-1.1
3C-2.1	4A-1.6	3C-1.2	3D-1.1	4A-1.1	4A-3.2	6B-1.1	3C-1.1
3C-2.1	4A-1.6	3C-1.2	5C-1.4	4A-1.1	3D-1.2	11C-1.1	12A-1.1
3C-2.1	4A-1.6	3C-1.2	5C-1.2		3D-1.2	11C-1.1	
3C-2.1	3C-1.2	3C-1.2			16D-1.1		
3C-2.1	3C-1.2	3C-2.3			3C-2.3		
3C-2.1	4A-1.5	3D-2.1			3D-1.2		
3C-2.1	5C-1.2	3D-2.1			3D-1.2		
4A-1.4	5C-1.3	3D-2.1			3D-1.2		
4A-1.4	5C-1.3	3D-2.1			3D-1.3		
4A-1.4	7F-2.1	3D-2.1			4A-3.1		
4A-1.4	5C-1.5	3D-2.1			11C-1.2		
4A-1.4	5C-1.3	4A-1.5			13A-1.1		
11C-2.1		7F-1.1					

NP

2006		2007		2008	2009	2010	2011
H-1.2	H-2.4	H-1.5	H-1.7	H-1.2	H-4.1	H-1.1	H-1.4
H-1.5	H-3.4	H-1.5	H-1.7	H-1.2	H-4.1	H-1.1	H-2.1
H-1.5	H-5.2	H-1.8	F-1.1	H-1.2	H-4.1	H-1.1	H-2.1
H-1.5	H-1.8	H-1.8	H-1.5	H-3.1	H-4.2	H-1.1	H-2.1
H-1.5	H-2.3	H-1.8	H-4.3	H-3.1	H-1.6	H-1.3	H-2.1
H-1.5	H-6.1	H-1.8	H-2.3	H-3.1	H-1.6	H-1.3	H-2.1

H-2.3	H-6.1	H-1.8	H-3.2	H-3.1	H-4.6	H-2.1	H-5.1
H-3.3	H-6.1	H-1.8	H-1.6	H-3.1	H-4.6	H-2.2	H-5.1
H-3.3	H-6.2	H-1.8	H-6.1		H-4.6	H-2.2	
H-4.5	H-1.2	H-1.8			D-1.1		
H-6.2	H-1.2	H-1.8			H-1.1		
H-6.2	H-4.3	H-1.8			H-1.1		
H-6.2	H-4.3	H-2.4			H-1.9		
H-6.2	H-4.4	H-4.7			H-4.5		
H-6.2	H-4.4	H-4.7			H-4.6		
H-6.2	H-7.1	H-4.7			H-4.6		
H-6.2	H-2.5	H-4.7			H-4.6		
H-6.2	H-4.4	H-4.7			D-1.1		
H-6.2		H-4.7					

NA

2006		2007		2008	2009	2010	2011
2D-2.1	2D-1.1	3A-2.1	1E- 2.1	4A-1.1	1E-1.1	3A-1.1	2D
2D-2.1	6A-3.3	6A-1.3	8A-3.1	4A-1.1	1E-1.1	6A-2.1	2D
2D-2.1	6A-4.3	6A-4.2	9A-3.1	4A-1.1	1E-1.1	6A-2.1	2D
2D-2.1	6A-1.1	8A-1.1	6A-1.3	6A-1.1	4A	6A-2.1	2D
2D-2.1	6A-4.1	8A-1.1	6A-1.3	6A-1.1	8A-2.1	6A-2.1	2D
2D-2.1	6A-4.1	8A-1.1	2D-1.1	6A-1.1	8A-2.2	6A-3.1	2D-1.1
2D-2.1	8A-1.1	8A-1.1	2D-1.1	8A	9A-1.2	6A-3.2	2D-1.2
2D-2.1	8A-1.1	8A-1.1	1E- 2.2	8A-2.1	8A-2.1	8A-1.3	-
2D-2.1	8A-1.1	8A-1.1	2G-1.2		8A-2.1	9A-1.1	
3A-2.2	6A-1.2	8A-1.1			3D-1.1		
3A-2.2	8A-1.2	8A-1.1			2D-1.1		
3A-2.2	8A-1.2	8A-1.1			6A-1.2		
6A-3.4	2D-1.3	8A-1.1			6A-3.2		
6A-4.2	2D-1.3	8A-2.2			8A-2.1		
6A-4.2	2D-3.1	8A-2.2			8A-2.1		
6A-4.2	2G-1.1	8A-2.2			8A-2.1		
6A-4.2	2D-1.3	8A-2.2			8A-2.2		
8A-1.4	2D-1.3	8A-2.2			9A-1.3		
9A-2.1		8A-2.2					

M

2006		2007		2008	2009	2010	2011
E-1.3	E-1.3	E-1.5	E-1.9	E-1.3	E-1.1	E-1.1	E-1.2
E-1.3	E-1.4	E-1.8	E-1.5	E-1.3	E-1.1	E-1.1	E-1.2
E-1.3	E-1.15	E-1.10	E-1.5	E-1.3	E-1.1	E-1.1	E-1.2
E-1.3	E-1.3	E-1.10	E-1.8	E-1.3	E-1.7	E-1.1	E-1.2
E-1.3	E-1.3	E-1.10	E-1.11	E-1.3	E-1.3	E-1.5	E-1.2
E-1.3	E-1.3	E-1.10	E-1.12	E-1.3	E-1.18	E-1.5	E-1.2
E-1.3	E-1.8	E-1.10	E-1.12	E-1.3	E-1.19	E-1.5	E-1.6
E-1.3	E-1.10	E-1.10	E-1.16	E-1.3	E-1.19	E-1.6	E-1.7
E-1.3	E-1.10	E-1.10	E-1.16		E-1.19	E-1.6	
E-1.3	E-1.10	E-1.10			J-1.1		

E-1.3	E-1.10	E-1.10			E-1.5		
E-1.3	E-1.11	E-1.10			E-1.17		
E-1.3	E-2.1	E-1.10			E-1.19		
E-1.3	E-1.3	E-1.18			E-1.19		
E-1.13	E-1.3	E-1.18			E-1.19		
E-1.13	E-1.5	E-1.18			E-1.20		
E-1.13	E-1.16	E-1.18			E-1.21		
E-1.14	E-1.3	E-1.18			J-1.1		
E-1.14		E-1.18					

NS

2006		2007		2008	2009	2010	2011
2B-1.3	2B-1.5	2B-1.6	2B-1.4	1D-1.1	2B-1.1	1D-1.2	2B-1.2
1D-1.7	1D-1.10	2B-1.6	1D-1.7	1D-1.1	2B-1.1	1D-1.4	2B-1.2
1D-1.7	1D-1.12	2B-1.6	1D-1.7	1D-1.1	2B-1.2	1D-1.4	2B-1.2
1D-1.7	2B-1.6	2B-1.6	1D-1.6	1D-1.1	1D-1.5	1D-1.5	2B-1.2
1D-1.7	2B-1.6	2B-1.6	1D-1.7	1D-1.1	1D-1.13	1D-1.5	2B-1.2
1D-1.7	2B-1.6	2B-1.6	1D-1.1	1D-1.1	1D-1.13	1D-1.5	2B-1.2
1D-1.7	1D-1.7	2B-1.6	1D-1.1	1D-1.1	1D-1.13	1D-1.5	2B-1.3
1D-1.7	1D-1.7	2B-1.6	1D-1.7	1D-1.1	1D-1.13	1D-1.5	1D-1.3
1D-1.7	1D-1.8	2B-1.6	1D-1.7		1D-1.13	1D-1.5	
1D-1.7	2B-1.6	2B-1.6			1C-1.1		
1D-1.7	2B-1.6	1D-1.1			2B-1.7		
1D-1.9	1D-1.6	1D-1.1			2B-1.8		
1D-1.9	2B-2.1	1D-1.1			1D-1.7		
1D-1.9	1D-1.1	1D-1.1			1D-1.13		
1D-1.9	1D-1.1	1D-1.1			1D-1.13		
1D-1.9	1D-1.7	1D-1.6			1D-1.13		
1D-1.10	1D-1.10	1D-1.6			1D-1.13		
1D-1.10	1D-1.1	1D-1.9			1C-1.1		
1D-1.11		1D-1.9					
