# Supplementary material for: Genetic Structure of Avian Influenza Viruses from Ducks of the Atlantic Flyway of North America
Source: PLoS One. 2014 Jan 30;9(1):e86999. doi: 10.1371/journal.pone.0086999 (PMC3907406; doi:10.1371/journal.pone.0086999)
Supplement: Figure S4 — Distribution of the AIV gene types from Atlantic flyway ducks by location. Gene typing was done as described in the Materials and Methods section such that sequences with nucleotide identity of ≥99% are considered as homologous genes or the same gene type. Sequences from different locations within the Atlantic bird flyway are shown in different colors, as indicated in the legend. The locations are abbreviated as follows: NL, Newfoundland; QC, Quebec; MD, Maryland; NB, New Brunswick; PEI, Prince Edward Island; NY, New York; PA, Pennsylvania; DE, Delaware; ON, Ontario; FL, Florida; NS, Nova Scotia. The 18 gene types detected only in Newfoundland are labelled with asterisk (*). (PDF) [file pone.0086999.s004.pdf]

Figure S4. Distribution of the AIV gene types from Atlantic flyway ducks by location. Gene typing was done as described in the Materials and Methods section such that sequences with nucleotide identity of ≥99% are considered as homologous genes or the same gene type. Sequences from different locations within the Atlantic bird flyway are shown in different colours, as indicated in the legend. The locations are abbreviated as follows: NL, Newfoundland; QC, Quebec; MD, Maryland; NB, New Brunswick; PEI, Prince Edward Island; NY, New York; PA, Pennsylvania; DE, Delaware; ON, Ontario; FL, Florida; NS, Nova Scotia. The 18 gene types detected only in Newfoundland are labeled with asterisk (*).

	NL	QC	NB	PEI	NS	MD	NY	DE	PA	ON	FL
PB2											
C-1.1	C-1.1*										
C-2.1	C-2.1										
C-2.2	C-2.2		C-2.2		C-2.2						
C-2.3	C-2.3										
C-2.4	C-2.4*										
C-2.5	C-2.5										
C-2.6		C-2.6	C-2.6								
C-2.7		C-2.7	C-2.7			C-2.7					
C-2.8			C-2.8								
C-2.9				C-2.9							
C-2.10	C-2.10		C-2.10								
C-2.11		C-2.11									
C-2.12		C-2.12					C-2.12				
C-2.13						C-2.13					
C-2.14						C-2.14					
C-2.15							C-2.15				
C-2.16									C-2.16		
C-2.17								C-2.17			
C-2.18				C-2.18							
C-2.19			C-2.19								
C-3.1	C-3.1										
C-3.2		C-3.2								C-3.2	
C-3.3		C-3.3									C-3.3
C-3.4		C-3.4									
C-3.5			C-3.5	C-3.5	C-3.5						
C-4.1			C-4.1	C-4.1							
C-5.1							C-5.1				
J-1.1			J-1.1								
J-1.2		J-1.2									
PB1											
F-1.1	F-1.1										
F-1.2	F-1.2			F-1.2							
F-1.3		F-1.3									
F-1.4							F-1.4		F-1.4		
F-2.1	F-2.1		F-2.1								
F-2.2		F-2.2*									
F-3.1	F-3.1										
F-3.2	F-3.2			F-3.2		F-3.2					
F-3.3			F-3.3								
F-3.4		F-3.4									
F-3.5			F-3.5								

F-3.6			F-3.6	F-3.6							
F-3.7		F-3.7									
F-3.8		F-3.8									F-3.8
F-3.9								F-3.9			
F-3.10							F-3.10				
F-3.11					F-3.11						
F-3.12			F-3.12								
F-4.1	F-4.1										
F-4.2	F-4.2										
F-4.3	F-4.3										
F-4.4	F-4.4										
F-4.5			F-4.5	F-4.5	F-4.5						
F-4.6		F-4.6									
F-4.7										F-4.7	
F-5.1			F-5.1			F-5.1	F-5.1				
F-5.2						F-5.2					
F-6.1			F-6.1								
F-6.2						F-6.2					
F-7.1						F-7.1					
F-8.1		F-8.1	F-8.1								
PA											
H-1.1	H-1.1			H-1.1							
H-1.2	H-1.2										
H-1.3	H-1.3										
H-1.4	H-1.4										
H-1.5	H-1.5										
H-1.6	H-1.6										
H-1.7		H-1.7									
H-1.8							H-1.8				
H-1.9		H-1.9									
H-1.10						H-1.10					
H-1.11						H-1.11					
H-1.12						H-1.12					
H-1.13						H-1.13					
H-1.14										H-1.14	
H-1.15			H-1.15								
H-1.16			H-1.16								
H-1.17				H-1.17	H-1.17						
E-1.1	E-1.1										
E-1.2	E-1.2										
E-1.3			E-1.3								
E-1.4			E-1.4								
E-1.5							E-1.5		E-1.5		
E-2.1	E-2.1		E-2.1	E-2.1	E-2.1						

E-2.2						E-2.2					
E-3.1		E-3.1	E-3.1	E-3.1							
E-3.2			E-3.2								
E-3.3		E-3.3									
E-3.4											E-3.4
E-3.5								E-3.5			
E-4.1			E-4.1								
E-5.1		E-5.1					E-5.1				
E-5.2		E-5.2									
E-6.1			E-6.1								
E-6.2		E-6.2									
HA											
1D-1.1	1D-1.1										
2H-1.1	2H-1.1										
2H-1.2	2H-1.2*										
2H-1.3	2H-1.3										
2H-2.1		2H-2.1									
3C-1.1	3C-1.1										
3C-1.2		3C-1.2	3C-1.2	3C-1.2							
3D-1.3					3D-1.3	3C-1.3					
3C-2.1		3C-2.1*									
3C-2.2						3C-2.2					
3C-2.3			3C-2.3*								
3D-1.1	3D-1.1										
3D-1.2			3D-1.2	3D-1.2	3D-1.2						
3D-1.3					3D-1.3						
3D-2.1			3D-2.1	3D-2.1							
4A-1.1	4A-1.1*										
4A-1.2	4A-1.2*										
4A-1.3						4A-1.3					
4A-1.4		4A-1.4	4A-1.4								
4A-1.5			4A-1.5	4A-1.5							
4A-1.6			4A-1.6*								
4A-2.1	4A-2.1										
4A-3.1			4A-3.1								
4A-3.2				4A-3.2							
5C-1.1	5C-1.1*										
5C-1.2						5C-1.2	5C-1.2				5C-1.2
5C-1.3							5C-1.3		5C-1.3		
5C-1.4										5C-1.4	
5C-1.5								5C-1.5			
6B-1.1	6B-1.1										
7F-1.1			7F-1.1*								
7F-2.1							7F-2.1				

11C-1.1	11C-1.1*										
11C-1.2			11C-1.2								
11C-2.1		11C-2.1									
12A-1.1	12A-1.1*										
13A-1.1			13A-1.1								
16D-1.1		16D-1.1*									
NP											
F-1.1						F-1.1					
H-1.1	H-1.1		H-1.1								
H-1.2	H-1.2	H-1.2		H-1.2							
H-1.3	H-1.3*										
H-1.4	H-1.4										
H-1.5		H-1.5	H-1.5	H-1.5							
H-1.6				H-1.6						H-1.6	
H-1.7						H-1.7					
H-1.8			H-1.8*								
H-1.9			H-1.9								
H-2.1	H-2.1										
H-2.2	H-2.2										
H-2.3	H-2.3	H-2.3	H-2.3								
H-2.4			H-2.4			H-2.4					
H-2.5								H-2.5			
H-3.1	H-3.1										
H-3.2	H-3.2										
H-3.3		H-3.3									
H-3.4						H-3.4					
H-4.1	H-4.1										
H-4.2	H-4.2										
H-4.3				H-4.3			H-4.3				
H-4.4							H-4.4		H-4.4		
H-4.5		H-4.5			H-4.5						
H-4.6			H-4.6	H-4.6	H-4.6						
H-4.7			H-4.7								
H-5.1	H-5.1										
H-5.2						H-5.2					
H-6.1			H-6.1								H-6.1
H-6.2		H-6.2	H-6.2								
H-7.1							H-7.1				
D-1.1			D-1.1								
D-1.2		D-1.1									
NA											
1E-1.1	1E-1.1										
1E-2.1						1E-2.1					

1E-2.2										1E-2.2	
2D-1.1	2D-1.1					2D-1.1					
2D-1.2	2D-1.2*										
2D-1.3							2D-1.3	2D-1.3	2D-1.3		
2D-2.1		2D-2.1									
2D-3.1							2D-3.1				
2G-1.1							2G-1.1				
2G-1.2											2G-1.2
3A-1.1	3A-1.1										
3A-2.1			3A-2.1								
3A-2.2		3A-2.2									
3D-1.1		3D-1.1									
4A-1.1	4A-1.1										
6A-1.1	6A-1.1		6A-1.1								
6A-1.2			6A-1.2	6A-1.2							
6A-1.3			6A-1.3	6A-1.3							
6A-2.1	6A-2.1										
6A-3.1	6A-3.1										
6A-3.2	6A-3.2		6A-3.2								
6A-3.3						6A-3.3					
6A-3.4		6A-3.4									
6A-4.1			6A-4.1								
6A-4.2		6A-4.2	6A-4.2								
6A-4.3						6A-4.3					
8A-1.1			8A-1.1								
8A-1.2				8A-1.2							
8A-1.3	8A-1.3										
8A-1.4		8A-1.4									
8A-2.1	8A-2.1		8A-2.1	8A-2.1	8A-2.1						
8A-2.2			8A-2.2	8A-2.2	8A-2.2						
8A-3.1						8A-3.1					
9A-1.1	9A-1.1										
9A-1.2				9A-1.2							
9A-1.3			9A-1.3								
9A-2.1		9A-2.1									
9A-3.1						9A-3.1					
M											
E-1.1	E-1.1										
E-1.2	E-1.2										
E-1.3	E-1.3	E-1.3	E-1.3	E-1.3		E-1.3	E-1.3		E-1.3		
E-1.4						E-1.4					
E-1.5	E-1.5		E-1.5			E-1.5	E-1.5				
E-1.6	E-1.6										
E-1.7	E-1.7										

E-1.8			E-1.8	E-1.8							
E-1.9						E-1.9					
E-1.10			E-1.10	E-1.10							
E-1.11				E-1.11							
E-l.12	E-l.12										
E-1.13		E-1.13									
E-1.14		E-1.14									
E-1.15						E-1.15					
E-1.16									E-1.16	E-1.16	E-1.16
E-1.17			E-1.17								
E-1.18			E-1.18	E-1.18	E-1.19						
E-1.19			E-1.19	E-1.19							
E-1.20			E-1.20								
E-1.21					E-1.21						
E-2.1							E-2.1				
J-1.1		J-1.1	J-1.1								
NS											
1D-1.1	1D-1.1		1D-1.1				1D-1.1		1D-1.1		
1D-1.2	1D-1.2										
1D-1.3	1D-1.3										
1D-1.4	1D-1.4										
1D-1.5	1D-1.5										
1D-1.6			1D-1.6	1D-1.6							
1D-1.7		1D-1.7	1D-1.7	1D-1.7		1D-1.7	1D-1.7			1D-1.7	1D-1.7
1D-1.8			1D-1.8								
1D-1.9		1D-1.9	1D-1.9								
1D-1.10		1D-1.10				1D-1.10		1D-1.10			
1D-1.11		1D-1.11									
1D-1.12						1D-1.12					
1D-1.13			1D-1.13	1D-1.13	1D-1.13						
1C-1.1			1C-1.1								
2B-1.1	2B-1.1										
2B-1.2	2B-1.2										
2B-1.3	2B-1.3	2B-1.3									
2B-1.4						2B-1.4					
2B-1.5						2B-1.5					
2B-1.6			2B-1.6	2B-1.6							
2B-1.7			2B-1.7								
2B-1.8			2B-1.8								
2B-2.1							2B-2.1				
