# Supplementary material for: Genetic Structure of Avian Influenza Viruses from Ducks of the Atlantic Flyway of North America
Source: PLoS One. 2014 Jan 30;9(1):e86999. doi: 10.1371/journal.pone.0086999 (PMC3907406; doi:10.1371/journal.pone.0086999)
Supplement: Figure S5 — Spatial distribution of detections of the Atlantic Flyway duck AIV gene sub-lineages in North American flyways. The numbers of viruses identified in each flyway are shown for the indicated gene sub-lineages that were identified in the 2006-2011 Atlantic flyway duck viruses. Only sub-lineages with ≥10 genes detected in at least one flyway are shown. Flyways are shown in different shading: Atlantic flyway (AF), black; Mississippi flyway (MF), dark grey; Central flyway (CF), light grey; Pacific flyway (PF), white. (PDF) [file pone.0086999.s005.pdf]

Figure S5. Spatial distribution of detections of the Atlantic flyway duck AIV gene sub-lineages in North American flyways. The numbers of viruses identified in each flyway are shown for the indicated gene sub-lineages that were identified in the 2006-2011 Atlantic flyway duck viruses. Only sub-lineages with $\geq$10 genes detected in at least one flyway are shown. Flyways are shown in different shading: Atlantic flyway (AF), black; Mississippi flyway (MF), dark grey; Central flyway (CF), light grey; Pacific flyway (PF), white.

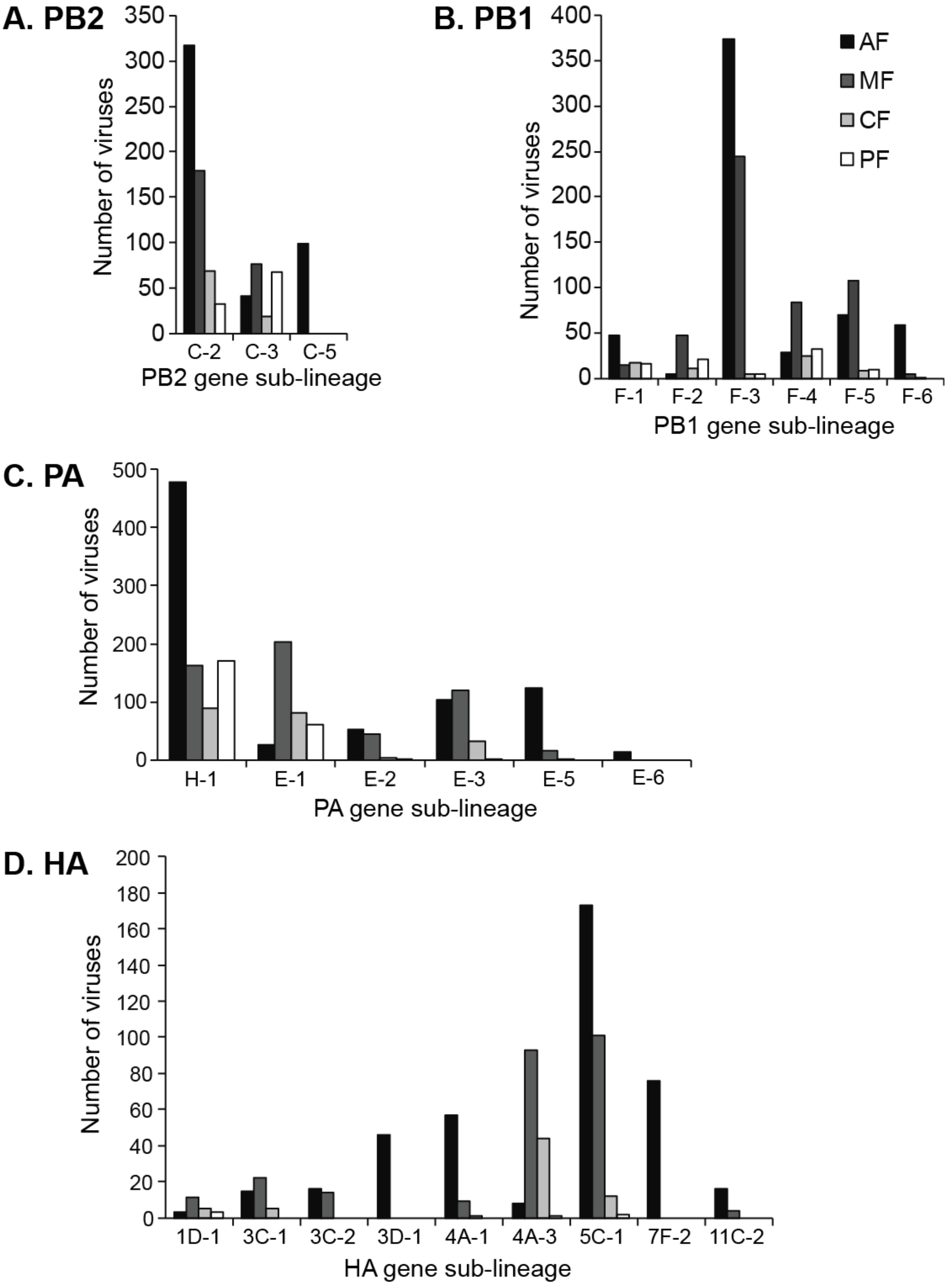

A. PB2
350
300
250
200
150
100
50
0
Number of viruses
C-2
C-3
C-5
PB2 gene sub-lineage
B. PB1
400
350
300
250
200
150
100
50
0
Number of viruses
AF
MF
CF
PF
F-1
F-2
F-3
F-4
F-5
F-6
PB1 gene sub-lineage
C. PA
500
400
300
200
100
0
Number of viruses
H-1
E-1
E-2
E-3
E-5
E-6
PA gene sub-lineage
D. HA
200
180
160
140
120
100
80
60
40
20
0
Number of viruses
1D-1
3C-1
3C-2
3D-1
4A-1
4A-3
5C-1
7F-2
11C-2
HA gene sub-lineage

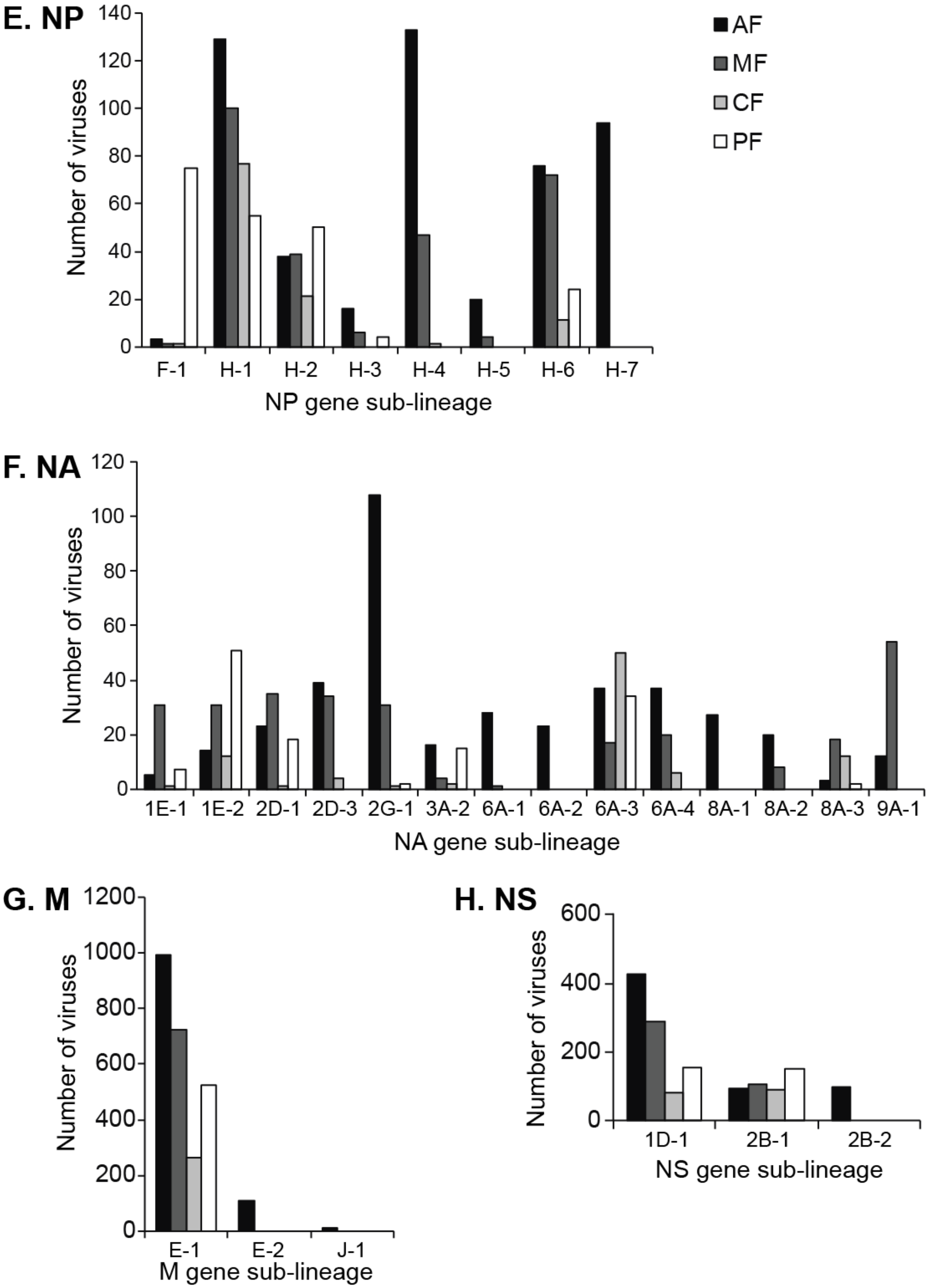
E. NP
AF
MF
CF
PF
Number of viruses
0
20
40
60
80
100
120
140
F-1
H-1
H-2
H-3
H-4
H-5
H-6
H-7
NP gene sub-lineage
F. NA
Number of viruses
0
20
40
60
80
100
120
1E-1
1E-2
2D-1
2D-3
2G-1
3A-2
6A-1
6A-2
6A-3
6A-4
8A-1
8A-2
8A-3
9A-1
NA gene sub-lineage
G. M
Number of viruses
0
200
400
600
800
1000
1200
E-1
E-2
J-1
M gene sub-lineage
H. NS
Number of viruses
0
200
400
600
1D-1
2B-1
2B-2
NS gene sub-lineage
