# Supplementary material for: Genetic Structure of Avian Influenza Viruses from Ducks of the Atlantic Flyway of North America
Source: PLoS One. 2014 Jan 30;9(1):e86999. doi: 10.1371/journal.pone.0086999 (PMC3907406; doi:10.1371/journal.pone.0086999)
Supplement: Table S1 — The 25 duck AIVs from Newfoundland, Canada, sequenced in this study. (PDF) [file pone.0086999.s006.pdf]

Table S1. The 25 duck AIVs from Newfoundland, Canada, sequenced in this study.

HA	Virus	Abbreviation	Date (mm/dd/yy)	Site
H2	A/Northern pintail/Newfoundland/GR683/2011(H2N2)	NOPI NL GR683 2011	10/13/11	Commonwealth Pond
	A/Mallard/Newfoundland/GR475/2011(H2N2)	MALL NL GR475 2011	09/14/11	Quidi Vidi Lake
	A/American black duck/Newfoundland/836/2008(H2N4)	ABDU NL 836 2008	10/08/08	Mundy Pond
	A/American black duck/Newfoundland/840/2008(H2N4)	ABDU NL 840 2008	10/15/08	Mundy Pond
	A/American black duck/Newfoundland/812/2008(H2N6)	ABDU NL 812 2008	09/26/08	Mundy Pond
H3	A/American black duck/Newfoundland/GR252/2011(H3N2)	ABDU NL GR252 2011	08/31/11	Mundy Pond
	A/American black duck/Newfoundland/GR490/2011(H3N2)	ABDU NL GR490 2011	09/17/11	Commonwealth Pond
	A/American black duck/Newfoundland/GR256/2011(H3N2)	ABDU NL GR256 2011	08/31/11	Mundy Pond
	A/American black duck/Newfoundland/GR396/2011(H3N2)	ABDU NL GR396 2011	0912/11	Commonwealth Pond
	A/American black duck/Newfoundland/MW662/2010(H3N6)	ABDU NL MW662 2010	09/17/10	Commonwealth Pond
	A/American black duck/Newfoundland/732/2008(H3N8)	ABDU NL 732 2008	09/15/08	Mundy Pond
	A/American black duck/Newfoundland/734/2008 H3N8)	ABDU NL 734 2008	09/19/08	Mundy Pond
	A/Northern pintail /Newfoundland/GR679/2011(H3N2)	NOPI NL GR679 2011	10/12/11	Commonwealth Pond
H4	A/American black duck/Newfoundland/807/2008(H4N4)	ABDU NL 807 2008	10/01/08	Mundy Pond
	A/American black duck/Newfoundland/819/2008(H4N6)	ABDU NL 819 2008	09/26/08	Mundy Pond
	A/American black duck/Newfoundland/826/2008(H4N6)	ABDU NL 826 2008	10/08/08	Mundy Pond
	A/American black duck/Newfoundland/MW609/2010(H4N6)	ABDU NL MW609 2010	09/16/10	Mundy Pond
	A/American black duck/Newfoundland/MW861/2010(H4N6)	ABDU NL MW861 2010	12/01/10	Commonwealth Pond
	A/Mallard/Newfoundland/PR021/2010(H4N6)	MALL NL PR021 2010	10/14/10	Commonwealth Pond
H6	A/American black duck/Newfoundland/MW733/2010(H6N6)	ABDU NL MW733 2010	10/14/10	Commonwealth Pond
	A/American black duck/Newfoundland/PR007/2010(H6N6)	ABDU NL PR007 2010	10/15/10	Commonwealth Pond

	A/Mallard and American Black Duck Hybrid /Newfoundland/MW721/2010(H6N8)	M-A Hybrid MW721 NL 2010	09/30/10	Quidi Vidi Lake
H11	A/American black duck/Newfoundland/MW819/2010(H11N3)	ABDU NL MW819 2010	11/22/10	Commonwealth Pond
	A/American black duck/Newfoundland/MW774/2010(H11N9)	ABDU NL MW774 2010	11/08/10	Commonwealth Pond
H12	A/Northern pintail/Newfoundland/GR495/2011(H12)	NOPI NL GR495 2011	09/19/11	Mundy Pond
