# Supplementary material for: Genetic Structure of Avian Influenza Viruses from Ducks of the Atlantic Flyway of North America
Source: PLoS One. 2014 Jan 30;9(1):e86999. doi: 10.1371/journal.pone.0086999 (PMC3907406; doi:10.1371/journal.pone.0086999)
Supplement: Table S2 — Genetic analysis of the 109 Atlantic flyway duck AIVs (from 2006 to 2011). (PDF) [file pone.0086999.s007.pdf]

Table S2. Genetic analysis of the 109 Atlantic flyway duck AIVs (from 2006 to 2011).

HA	Virus identification information	Genotype	Sub-genotype[b]	Gene type by segment[c]							
				PB2	PB1	PA	HA	NP	NA	M	NS
H1	A/American black duck/Newfoundland/1146/2009(H1N1)[a]	CFE1DH1EE2B	CFE1DH1EE2B-1	C-2.1	F-3.1	E-1.1	1D-1.1	H-4.1	1E-1.1	E-1.1	2B-1.1
	A/American black duck/Newfoundland/1148/2009(H1N1)[a]		CFE1DH1EE2B-1	C-2.1	F-3.1	E-1.1	1D-1.1	H-4.1	1E-1.1	E-1.1	2B-1.1
	A/American black duck/Newfoundland/1150/2009(H1N1)[a]		CFE1DH1EE2B-1	C-2.1	F-3.1	E-1.1	1D-1.1	H-4.1	1E-1.1	E-1.1	2B-1.1
	A/domestic duck/Newfoundland/MW668/2010(H1N1)[a]		CFE1DH1EE2B-1	C-2.1	F-3.1	E-1.1	1D-1.1	H-4.1	1E-1.1	E-1.1	2B-1.1
H2	A/Northern pintail/Newfoundland/GR683/2011(H2N2)	CFE2HH2DE2B	CFH2HH2DE2B-1	C-2.2	F-4.1	E-2.1	2H-1.1	H-2.1	2D-1	E-1.2	2B-1.2
	A/mallard/Newfoundland/GR475/2011(H2N2)		CFH2HH2DE2B-2	C-2.2	F-4.1	E-1.2	2H-1.1	H-2.1	2D-1	E-1.2	2B-1.2
	A/mallard/Quebec/10969/2006(H2N3)	CFE2HH3AE1D	CFE2HH3AE1D-1	C-2.11	F-4.6	E-5.1	2H-2.1	H-1.2	3A-2.2	E-1.14	1D-1.7
	A/mallard/Quebec/11063/2006(H2N3)		CFE2HH3AE1D-2	C-3.2	F-1.3	E-5.1	2H-2.1	H-3.3	3A-2.2	E-1.13	1D-1.10
	A/mallard/Quebec/11281/2006(H2N3)		CFE2HH3AE1D-2	C-3.2	F-1.3	E-5.1	2H-2.1	H-3.3	3A-2.2	E-1.13	1D-1.10
	A/American black duck/Newfoundland/836/2008(H2N4)	CFE2HH4AE1D	CFE2HH4AE1D-1	C-2.2	F-4.2	E-2.1	2H-1.2	H-3.1	4A-1.1	E-1.3	1D-1.1
	A/American black duck/Newfoundland/840/2008(H2N4)		CFE2HH4AE1D-1	C-2.2	F-4.2	E-2.1	2H-1.2	H-3.1	4A-1.1	E-1.3	1D-1.1
	A/American black duck/Newfoundland/812/2008(H2N6)	CFH2HH6AE1D	CFH2HH6AE1D-1	C-1.1	F-3.2	H-1.1	2H-1.3	H-3.1	6A-1.1	E-1.3	1D-1.1
H3	A/American black duck/Newfoundland/GR252/2011(H3N2)	CFE3CH2DE2B	CFE3CH2DE2B-1	C-2.2	F-2.1	E-2.1	3C-1.1	H-2.1	2D-1	E-1.4	2B-1.2
	A/American black duck/Newfoundland/GR256/2011(H3N2)		CFE3CH2DE2B-2	C-2.2	F-2.1	E-2.1	3C-1.1	H-5.1	2D-1.2	E-1.4	2B-1.2
	A/American black duck/Newfoundland/GR490/2011(H3N2)		CFE3CH2DE2B-?	C-2.2	F-2.1	E-2.1	3C-1.1	H-2.1	2D	E-1.4	2B-1.2
	A/American black duck/Newfoundland/GR396/2011(H3N2)	CFH3CH2DE2B	CFH3CH2DE2B-1	C-2.2	F-1.1	H-1.2	3C-1.1	H-2.1	2D-1.1	E-1.4	2B-1.2
	A/American black duck/Newfoundland/GR679/2011(H3N2)		CFH3CH2DE2B-2	C-2.5	F-4.1	H-1.5	3C-1.1	H-5.1	2D	E-1.6	2B-1.2
	A/American black duck/Newfoundland and Labrador/26516/2007(H3N2)	CFH3DH2DE1D	CFH3DH2DE1D-1	C-2.10	F-3.2	H-1.6	3D-1.1	H-2.3	2D-1.1	E-l.12	1D-1.1
	A/American black duck/Newfoundland and Labrador/26553/2007(H3N2)		CFH3DH2DE1D-2	C-2.10	F-3.2	H-1.6	3D-1.1	H-3.2	2D-1.1	E-1.12	1D-1.1
	A/American black duck/Quebec/11235/2006(H3N2)	CFH3CH2DE1D	CFH3CH2DE1D-1	C-3.2	F-3.4	H-1.7	3C-2.1	H-6.2	2D-2.1	E-1.3	1D-1.7
	A/mallard/Quebec/11040/2006(H3N2)		CFH3CH2DE1D-2	C-2.12	F-3.4	H-1.7	3C-2.1	H-6.2	2D-2.1	E-1.3	1D-1.7
	A/mallard/Quebec/11045/2006(H3N2)		CFH3CH2DE1D-2	C-2.12	F-3.4	H-1.7	3C-2.1	H-6.2	2D-2.1	E-1.3	1D-1.7

A/mallard/Quebec/11121/2006(H3N2)		CFH3CH2DE1D-2	C-2.12	F-3.4	H-1.7	3C-2.1	H-6.2	2D-2.1	E-1.3	1D-1.7
A/mallard/Quebec/11020/2006(H3N2)		CFH3CH2DE1D-2	C-2.12	F-3.4	H-1.7	3C-2.1	H-6.2	2D-2.1	E-1.3	1D-1.7
A/mallard/Quebec/11247/2006(H3N2)		CFH3CH2DE1D-3	C-3.3	F-4.6	H-1.7	3C-2.1	H-6.2	2D-2.1	E-1.3	1D-1.7
A/mallard/Quebec/11194/2006(H3N2)		CFH3CH2DE1D-3	C-3.3	F-4.6	H-1.7	3C-2.1	H-6.2	2D-2.1	E-1.3	1D-1.7
A/mallard/Quebec/11093/2006(H3N2)		CFH3CH2DE1D-3	C-3.4	F-4.6	H-1.7	3C-2.1	H-6.2	2D-2.1	E-1.3	1D-1.7
A/mallard/Quebec/11221/2006(H3N2)		CFH3CH2DE1D-4	C-2.7	F-2.2	H-1.7	3C-2.1	H-6.2	2D-2.1	E-1.3	1D-1.7
A/American black duck/Newfoundland/MW662/2010(H3N6)	CFE3CH6AE1D	CFE3CH6AE1D-1	C-2.4	F-4.3	E-1.1	3D-1.1	H-1.1	6A-3.1	E-1.1	1D-1.5
A/American black duck/New Brunswick/25182/2007(H3N6)	CFE3CH6AE2B	CFE3CH6AE2B-1	C-2.7	F-6.1	E-1.3	3C-2.3	H-2.4	6A-4.2	E-1.5	2B-1.4
A/mallard/Maryland/1235/2006(H3N6)	CFH3CH6AE1D	CFH3CH6AE1D-1	C-2.7	F-5.1	H-1.10	3C-2.2	H-3.4	6A-4.3	E-1.15	1D-1.12
A/blue-winged teal/New Brunswick/03757/2009(H3N6)		CFH3CH6AE1D-2	C-2.2	F-3.12	H-1.15	3C-2.3	H-1.1	6A-3.2	E-1.20	1D-1.7
A/American black duck/Newfoundland/732/2008(H3N8)	CFE3DH8AE1D	CFE3DH8AE1D-1	C-1.1	F-3.2	E-2.1	3D-1.1	H-3.1	8A	E-1.3	1D-1.1
A/mallard/Nova Scotia/03271/2009(H3N8)		CFE3DH8AE1D-2	C-3.5	F-4.5	E-2.1	3D-1.2	H-4.6	8A-2.1	E-1.19	1D-1.13
A/ring-necked duck/Nova Scotia/03378/2009(H3N8)		CFE3DH8AE1D-2	C-3.5	F-4.5	E-2.1	3D-1.2	H-4.6	8A-2.1	E-1.19	1D-1.13
A/ring-necked duck/New Brunswick/03400/2009(H3N8)		CFE3DH8AE1D-2	C-3.5	F-4.5	E-2.1	3D-1.2	H-4.6	8A-2.1	E-1.19	1D-1.13
A/green-winged teal/New Brunswick/03483/2009(H3N8)		CFE3DH8AE1D-2	C-3.5	F-4.5	E-2.1	3D-1.2	H-4.6	8A-2.1	E-1.19	1D-1.13
A/northern pintail/New Brunswick/03547/2009(H3N8)		CFE3DH8AE1D-2	C-3.5	F-4.5	E-2.1	3D-1.2	H-4.6	8A-2.1	E-1.19	1D-1.13
A/blue-winged teal/Prince Edward Island/03912/2009(H3N8)		CFE3DH8AE1D-2	C-3.5	F-4.5	E-2.1	3D-1.2	H-4.6	8A-2.1	E-1.19	1D-1.13
A/green-winged teal/New Brunswick/02586/2007(H3N8)	CFH3DH8AE1D	CFH3DH8AE1D-1	C-2.19	F-3.6	H-1.17	3D-2.1	H-4.7	8A-2.2	E-1.18	1D-1.9
A/green-winged teal/New Brunswick/02587/2007(H3N8)		CFH3DH8AE1D-1	C-2.19	F-3.6	H-1.17	3D-2.1	H-4.7	8A-2.2	E-1.18	1D-1.1
A/green-winged teal/New Brunswick/02588/2007(H3N8)		CFH3DH8AE1D-1	C-2.19	F-3.6	H-1.17	3D-2.1	H-4.7	8A-2.2	E-1.18	1D-1.1
A/green-winged teal/New Brunswick/02590/2007(H3N8)		CFH3DH8AE1D-1	C-2.19	F-3.6	H-1.17	3D-2.1	H-4.7	8A-2.2	E-1.18	1D-1.1
A/green-winged teal/New Brunswick/02591/2007(H3N8)		CFH3DH8AE1D-1	C-2.19	F-3.6	H-1.17	3D-2.1	H-4.7	8A-2.2	E-1.18	1D-1.1
A/green-winged teal/New Brunswick/02592/2007(H3N8)		CFH3DH8AE1D-1	C-2.19	F-3.6	H-1.17	3D-2.1	H-4.7	8A-2.2	E-1.18	1D-1.1
A/American black duck/Newfoundland/734/2008(H3N8)		CFH3DH8AE1D-2	C-1.1	F-3.2	H-1.1	3D-1.1	H-3.1	8A-2.1	E-1.3	1D-1.1

	A/blue-winged teal/Nova Scotia/03971/2009(H3N8)		CFH3DH8AE1D-3	C-2.2	F-3.11	H-1.17	3D-1.3	H-4.5	8A-2.2	E-1.21	1D-1.13
	A/blue-winged teal/Prince Edward Island/03927/2009(H3N8)		CFH3DH8AE1D-4	C-2.18	F-3.6	H-1.17	3D-2.1	H-1.6	8A-2.2	E-1.18	1D-1.13
	A/mallard/Quebec/11082/2006(H3N8)	CFE3CH8AE2B	CFE3CH8AE2B-1	C-2.7	F-3.7	E-3.1	3C-1.2	H-2.3	8A-1.4	E-1.14	2B-1.3
	A/American black duck/New Brunswick/19350/2006(H3N8)		CFE3CH8AE2B-2	C-2.6	F-3.5	E-4.1	3C-1.2	H-1.8	8A-1.1	E-1.10	2B-1.6
	A/American wigeon/New Brunswick/04487/2007(H3N8)		CFE3CH8AE2B-2	C-2.6	F-3.5	E-4.1	3C-1.2	H-1.8	8A-1.1	E-1.10	2B-1.6
	A/American wigeon/New Brunswick/04488/2007(H3N8)		CFE3CH8AE2B-2	C-2.6	F-3.5	E-4.1	3C-1.2	H-1.8	8A-1.1	E-1.10	2B-1.6
	A/American wigeon/New Brunswick/04489/2007(H3N8)		CFE3CH8AE2B-2	C-2.6	F-3.5	E-4.1	3C-1.2	H-1.8	8A-1.1	E-1.10	2B-1.6
	A/American wigeon/New Brunswick/04490/2007(H3N8)		CFE3CH8AE2B-2	C-2.6	F-3.5	E-4.1	3C-1.2	H-1.8	8A-1.1	E-1.10	2B-1.6
	A/American wigeon/New Brunswick/04491/2007(H3N8)		CFE3CH8AE2B-2	C-2.6	F-3.5	E-4.1	3C-1.2	H-1.8	8A-1.1	E-1.10	2B-1.6
	A/American wigeon/New Brunswick/04492/2007(H3N8)		CFE3CH8AE2B-2	C-2.6	F-3.5	E-4.1	3C-1.2	H-1.8	8A-1.1	E-1.10	2B-1.6
	A/American wigeon/New Brunswick/04493/2007(H3N8)		CFE3CH8AE2B-2	C-2.6	F-3.5	E-4.1	3C-1.2	H-1.8	8A-1.1	E-1.10	2B-1.6
	A/American wigeon/New Brunswick/04494/2007(H3N8)		CFE3CH8AE2B-2	C-2.6	F-3.5	E-4.1	3C-1.2	H-1.8	8A-1.1	E-1.10	2B-1.6
	A/American wigeon/New Brunswick/04497/2007(H3N8)		CFE3CH8AE2B-2	C-2.6	F-3.5	E-4.1	3C-1.2	H-1.8	8A-1.1	E-1.10	2B-1.6
	A/American wigeon/New Brunswick/04500/2007(H3N8)		CFE3CH8AE2B-2	C-2.6	F-3.5	E-4.1	3C-1.2	H-1.8	8A-1.1	E-1.10	2B-1.6
	A/American black duck/Prince Edward Island/14228/2006(H3N8)	CFH3CH8AE2B	CFH3CH8AE2B-1	C-4.1	F-3.6	H-1.1	3C-1.2	H-1.2	8A-1.2	E-1.10	2B-1.6
	A/American black duck/Prince Edward Island/14230/2006(H3N8)		CFH3CH8AE2B-2	C-2.9	F-3.6	H-1.1	3C-1.2	H-1.2	8A-1.2	E-1.10	2B-1.6
	A/mallard/Maryland/807/2007(H3N8)	CFH3CH8AE1D	CFH3CH8AE1D-1	C-2.7	F-5.2	H-1.12	3C-1.3	H-1.7	8A-3.1	E-1.9	1D-1.7
H4	A/blue-winged teal/New Brunswick/03756/2009(H4N2)	CFH4AH2DE2B	CFH4AH2DE2B-1	C-2.8	F-2.1	H-1.16	4A-3.1	H-1.1	2D-1.1	E-1.5	2B-1.7
	A/American black duck/Newfoundland/807/2008(H4N4)	CFH4AH4AE1D	CFH4AH4AE1D-1	C-1.1	F-3.2	H-1.1	4A-1.1	H-1.2	4A-1.1	E-1.3	1D-1.1
	A/American black duck/Newfoundland/819/2008(H4N6)	CFH4AH6AE1D	CFH4AH6AE1D-1	C-1.1	F-3.2	H-1.1	4A-1.1	H-1.2	6A-1.1	E-1.3	1D-1.1
	A/American black duck/Newfoundland/826/2008(H4N6)		CFH4AH6AE1D-1	C-1.1	F-3.2	H-1.1	4A-1.1	H-1.2	6A-1.1	E-1.3	1D-1.1

	A/American black duck/Newfoundland/MW861/2010(H4N6)		CFH4AH6AE1D-2	C-2.3	F-1.1	H-1.5	4A-2.1	H-1.1	6A-2.1	E-1.5	1D-1.5
	A/mallard/Newfoundland/PR021/2010(H4N6)		CFH4AH6AE1D-2	C-2.4	F-1.1	H-1.5	4A-2.1	H-1.1	6A-2.1	E-1.6	1D-1.5
	A/American black duck/Prince Edward Island/14235/2006(H4N6)		CFH4AH6AE1D-3	C-4.1	F-3.2	H-1.1	4A-1.5	H-4.3	6A-1.2	E-1.11	1D-1.6
	A/American black duck/Prince Edward Island/02708/2007(H4N6)		CFH4AH6AE1D-3	C-4.1	F-3.6	H-1.1	4A-1.5	H-4.3	6A-1.3	E-1.11	1D-1.6
	A/mallard/Quebec/11182/2006(H4N6)		CFH4AH6AE1D-4	C-2.12	F-3.2	H-1.9	4A-1.4	H-1.5	6A-3.4	E-1.3	1D-1.9
	A/mallard/Quebec/11189/2006(H4N6)		CFH4AH6AE1D-4	C-2.12	F-3.4	H-1.7	4A-1.4	H-1.5	6A-4.2	E-1.3	1D-1.9
	A/American black duck/Newfoundland/MW609/2010(H4N6)	CFE4AH6AE1D	CFE4AH6AE1D-1	C-2.1	F-3.1	E-1.1	4A-1.2	H-2.1	6A-2.1	E-1.1	1D-1.2
	A/American black duck/New Brunswick/19347/2006(H4N6)		CFE4AH6AE1D-2	C-2.10	F-3.3	E-3.1	4A-1.4	H-2.3	6A-1.1	E-1.8	1D-1.8
	A/American black duck/New Brunswick/19497/2006(H4N6)		CFE4AH6AE1D-3	C-2.7	F-5.1	E-3.2	4A-1.6	H-6.1	6A-4.1	E-1.3	1D-1.7
	A/American black duck/New Brunswick/19502/2006(H4N6)		CFE4AH6AE1D-3	C-2.7	F-5.1	E-3.2	4A-1.6	H-6.2	6A-4.1	E-1.3	1D-1.7
	A/mallard/Quebec/11106/2006(H4N6)		CFE4AH6AE1D-4	C-2.12	F-3.4	E-5.2	4A-1.4	H-1.5	6A-4.2	E-1.3	1D-1.9
	A/mallard/Quebec/11103/2006(H4N6)		CFE4AH6AE1D-4	C-2.12	F-3.4	E-5.2	4A-1.4	H-1.5	6A-4.2	E-1.3	1D-1.9
	A/mallard/Quebec/11102/2006(H4N6)		CFE4AH6AE1D-4	C-2.12	F-3.4	E-5.2	4A-1.4	H-1.5	6A-4.2	E-1.13	1D-1.9
	A/American black duck/Prince Edward Island/02662/2007(H4N6)		CFE4AH6AE1D-5	C-4.1	F-3.6	E-3.1	4A-1.5	H-1.5	6A-1.3	E-1.8	1D-1.6
	A/green-winged teal/New Brunswick/02426/2007(H4N6)		CFE4AH6AE1D-5	C-4.1	F-3.6	E-3.1	4A-1.5	H-1.5	6A-1.3	E-1.8	1D-1.6
	A/mallard/Maryland/965/2006(H4N6)	CFE4AH6AE2B	CFE4AH6AE2B-1	C-2.13	F-6.2	E-2.2	4A-1.3	H-2.4	6A-3.3	E-1.4	2B-1.5
	A/American black duck/New Brunswick/19389/2006(H4N8)	CFE4AH8AE2B	CFE4AH8AE2B-1	C-2.6	F-3.5	E-4.1	4A-1.6	H-6.1	8A-1.1	E-1.3	2B-1.6
	A/American black duck/New Brunswick/19392/2006(H4N8)		CFE4AH8AE2B-2	C-2.6	F-5.1	E-4.1	4A-1.6	H-6.1	8A-1.1	E-1.10	2B-1.6
	A/blue-winged teal/Prince Edward Island/03910/2009(H4N9)	CFE4AH9AE1D	CFE4AH9AE1D-1	C-2.18	F-1.2	E-2.1	4A-3.2	H-1.6	9A-1.2	E-1.3	1D-1.13
H5	A/American black duck/Newfoundland/1181/2009(H5N4)[a]	CFH5CH4AE1D	CFH5CH4AE1D-1	C-2.4	F-3.1	H-1.3	5C-1.1	H-4.2	4A	E-1.7	1D-1.5
	A/mallard/Maryland/802/2007(H5N1)	CFH5CF1EE1D	CFH5CF1EE1D-1	C-2.7	F-7.1	H-1.13	5C-1.2	**F-1.1**	1E- 2.1	E-1.5	1D-1.7
	A/mallard/Ontario/26078/2007(H5N1)	CFH5CH1EE1D	CFH5CH1EE1D-1	C-3.2	F-4.7	H-1.14	5C-1.4	H-1.6	1E- 2.2	E-1.16	1D-1.7
	A/mallard/Maryland/182/2006(H5N2)	CFH5CH2DE1D	CFH5CH2DE1D-1	C-2.14	F-3.7	H-1.11	5C-1.2	H-5.2	2D-1.1	E-1.3	1D-1.10
	A/Muscovy duck/New York/62095-1/2006(H5N2)	CFE5CH2DE1D	CFE5CH2DE1D-1	C-2.12	F-1.4	E-1.5	5C-1.3	H-4.4	2D-1.3	E-1.3	1D-1.1
	A/duck/New York/445743/2006(H5N2)		CFE5CH2DE1D-1	C-2.12	F-1.4	E-1.5	5C-1.3	H-4.4	2D-1.3	E-1.3	1D-1.1
	A/duck/New York/465571/2006(H5N2)	CFE5CH2GE1D	CFE5CH2GE1D-1	C-2.15	F-5.1	E-5.1	5C-1.2	H-4.3	2D-3.1	E-1.5	1D-1.7

	A/northern pintail/Florida/480645-5/2007(H5N2)		CFE5CH2GE1D-2	C-3.3	F-3.8	E-3.4	5C-1.2	H-6.1	2G-2.1	E-1.16	1D-1.7
	A/duck/Pennsylvania/446080-6/2006(H5N2)	CFE5CH2DE1D	CFE5CH2DE1D-1	C-2.16	F-1.4	E-1.5	5C-1.3	H-4.4	2D-1.3	E-1.3	1D-1.1
	A/green winged teal/Delaware/458672-5/2006(H5N2)		CFE5CH2DE1D-2	C-2.17	F-3.9	E-3.5	5C-1.5	H-2.5	2D-1.3	E-1.16	1D-1.10
	A/mallard/Maryland/792/2007(H5N9)	CFH5CH9AE1D	CFH5CH9AE1D	C-2.7	F-5.2	H-1.13	5C-1.2	H-1.7	9A-3.1	E-1.5	1D-1.7
H6	A/American black duck/Newfoundland/MW733/2010(H6N6)	CFH6BH6AE1D	CFH6BH6AE1D-1	C-2.3	F-3.1	H-1.4	**6B-1.1**	H-1.3	6A-3.2	E-1.1	1D-1.4
	A/American black duck/Newfoundland/PR007/2010(H6N6)		CFH6BH6AE1D-2	C-2.1	F-3.1	H-1.5	**6B-1.1**	H-1.1	6A-2.1	E-1.6	1D-1.5
	A/duck/Newfoundland/MW721/2010(H6N8)	CFH6BH8AE1D	CFH6BH8AE1D-1	C-2.1	F-3.1	H-1.4	**6B-1.1**	H-1.3	8A-1.3	E-1.1	1D-1.4
H7	A/American black duck/NB/2538/2007(H7N3)	CFE7FH3AE1D	CFE7FH3AE1D-1	C-2.6	F-4.5	E-1.4	7F-1.1	H-1.5	3A-2.1	E-1.10	1D-1.9
	A/Muscovy duck/New York/19495-7/2006(H7N2)	CFH7FH2GE2B	CFH7FH2GE2B-1	C-5.1	F-3.10	H-1.8	7F-2.1	H-7.1	2G-1.1	E-2.1	2B-2.1
H11	A/American black duck/Newfoundland/MW819/2010(H11N3)	CFH11CH3AE1D	CFH11CH3AE1D-1	C-2.5	F-1.2	H-1.4	11C-1.1	H-2.2	3A-1.1	E-1.5	1D-1.5
	A/American black duck/Newfoundland/MW774/2010(H11N9)	CFH11CH9AE1D	CFH11CH9AE1D-1	C-2.3	F-1.2	H-1.4	11C-1.1	H-2.2	9A-1.1	E-1.5	1D-1.5
	A/mallard/Quebec/11111/2006(H11N9)	CFE11CH9AE1D	CFE11CH9AE1D-1	C-2.6	F-3.7	E-3.3	11C-2.1	H-4.5	9A-2.1	E-1.3	1D-1.11
	A/ring-necked duck/New Brunswick/03449/2009(H11N9)	CFH11CH9AE2B	CFH11CH9AE2B-1	C-3.5	F-3.12	H-1.15	11C-1.2	H-1.9	9A-1.3	E-1.17	2B-1.8
H12	A/Northern pintail/Newfoundland/GR495/2011(H12)	CFE12AH?E1D	CFE12AH?E1D-1	C-3.1	F-4.4	E-2.1	12A-1.1	H-1.4	-	E-1.7	1D-1.3
H13	A/hooded merganser/New Brunswick/03750/2009(H13N6)	JFE13AD6AF1C	JFE13AD6AF1C-1	**J-1.1**	**F-8.1**	**E-6.1**	**13A-1.1**	**D-1.1**	6A-1.2	**F-1.1**	**1C-1.1**
H16	A/mallard/Quebec/02916-1/2009(H16N3)	JFE16DD3DF1C	JFE16DD3DF1C-1	**J-1.2**	**F-8.1**	**E-6.2**	**16D-1.1**	**D-1.1**	**3D-1.1**	**F-1.1**	**1C-1.1**
**Totals**	**109**	**43**	**70**								
	**Gene lineages**			**2**	**1**	**2**	**12**	**3**	**9**	**2**	**3**
	**Gene sub-lineages**			**6**	**8**	**7**	**19**	**9**	**20**	**3**	**4**
	**Gene types**			**29**	**31**	**34**	**38**	**32**	**38**	**23**	**23**

[a] The 4 H1N1 viruses and the H5N4 virus from Newfoundland were sequenced previously (accession numbers KC464555-KC464586, KC492275-KC492290 and KC492307-KC492330).

[b] Repeatedly detected homologous genomes are shaded in grey.

[c] AIV genes of origin other than North American avian are highlighted in colour: Eurasian avian, blue; Eurasian gull, yellow; North American gull, green.
