# Supplementary material for: Genetic Structure of Avian Influenza Viruses from Ducks of the Atlantic Flyway of North America
Source: PLoS One. 2014 Jan 30;9(1):e86999. doi: 10.1371/journal.pone.0086999 (PMC3907406; doi:10.1371/journal.pone.0086999)
Supplement: Table S3 — AIV gene typing summary for Atlantic flyway locations with 20 or more viruses. (PDF) [file pone.0086999.s008.pdf]

Table S3. AIV gene typing summary for Atlantic flyway locations with 20 or more viruses.

Location	Category[a]	HA	NA	PB2	PB1	PA	NP	M	NS	Total
Newfoundland	Gene lineages	9	7	1	1	2	1	1	2	24
	Sub-lineages	10	10	3	4	3	5	1	2	38
	Gene types	13	12	8	9	9	12	8	7	78
Quebec	Gene lineages	4	5	1	1	2	1	1	2	17
	Sub-lineages	6	7	3	5	4	6	2	3	36
	Gene types	6	7	8	7	7	6	4	6	51
New Brunswick	Gene lineages	3	5	2	1	2	2	2	3	19
	Sub-lineages	6	8	4	6	6	5	2	3	40
	Gene types	8	12	9	9	9	11	9	10	81

[a] Gene lineages, sub-lineages and gene types are defined as sharing ≥90%, ≥95% and ≥99% nucleotide identity, respectively.
