# Supplementary material for: Genetic Structure of Avian Influenza Viruses from Ducks of the Atlantic Flyway of North America
Source: PLoS One. 2014 Jan 30;9(1):e86999. doi: 10.1371/journal.pone.0086999 (PMC3907406; doi:10.1371/journal.pone.0086999)
Supplement: Table S4 — AIV genotyping for Atlantic flyway locations with 20 or more viruses. (PDF) [file pone.0086999.s009.pdf]

Table S4. AIV genotyping for Atlantic flyway locations with 20 or more viruses.

Location	Viruses	Genotypes	Sub-genotypes	Repeatedly detected sub-genotypes
Newfoundland	31	19	24	3
Quebec	20	6	10	4
New Brunswick	32	11	14	3
