# Supplementary material for: Genetic Structure of Avian Influenza Viruses from Ducks of the Atlantic Flyway of North America
Source: PLoS One. 2014 Jan 30;9(1):e86999. doi: 10.1371/journal.pone.0086999 (PMC3907406; doi:10.1371/journal.pone.0086999)
Supplement: Table S5 — Repeated gene type detections across space and time in ducks of the Atlantic flyway over 2006–2011. (PDF) [file pone.0086999.s010.pdf]

Table S5. Repeated gene type detections across space and time in ducks of the Atlantic flyway over 2006-2011.

Segment	Gene type	Detection year/location[a]	Duration (years)
**PB2**	C-2.1	2009/NL, 2010/NL	2
	C-2.2	2008/NL, 2009/NB, 2011/NL	3
	C-2.4	2009/NL, 2010/NL	2
	C-2.5	2010/NL, 2011/NL	2
	C-2.6	2006/QC, 2006/NB, 2006/NB, 2007/NB	2
	C-2.7	2006/QC, 2006/MD, 2006/NB, 2007/MD, 2007/NB	2
	C-2.8	2007/MD, 2007/NB, 2009/NB	3
	C-2.10	2006/NB, 2007/NL	2
	C-3.2	2006/QC, 2007/ON	2
	C-3.3	2006/QC, 2007/FL	2
	C-3.5	2009/PEI, 2009/NS, 2009/QC	1
	C-4.1	2007/NB, 2007/PEI	1
**PB1**	F-1.1	2010/NL, 2010/NL, 2011/NL	2
	F-3.1	2009/NL, 2010/NL	2
	F-3.2	2006/PEI, 2007/NL, 2008/NL	3
	F-3.5	2006/NB, 2007/NB	2
	F-3.6	2007/PEI, 2009/PEI	3
	F-3.8	2006/QC, 2007/FL	2
	F-3.12	2009/PEI, 2009/NB	1
	F-4.5	2007/NB, 2009/NS, 2009/NB	3
	F-5.1	2006/NB, 2006/NY, 2006/MD, 2007/MD	2
	F-8.1	2009/QC, 2009/NB	1
**PA**	E-1.1	2009/NL, 2010/NL	2
	E-1.5	2006/NY, 2006/PA	1
	E-2.1	2008/NL, 2009/PEI, 2009/NS, 2009/NB, 2011/NL	4
	E-3.1	2006/QC, 2006/PEI	1
	E-5.1	2006/QC, 2006/NY, 2006/NB	1
	H-1.1	2006/PEI, 2008/NL	3
	H-1.5	2010/NL, 2011/NL	2
	H-1.15	2009/PEI, 2009/NB	1
	H-1.17	2007/NB, 2009/NB, 2009/PEI	3
	E-4.1	2006/NB, 2007/NB	2
**NP**	H-1.1	2009/PEI, 2009/NB, 2010/NL	2
	H-1.2	2006/QC, 2006/PEI, 2008/NL	3
	H-1.5	2006/QC, 2006/NB, 2007/NB	2
	H-1.8	2006/NB, 2007/NB	2
	H-2.1	2010/NL, 2011/NL	2
	H-2.3	2006/QC, 2007/NL	2
	H-2.4	2006/MD, 2007/NB	2
	H-4.3	2006/PEI, 2006/NY	1
	H-4.4	2006/NY, 2006/PA	1

	H-4.6	2009/PEI, 2009/NS, 2009/NB	1
	H-6.1	2006/NB, 2007/FL	2
	H-6.2	2006/QC, 2006/NB	1
**M**	E-1.1	2009/NL, 2010/NL	2
	E-1.3	2006/QC, 2006/MD, 2006/NB, 2006/NY, 2006/PA, 2008/NL, 2009/PEI	4
	E-1.5	2006/NY, 2007/MD, 2007/NB, 2009/NB, 2010/NL	5
	E-1.6	2010/NL, 2011/NL	2
	E-1.7	2009/NL, 2011/NL	3
	E-1.8	2006/NB, 2007/NB, 2007/PEI	2
	E-1.10	2006/NB, 2007/NB	2
	E-1.16	2006/DE, 2007/ON, 2007/FL	2
	E-1.18	2007/NB, 2009/PEI	3
	E-1.19	2009/PEI, 2009/NS, 2009/NB	1
	J-1.1	2009/QC, 2009/NB	1
**NS**	1D-1.1	2006/NY, 2007/NL, 2008/NL	3
	1D-1.5	2009/NL, 2010/NL	2
	1D-1.6	2006/PEI, 2007/NB, 2007/PEI	2
	1D-1.7	2006/QC, 2006/NB, 2007/FL, 2007/ON, 2009/PEI	4
	1D-1.9	2006/QC, 2006/PA, 2007/NB	2
	1D-1.10	2006/QC, 2006/MD, 2006/DE	1
	1D-1.13	2009/PEI, 2009/NS, 2009/NB	1
	1C-1.1	2009/QC, 2009/NB	1
	2B-1.6	2006/NB, 2006/PEI, 2007/NB	2
**HA**	3C-1.2	2006/QC, 2006/NB, 2006/PEI, 2007/NB	2
	3C-2.3	2007/NB, 2009/PEI	3
	3D-1.1	2007/NL, 2008/NL, 2010/NL	4
	3D-1.2	2009/PEI, 2009/NS, 2009/NS	1
	3D-2.1	2007/NB, 2009/PEI	3
	4A-1.4	2006/QC, 2006/NB	1
	4A-1.5	2006/PEI, 2007/NB, 2007/PEI	2
	5C-1.2	2006/MD, 2007/MD, 2007/FL	2
	5C-1.3	2006/NY, 2006/PA	1
**NA**	2D-1.1	2006/MD, 2007/NL, 2009/NB, 2011/NL	6
	2D-1.3	2006/NY, 2006/PA, 2006/DE	1
	6A-1.1	2006/NB, 2008/NL	3
	6A-1.2	2006/PEI, 2009/NB	4
	6A-3.2	2009/PEI, 2010/NL	2
	6A-4.2	2006/QC, 2007/NB	2
	8A-2.1	2008/NL, 2009/NS, 2009/NB	2
	8A-2.2	2007/NB, 2009/PEI, 2009/NB	3

[a] NL, Newfoundland; QC, Quebec; MD, Maryland; NB, New Brunswick; PEI, Prince Edward Island; NY, New York; PA, Pennsylvania; DE, Delaware; ON, Ontario; FL, Florida; NS, Nova Scotia
