# Supplementary material for: Genetic Structure of Avian Influenza Viruses from Ducks of the Atlantic Flyway of North America
Source: PLoS One. 2014 Jan 30;9(1):e86999. doi: 10.1371/journal.pone.0086999 (PMC3907406; doi:10.1371/journal.pone.0086999)
Supplement: Table S6 — Detection of the 248 Atlantic flyway AIV gene types through years in North American flyways. (PDF) [file pone.0086999.s011.pdf]

Table S6. Detection of the 248 Atlantic flyway AIV gene types through years in North American flyways.

Segment	Gene type	Detection in NCBI database	Detection of the Newfoundland AIV gene types over 2007-2011	Total period	Duration (years)
PB2	C-1.1	No	2008	2008	1
	C-2.1	2005-2010	2009-2010	2005-2010	6
	C-2.2	2004-2009	2008-2011	2004-2011	8
	C-2.3	2003-2009	2010	2003-2010	8
	C-2.4	No	2009-2010	2009-2010	2
	C-2.5	2000-2010	2010-2011	2000-2011	12
	C-2.6	2003-2007	No	2003-2007	5
	C-2.7	2003-2009	No	2003-2009	7
	C-2.8	2007-2009	No	2007-2009	3
	C-2.9	2003-2006	No	2003-2006	4
	C-2.10	2002-2007	2007	2002-2007	6
	C-2.11	2005-2007	No	2005-2007	3
	C-2.12	2005-2007	No	2005-2007	3
	C-2.13	1985-2007	No	1985-2007	23
	C-2.14	2005-2006	No	2005-2006	2
	C-2.15	2006-2009	No	2006-2009	4
	C-2.16	2005-2007	No	2005-2007	3
	C-2.17	2000-2006	No	2000-2006	7
	C-2.18	2005-2009	No	2005-2009	5
	C-2.19	2004-2007	No	2004-2007	4
	C-3.1	1983-2010	2011	1983-2010	29
	C-3.2	2005-2007	No	2005-2007	3
	C-3.3	2006	No	2006	1
	C-3.4	2005-2008	No	2005-2008	4
	C-3.5	2006-2009	No	2006-2009	4
	C-4.1	2005-2007	No	2005-2007	3
	C-5.1	2003-2006	No	2003-2006	4
	J-1.1	2009	No	2009	1
	J-1.2	2005-2009	No	2005-2009	5
PB1	F-1.1	2006-2010	2010-2011	2006-2011	6
	F-1.2	2006-2009	2010	2006-2010	5
	F-1.3	2006-2009	No	2006-2009	4
	F-1.4	2004-2007	No	2004-2007	4
	F-2.1	2003-2010	2011	2003-2011	9
	F-2.2	2006	No	2006	1
	F-3.1	2006-2009	2009-2010	2006-2010	5
	F-3.2	2003-2008	2007-2008	2003-2008	6
	F-3.3	2002-2009	No	2002-2009	8
	F-3.4	2002-2008	No	2002-2008	7
	F-3.5	2003-2007	No	2003-2007	5
	F-3.6	2002-2009	No	2002-2009	8
	F-3.7	2002-2009	No	2002-2009	8

	F-3.8	2006-2008	No	2006-2008	3
	F-3.9	2002-2008	No	2002-2008	7
	F-3.10	2005-2006	No	2005-2006	2
	F-3.11	2002-2009	No	2002-2009	8
	F-3.12	2006-2009	No	2006-2009	4
	F-4.1	1999-2008	2011	1999-2011	13
	F-4.2	2007-2009	2008	2007-2009	3
	F-4.3	2009	2010	2009-2010	2
	F-4.4	2009-2010	2011	2009-2011	3
	F-4.5	2006-2009	No	2006-2009	4
	F-4.6	2006-2010	No	2006-2010	5
	F-4.7	1997-1010	No	1997-1010	14
	F-5.1	2005-2009	No	2005-2009	5
	F-5.2	2006-2009	No	2006-2009	4
	F-6.1	2005-2008	No	2005-2008	4
	F-6.2	2003-2007	No	2003-2007	5
	F-7.1	2005-2007	No	2005-2007	3
	F-8.1	2008-2009	No	2008-2009	2
PA	H-1.1	2002-2009	2010	2002-2010	9
	H-1.2	2006-2010	2008	2006-2010	5
	H-1.3	2005-2009	2009	2005-2009	5
	H-1.4	2002-2009	2010	2002-2010	9
	H-1.5	2009-2010	2010-2011	2009-2011	3
	H-1.6	2005-2009	2007	2005-2009	5
	H-1.7	2000-2006	No	2000-2006	7
	H-1.8	2004-2006	No	2004-2006	3
	H-1.9	2005-2010	No	2005-2010	6
	H-1.10	2006-2009	No	2006-2009	4
	H-1.11	1999-2009	No	1999-2009	11
	H-1.12	2003-2009	No	2003-2009	7
	H-1.13	2005-2007	No	2005-2007	3
	H-1.14	2006-2008	No	2006-2008	3
	H-1.15	1997-2010	No	1997-2010	14
	H-1.16	2006-2009	No	2006-2009	4
	H-1.17	2005-2009	No	2005-2009	5
	E-1.1	2006-2009	2009-2010	2006-2010	5
	E-1.2	2006-2010	2011	2006-2011	5
	E-1.3	2006-2009	No	2006-2009	4
	E-1.4	2006-2008	No	2006-2008	3
	E-1.5	2005-2010	No	2005-2010	6
	E-2.1	2007-2009	2008-2011	2007-2011	5
	E-2.2	1998-2008	No	1998-2008	11
	E-3.1	2004-2010	No	2004-2010	7
	E-3.2	2002-2007	No	2002-2007	6
	E-3.3	2004-2009	No	2004-2009	6
	E-3.4	2006-2009	No	2006-2009	4

	E-3.5	2000-2006	No	2000-2006	7
	E-4.1	2005-2007	No	2005-2007	3
	E-5.1	2002-2008	No	2002-2008	7
	E-5.2	2002-2007	No	2002-2007	6
	E-6.1	2006-2009	No	2006-2009	4
	E-6.2	1998-2009	No	1998-2009	12
HA	1D-1.1	2007-2009	2009	2007-2009	3
	2H-1.1	2008-2009	2011	2008-2011	4
	2H-1.2	2007	2008	2007-2008	2
	2H-1.3	2008	2008	2008	1
	2H-2.1	2006	2006	2006	1
	3C-1.1	2004-2011	2011	2004-2011	8
	3C-1.2	2005-2007	No	2005-2007	3
	3C-1.3	2006-2009	No	2006-2009	4
	3C-2.1	2005-2006	No	2005-2006	2
	3C-2.2	2002-2008	No	2002-2008	7
	3C-2.3	2007-2009	No	2007-2009	3
	3D-1.1	2007-2009	2007-2010	2007-2010	4
	3D-1.2	2008-2009	No	2008-2009	2
	3D-1.3	2005-2009	No	2005-2009	5
	3D-2.1	2007-2009	No	2007-2009	3
	4A-1.1	NO	2008	2008	1
	4A-1.2	NO	2010	2010	1
	4A-1.3	2002-2006	No	2002-2006	5
	4A-1.4	2002-2008	No	2002-2008	7
	4A-1.5	2002-2007	No	2002-2007	6
	4A-1.6	2006	No	2006	1
	4A-2.1	2008-2009	2010	2008-2010	3
	4A-3.1	2005-2009	No	2005-2009	5
	4A-3.2	2007-2009	No	2007-2009	3
	5C-1.1	NO	2009	2009	1
	5C-1.2	2004-2009	No	2004-2009	6
	5C-1.3	2004-2007	No	2004-2007	4
	5C-1.4	2004-2009	No	2004-2009	6
	5C-1.5	2004-2009	No	2004-2009	6
	6B-1.1	2008	No	2008	1
	7F-1.1	2005-2008	No	2005-2008	4
	7F-2.1	2004-2006	No	2004-2006	3
	11C-1.1	NO	2010	2010	1
	11C-1.2	2007-2009	No	2007-2009	3
	11C-2.1	2002-2006	No	2002-2006	5
	12A-1.1	NO	2011	2011	1
	13A-1.1	2009	No	2009	1
	16D-1.1	2009	No	2009	1
NA	1E-1.1	2007-2009	2009	2007-2009	3
	1E-2.1	2006-2009	No	2006-2009	4

	1E-2.2	2006-2009	No	2006-2009	4
	2D-1.1	2006-2009	2007-2011	2006-2011	6
	2D-1.2	No	2011	2011	1
	2D-1.3	2000-2009	No	2000-2009	10
	2D-2.1	2003-2006	No	2003-2006	4
	2D-3.1	2001-2009	No	2001-2009	9
	2G-1.1	2005-2006	No	2005-2006	2
	2G-1.2	2001-2010	No	2001-2010	10
	3A-1.1	1978	2010	1978-2010	33
	3A-2.1	2003-2007	No	2003-2007	5
	3A-2.2	2004-2008	No	2004-2008	6
	3D-1.1	2009	No	2009	1
	4A-1.1	2008	2008	2008	1
	6A-1.1	1999-2007	2008	1999-2008	10
	6A-1.2	2009	No	2009	1
	6A-1.3	2005-2007	No	2005-2007	3
	6A-2.1	2007-2009	2010	2007-2010	4
	6A-3.1	2006-2009	2010	2006-2010	5
	6A-3.2	2004-2009	2010	2004-2010	7
	6A-3.3	2006	No	2006	1
	6A-3.4	2004-2009	No	2004-2009	6
	6A-4.1	1998-2008	No	1998-2008	11
	6A-4.2	2002-2009	No	2002-2009	8
	6A-4.3	2002-2007	No	2002-2007	6
	8A-1.1	2005-2007	No	2005-2007	3
	8A-1.2	2005-2007	No	2005-2007	3
	8A-1.3	2005-2006	2010	2005-2010	6
	8A-1.4	2005-2007	No	2005-2007	3
	8A-2.1	2006-2009	2008	2006-2009	4
	8A-2.2	2007-2009	No	2007-2009	3
	8A-3.1	2005-2009	No	2005-2009	5
	9A-1.1	2007-2009	No	2007-2009	3
	9A-1.2	2007-2010	No	2007-2010	4
	9A-1.3	2007-2010	No	2007-2010	4
	9A-2.1	2005-2006	No	2005-2006	2
	9A-3.1	2006-2009	No	2006-2009	4
NP	F-1.1	2001-2011	No	2001-2011	11
	H-1.1	2006-2009	2010	2006-2010	5
	H-1.2	2001-2009	2008	2001-2009	9
	H-1.3	No	2010	2010	1
	H-1.4	2005-2009	2011	2005-2011	7
	H-1.5	1985-2007	No	1985-2007	23
	H-1.6	2003-2009	No	2003-2009	7
	H-1.7	1999-2009	No	1999-2009	11
	H-1.8	2006-2007	No	2006-2007	2
	H-1.9	2006-2009	No	2006-2009	4

	H-2.1	2008-2009	2010-2011		2008-2011	4
	H-2.2	2006-2007	2010		2006-2010	5
	H-2.3	2005-2008	2007		2005-2008	4
	H-2.4	2006-2010	No		2006-2010	5
	H-2.5	2005-2009	No		2005-2009	5
	H-3.1	2006-2009	2008		2006-2009	4
	H-3.2	2006-2007	2007		2006-2007	2
	H-3.3	2006-2007	No		2006-2007	2
	H-3.4	2006-2007	No		2006-2007	2
	H-4.1	2003-2009	2009		2003-2009	7
	H-4.2	2004-2009	2009		2004-2009	6
	H-4.3	2005-2007	No		2005-2007	3
	H-4.4	2003-2009	No		2003-2009	7
	H-4.5	2004-2009	No		2004-2009	6
	H-4.6	2003-2009	No		2003-2009	7
	H-4.7	2004-2009	No		2004-2009	6
	H-5.1	2009	2011		2009-2011	3
	H-5.2	2004-2006	No		2004-2006	3
	H-6.1	2001-2009	No		2001-2009	9
	H-6.2	2002-2009	No		2002-2009	8
	H-7.1	2005-2006	No		2005-2006	2
	D-1.1	1999-2009	No		1999-2009	11
M	E-1.1	2006-2010	2009-2010		2006-2010	5
	E-1.2	2006-2009	2011		2006-2011	6
	E-1.3	1985-2009	2008		1985-2009	25
	E-1.4	2006	No		2006	1
	E-1.5	1998-2009	2010		1998-2010	13
	E-1.6	2004-2010	2010-2011		2004-2011	8
	E-1.7	2005-2010	2009-2011		2005-2011	7
	E-1.8	1998-2009	No		1998-2009	12
	E-1.9	1996-2009	No		1996-2009	14
	E-1.10	1998-2009	No		1998-2009	12
	E-1.11	1996-2007	No		1996-2007	12
	E-l.12	1998-2010	2007		1998-2010	13
	E-1.13	2002-2010	No		2002-2010	9
	E-1.14	1998-2009	No		1998-2009	12
	E-1.15	1998-2007	No		1998-2007	10
	E-1.16	2001-2007	No		2001-2007	7
	E-1.17	1998-2009	No		1998-2009	12
	E-1.18	1996-2009	No		1996-2009	14
	E-1.19	1987-2009	No		1987-2009	13
	E-1.20	1991-2010	No		1991-2010	20
	E-1.21	1986-2009	No		1986-2009	24
	E-2.1	2005-2006	No		2005-2006	2
	J-1.1	2005-2009	No		2005-2009	4
NS	1D-1.1	2003-2010	2007-2008		2003-2010	8

	1D-1.2	2005-2010	2010	2005-2010	6
	1D-1.3	2005-2010	2011	2005-2011	7
	1D-1.4	1987-2010	2010	1987-2010	24
	1D-1.5	1999-2010	2009-2010	1999-2010	12
	1D-1.6	1998-2007	No	1998-2007	10
	1D-1.7	1988-2009	No	1988-2009	22
	1D-1.8	1995-2010	No	1995-2010	16
	1D-1.9	2002-2009	No	2002-2009	8
	1D-1.10	1999-2007	No	1999-2007	9
	1D-1.11	2002-2009	No	2002-2009	8
	1D-1.12	1998-2006	No	1998-2006	9
	1D-1.13	1999-2010	No	1999-2010	12
	1C-1.1	1999-2009	No	1999-2009	11
	2B-1.1	1998-2009	2009	1998-2009	12
	2B-1.2	1985-2009	2009-2011	1985-2011	27
	2B-1.3	1985-2009	2011	1985-2011	27
	2B-1.4	1998-2008	No	1998-2008	11
	2B-1.5	1998-2008	No	1998-2008	11
	2B-1.6	2000-2007	No	2000-2007	8
	2B-1.7	2002-2009	No	2002-2009	8
	2B-1.8	1991-2009	No	1991-2009	19
	2B-2.1	2004-2006	No	2004-2006	3
Total	248	1978-2011	2007-2011	1978-2011	34
