# Supplementary material for: Genetic Structure of Avian Influenza Viruses from Ducks of the Atlantic Flyway of North America
Source: PLoS One. 2014 Jan 30;9(1):e86999. doi: 10.1371/journal.pone.0086999 (PMC3907406; doi:10.1371/journal.pone.0086999)
Supplement: Table S7 — Detection of the 248 identified Atlantic flyway AIV gene types in the North American flyways. (PDF) [file pone.0086999.s012.pdf]

Table S7. Detection of the 248 identified Atlantic flyway AIV gene types in the North American flyways.

**Gene type**	**Flyway**			
**PB2**	**Atlantic**	**Mississippi**	**Central**	**Pacific**
C-1.1	6	0	0	0
C-2.1	8	16	14	2
C-2.2	33	18	0	0
C-2.3	26	49	4	9
C-2.4	3	0	0	0
C-2.5	10	20	15	16
C-2.6	41	12	4	0
C-2.7	53	20	3	0
C-2.8	1	3	0	0
C-2.9	2	2	3	0
C-2.10	10	0	1	0
C-2.11	7	2	0	0
C-2.12	32	2	2	0
C-2.13	19	4	1	0
C-2.14	2	0	0	0
C-2.15	22	26	17	4
C-2.16	27	2	1	0
C-2.17	1	1	0	0
C-2.18	6	1	0	0
C-2.19	14	1	3	1
C-3.1	18	62	17	63
C-3.2	11	1	0	0
C-3.3	2	0	0	0
C-3.4	1	2	0	0
C-3.5	9	11	1	4
C-4.1	12	0	0	0
C-5.1	99	0	0	0
J-1.1	4	0	0	0
J-1.2	13	0	0	0
**Total**	**492**	**255**	**86**	**99**
**PB1**	**Atlantic**	**Mississippi**	**Central**	**Pacific**
F-1.1	3	1	7	2
F-1.2	4	1	2	9
F-1.3	4	6	8	5
F-1.4	36	7	0	0
F-2.1	4	47	11	21
F-2.2	1	0	0	0
F-3.1	8	39	0	0
F-3.2	39	1	0	0
F-3.3	54	45	0	0
F-3.4	16	28	0	0
F-3.5	22	6	0	0

F-3.6	54	45	0	0
F-3.7	32	41	0	0
F-3.8	1	2	1	4
F-3.9	18	35	0	0
F-3.10	128	0	0	0
F-3.11	1	1	0	0
F-3.12	1	2	3	0
F-4.1	5	25	0	0
F-4.2	2	28	1	0
F-4.3	1	2	0	0
F-4.4	1	2	4	3
F-4.5	9	3	1	0
F-4.6	9	19	15	5
F-4.7	1	5	4	24
F-5.1	35	54	6	6
F-5.2	35	53	2	4
F-6.1	24	0	0	0
F-6.2	35	4	1	0
F-7.1	4	4	0	0
F-8.1	6	0	0	0
**Total**	**593**	**506**	**66**	**83**

**PA**	**Atlantic**	**Mississippi**	**Central**	**Pacific**
H-1.1	86	23	11	24
H-1.2	1	1	7	15
H-1.3	11	1	1	1
H-1.4	77	17	9	2
H-1.5	10	1	2	0
H-1.6	4	2	0	0
H-1.7	10	0	2	0
H-1.8	131	0	0	0
H-1.9	6	12	4	30
H-1.10	2	1	0	0
H-1.11	13	5	16	12
H-1.12	16	36	11	27
H-1.13	3	1	1	1
H-1.14	27	39	4	6
H-1.15	4	7	13	16
H-1.16	6	9	4	33
H-1.17	72	7	3	4
E-1.1	6	78	16	2
E-1.2	3	27	10	8
E-1.3	1	78	16	2
E-1.4	2	1	32	0
E-1.5	14	19	6	49
E-2.1	35	24	2	0
E-2.2	18	19	2	1

E-3.1	22	49	15	0
E-3.2	52	12	0	0
E-3.3	21	48	11	0
E-3.4	1	4	4	0
E-3.5	7	6	1	1
E-4.1	12	0	0	0
E-5.1	76	6	2	0
E-5.2	47	9	0	0
E-6.1	7	0	0	0
E-6.2	4	0	0	0
**Total**	**807**	**542**	**205**	**234**

**HA**	**Atlantic**	**Mississippi**	**Central**	**Pacific**
1D-1.1	3	11	5	3
2H-1.1	2	6	0	0
2H-1.2	2	1	0	0
2H-1.3	1	0	0	1
2H-2.1	3	0	0	0
3C-1.1	6	15	3	0
3C-1.2	8	0	0	0
3C-1.3	1	7	2	0
3C-2.1	9	0	0	0
3C-2.2	5	14	0	0
3C-2.3	2	0	0	0
3D-1.1	23	0	0	0
3D-1.2	16	0	0	0
3D-1.3	7	0	0	0
3D-2.1	8	0	0	0
4A-1.1	3	0	0	0
4A-1.2	1	0	0	0
4A-1.3	9	3	1	0
4A-1.4	36	5	0	0
4A-1.5	4	1	0	0
4A-1.6	4	0	0	0
4A-2.1	2	2	1	2
4A-3.1	1	0	1	0
4A-3.2	7	93	43	1
5C-1.1	4	3	0	2
5C-1.2	63	37	5	0
5C-1.3	15	8	1	0
5C-1.4	44	15	1	0
5C-1.5	47	38	5	0
6B-1.1	3	0	0	0
7F-1.1	1	0	0	0
7F-2.1	76	0	0	0
11C-1.1	2	0	0	0
11C-1.2	1	10	0	0

11C-2.1	16	4	0	0
12A-1.1	1	0	0	0
13A-1.1	4	0	0	0
16D-1.1	1	0	0	0
**Total**	**441**	**273**	**68**	**9**
**NP**	**Atlantic**	**Mississippi**	**Central**	**Pacific**
F-1.1	3	1	1	75
H-1.1	8	14	17	4
H-1.2	42	34	30	11
H-1.3	2	0	0	0
H-1.4	14	23	9	13
H-1.5	32	1	0	1
H-1.6	4	8	3	24
H-1.7	21	16	18	2
H-1.8	4	0	0	0
H-1.9	2	4	0	0
H-2.1	6	1	0	3
H-2.2	4	10	6	2
H-2.3	15	4	4	7
H-2.4	4	12	7	7
H-2.5	9	12	4	31
H-3.1	7	2	0	1
H-3.2	2	1	0	1
H-3.3	2	2	0	1
H-3.4	5	1	0	1
H-4.1	7	7	0	0
H-4.2	8	1	0	0
H-4.3	30	3	1	0
H-4.4	21	10	0	0
H-4.5	21	12	0	0
H-4.6	17	3	0	0
H-4.7	29	11	0	0
H-5.1	2	1	0	0
H-5.2	18	3	0	0
H-6.1	34	29	5	5
H-6.2	42	43	6	19
H-7.1	94	0	0	0
D-1.1	7	0	0	0
**Total**	**516**	**269**	**111**	**208**
**NA**	**Atlantic**	**Mississippi**	**Central**	**Pacific**
1E-1.1	5	31	1	7
1E- 2.1	7	16	6	30
1E- 2.2	7	15	6	21
2D-1.1	5	19	1	18
2D-1.2	1	0	0	0
2D-1.3	17	16	0	0

2D-2.1	10	0	0	0
2D-3.1	39	34	4	0
2G-1.1	98	0	0	0
2G-1.2	10	31	1	2
3A-1.1	1	0	1	0
3A-2.1	6	0	0	0
3A-2.2	10	4	2	15
3D-1.1	1	0	0	0
4A-1.1	5	0	0	0
6A-1.1	14	1	0	0
6A-1.2	4	0	0	0
6A-1.3	10	0	0	0
6A-2.1	23	0	0	0
6A-3.1	3	1	4	0
6A-3.2	22	8	27	19
6A-3.3	4	2	0	0
6A-3.4	8	6	19	15
6A-4.1	16	7	5	0
6A-4.2	16	7	1	0
6A-4.3	5	6	0	0
8A-1.1	8	0	0	0
8A-1.2	8	0	0	0
8A-1.3	4	0	0	0
8A-1.4	7	0	0	0
8A-2.1	10	8	0	0
8A-2.2	10	0	0	0
8A-3.1	3	18	12	2
9A-1.1	4	12	0	0
9A-1.2	4	20	0	0
9A-1.3	4	22	0	0
9A-2.1	6	0	0	0
9A-3.1	1	1	0	0
**Total**	**416**	**285**	**90**	**129**
**M**	**Atlantic**	**Mississippi**	**Central**	**Pacific**
E-1.1	9	16	12	14
E-1.2	6	7	1	3
E-1.3	115	23	2	25
E-1.4	3	0	0	0
E-1.5	135	80	16	22
E-1.6	7	20	9	40
E-1.7	11	57	16	109
E-1.8	33	46	20	5
E-1.9	138	78	15	19
E-1.10	115	87	22	26
E-1.11	11	9	2	3
E-l.12	121	76	27	28

E-1.13	12	52	34	143
E-1.14	59	52	16	24
E-1.15	6	1	2	4
E-1.16	6	6	6	0
E-1.17	102	81	24	18
E-1.18	69	15	10	6
E-1.19	21	1	9	18
E-1.20	2	17	13	16
E-1.21	11	2	7	0
E-2.1	107	0	0	0
J-1.1	10	0	0	0
**Total**	**1109**	**726**	**263**	**523**

**NS**	**Atlantic**	**Mississippi**	**Central**	**Pacific**
1D-1.1	52	10	6	7
1D-1.2	3	31	6	17
1D-1.3	1	12	3	3
1D-1.4	60	35	9	16
1D-1.5	20	15	6	23
1D-1.6	30	26	3	0
1D-1.7	90	52	10	8
1D-1.8	91	54	13	8
1D-1.9	44	11	7	8
1D-1.10	10	7	8	26
1D-1.11	13	11	0	2
1D-1.12	2	6	1	0
1D-1.13	12	18	6	37
1C-1.1	6	0	0	0
2B-1.1	8	15	28	0
2B-1.2	22	27	36	10
2B-1.3	15	16	4	38
2B-1.4	6	17	0	0
2B-1.5	9	14	2	38
2B-1.6	10	7	2	37
2B-1.7	14	4	0	0
2B-1.8	7	4	14	25
2B-2.1	97	0	0	0
**Total**	**622**	**392**	**164**	**303**
