# Supplementary material for: Genetic Structure of Avian Influenza Viruses from Ducks of the Atlantic Flyway of North America
Source: PLoS One. 2014 Jan 30;9(1):e86999. doi: 10.1371/journal.pone.0086999 (PMC3907406; doi:10.1371/journal.pone.0086999)
Supplement: Table S8 — Summary of detections of the Atlantic Flyway duck AIV genes in non-duck hosts. (PDF) [file pone.0086999.s013.pdf]

Table S8. Summary of detections of the Atlantic Flyway duck AIV genes in non-duck hosts.

Sub-lineage	Number of gene types	Gene types found outside ducks
PB2		
C-1	1	ND[a]
C-2	19	C-2.2, 2.3, 2.5, 2.7, 2.12, 2.13, 2.15, 2.16, 2.19
C-3	5	C-3.1, 3.5
C-4	1	C-4.1
C-5	1	C-5.1
J-1	2	J-1.1
Total	29	14
PB1		
F-1	4	F-1.3, 1.4
F-2	2	F-2.1
F-3	12	F-3.2, 3.3, 3.4, 3.5, 3.6, 3.7, 3.9, 3.10
F-4	7	F-4.5, 4.7
F-5	2	F-5.1, 5.2
F-6	2	F-6.1, 6.2
F-7	1	ND
F-8	1	F-8.1
Total	31	18
PA		
H-1	17	H-1.1, 1.5, 1.8, 1.9, 1.11, 1.12, 1.13, 1.14, 1.16, 1.17
E-1	5	E-1.1, 1.4, 1.5
E-2	2	E-2.1, 2.2
E-3	5	E-3.1, 3.2, 3.3
E-4	1	ND
E-5	2	E-5.1, 5.2
E-6	2	E-6.1, 6.2
Total	34	22
HA		
1D-1	1	1D-1.1
2H-1	3	2H-1.2
2H-2	1	ND
3C-1	3	3C-1.2
3C-2	3	ND
3D-1	3	3D-1.1
3D-2	1	ND
4A-1	6	4A-1.1, 1.4
4A-2	1	ND
4A-3	2	4A-3.2
5C-1	5	5C-1.2, 1.3, 1.4, 1.5
6B-1	1	6B-1.1
7F-1	1	ND
7F-2	1	7F-2.1

11C-1	2	ND
11C-2	1	ND
12A-1	1	ND
13A-1	1	13A-1.1
16D-1	1	ND
Total	38	14
NP		
F-1	1	F-1.1
H-1	9	H-1.2, 1.4, 1.5, 1.6, 1.7
H-2	5	H-2.3, 2.4, 2.5
H-3	4	H-3.1, 3.4
H-4	7	H-4.3, 4.4, 4.5, 4.6
H-5	2	H-5.2
H-6	2	H-6.2
H-7	1	H-7.1
D-1	1	D-1.1
Total	32	19
NA		
1E-1	1	ND
1E-2	2	1E -2.1, 2.2
2D-1	3	2D-1.1, 1.3
2D-2	1	2D-2.1
2D-3	1	2D-3.1
2G-1	2	2G-1.1, 1.2
3A-1	1	ND
3A-2	2	3A-2.1, 2.2
3D-1	1	ND
4A-1	1	4A-1.1
6A-1	3	6A-1.1, 1.2
6A-2	1	ND
6A-3	4	6A-3.2
6A-4	3	ND
8A-1	4	ND
8A-2	2	ND
8A-3	1	ND
9A-1	3	ND
9A-2	1	ND
9A-3	1	ND
Total	38	14
M		
E-1	21	E-1.1, 1.2, 1.3,1.5 to 1.21
E-2	1	E-2.1
J-1	1	E-3.1
Total	23	22
NS		
1D-1	13	1D-1.1, 1.2, 1.4, 1.5, 1.7, 1.8, 1.9, 1.10, 1.12

1C-1	1	1C-1.1
2B-1	8	2B-1.1 to 1.8
2B-2	1	2B-2.1
Total	23	18
Overall totals	248	142

[a] Not detected
