# Supplementary material for: Genetic Structure of Avian Influenza Viruses from Ducks of the Atlantic Flyway of North America
Source: PLoS One. 2014 Jan 30;9(1):e86999. doi: 10.1371/journal.pone.0086999 (PMC3907406; doi:10.1371/journal.pone.0086999)
Supplement: Table S9 — Identification of Atlantic Flyway duck AIV gene types in hosts other than ducks. (PDF) [file pone.0086999.s014.pdf]

Table S9. Identification of Atlantic Flyway duck AIV gene types in hosts other than ducks.

Segment	Gene type	Hosts
PB2	C-2.2	shorebird
	C-2.3	shorebird, goose, turkey, chicken
	C-2.5	goose
	C-2.7	shorebird
	C-2.12	chicken
	C-2.13	shorebird
	C-2.15	turkey, quail
	C-2.16	chicken
	C-2.19	shorebird
	C-3.1	shorebird, goose, turkey, swan
	C-3.5	shorebird
	C-4.1	murre
	C-5.1	chicken, guinea fowl, pheasant
	J-1.1	gull
PB1	F-1.3	shorebird, goose
	F-1.4	shorebird, chicken, gull
	F-2.1	turkey, dove
	F-3.2	shorebird
	F-3.3	shorebird
	F-3.4	shorebird
	F-3.5	shorebird
	F-3.6	shorebird
	F-3.7	shorebird
	F-3.9	shorebird, turkey
	F-3.10	turkey, chicken, guinea fowl, pheasant, quail
	F-4.5	goose
	F-4.7	goose, grebe
	F-5.1	shorebird, turkey, quail
	F-5.2	shorebird, turkey, quail
	F-6.1	shorebird, swan
	F-6.2	turkey, swan
	F-8.1	gull
PA	H-1.1	shorebird, chicken, gull, murre
	H-1.4	shorebird, gull
	H-1.8	turkey, chicken, guinea fowl, pheasant, quail
	H-1.9	goose, swan
	H-1.11	shorebird
	H-1.12	goose, swan
	H-1.13	goose
	H-1.14	shorebird, goose
	H-1.16	goose
	H-1.17	shorebird
	E-1.1	dove
	E-1.4	goose, chicken, coot

	E-1.5	goose, chicken
	E-2.1	shorebird
	E-2.2	shorebird, goose
	E-3.1	shorebird, gull, murre
	E-3.2	shorebird, turkey
	E-3.4	shorebird
	E-5.1	shorebird, turkey, quail
	E-5.2	shorebird, turkey
	E-6.1	gull
	E-6.2	shorebird, gull
HA	1D-1.1	dove, dunlin
	2H-1.2	chicken
	3C-1.2	shorebird
	3D-1.1	shorebird
	4A-1.1	shorebird
	4A-1.4	shorebird
	4A-3.2	shorebird
	5C-1.2	goose, turkey, swan
	5C-1.3	chicken
	5C-1.4	goose, turkey, swan
	5C-1.5	goose, turkey
	6B-1.1	shorebird
	7F-2.1	turkey, chicken, guinea fowl, quail
	13A-1.1	gull
NA	1E- 2.1	shorebird, goose
	1E- 2.2	shorebird, goose
	2D-1.1	dove
	2D-1.3	goose, chicken
	2D-2.1	shorebird
	2D-3.1	shorebird, goose, turkey
	2G-1.1	turkey, chicken, guinea fowl, pheasant, chukar
	2G-1.2	shorebird, goose, turkey
	3A-2.1	shorebird
	3A-2.2	chicken
	4A-1.1	shorebird
	6A-1.1	swine
	6A-1.2	gull
	6A-3.2	shorebird
NP	F-1.1	swine
	H-1.2	shorebird, goose, turkey, chicken, chukar
	H-1.4	shorebird, goose, chicken
	H-1.5	shorebird, goose
	H-1.6	goose, chicken, swan
	H-1.7	shorebird, goose, turkey, chicken
	H-2.3	shorebird
	H-2.4	goose, dove

	H-2.5	shorebird, goose, swan
	H-3.1	shorebird
	H-3.4	shorebird
	H-4.3	turkey, quail
	H-4.4	chicken
	H-4.5	chicken
	H-4.6	chicken
	H-5.2	shorebird
	H-6.2	shorebird, grebe
	H-7.1	chicken, guinea fowl, pheasant
	D-1.1	shorebird, gull
M	E-1.1	dove
	E-1.2	cormorant
	E-1.3	shorebird, turkey, chicken
	E-1.5	goose, chicken
	E-1.6	shorebird, turkey, gull, grebe, cormorant
	E-1.7	shorebird, turkey, cormorant
	E-1.8	shorebird, goose, murre, swine
	E-1.9	shorebird, turkey, chicken, gull, quail, swine
	E-1.10	shorebird, turkey, murre
	E-1.11	shorebird, chicken, pheasant
	E-1.13	shorebird, goose, turkey, grebe, cormorant
	E-l.12	shorebird, chicken, murre, swine
	E-1.14	shorebird, swine
	E-1.15	shorebird
	E-1.16	turkey, swan, dunlin
	E-1.17	shorebird, turkey, quail, murre, swine
	E-1.18	shorebird, goose
	E-1.19	shorebird, turkey, chicken, guinea fowl, rhea
	E-1.20	goose
	E-1.21	turkey, chicken
	E-2.1	turkey, chicken, guinea fowl, pheasant, quail
	J-1.1	shorebird, gull
NS	1D-1.1	shorebird, turkey, chicken, coot
	1D-1.2	goose, coot
	1D-1.4	shorebird, chicken
	1D-1.5	goose, chicken
	1D-1.7	shorebird, goose, turkey, swan, swine
	1D-1.8	shorebird, goose, turkey, guinea fowl, swine
	1D-1.9	shorebird, goose, turkey, chicken
	1D-1.10	shorebird
	1D-1.12	shorebird
	1C-1.1	gull
	2B-1.1	gull
	2B-1.2	shorebird, goose, murre
	2B-1.3	shorebird, gull

2B-1.4	gull
2B-1.5	gull
2B-1.6	goose, guinea fowl, murre
2B-1.7	shorebird
2B-1.8	shorebird, swan
2B-2.1	turkey, chicken, guinea fowl, pheasant, quail
